# The Repertoire of Solute-Binding Proteins of Model Bacteria Reveals Large Differences in Number, Type, and Ligand Range

**DOI:** 10.1128/spectrum.02054-22

**Published:** 2022-09-19

**Authors:** Álvaro Ortega, Miguel A. Matilla, Tino Krell

**Affiliations:** a Department of Biochemistry and Molecular Biology B and Immunology, Faculty of Chemistry, University of Murcia, Regional Campus of International Excellence Campus Mare Nostrum, Murcia, Spain; b Department of Environmental Protection, Estación Experimental del Zaidín, Consejo Superior de Investigaciones Científicas, Granada, Spain; South China Sea Institute of Oceanology

**Keywords:** solute-binding proteins, transport, signal transduction, model bacteria, transport substrate, signal molecules, transmembrane receptors

## Abstract

Solute-binding proteins (SBPs) are of central physiological relevance for bacteria. They are located in the extracytosolic space, where they present substrates to transporters but also stimulate different types of transmembrane receptors coordinating compound uptake with signal transduction. SBPs are a superfamily composed of proteins recognized by 45 Pfam profiles. The definition of SBP profiles for bacteria is hampered by the fact that these Pfam profiles recognize sensor domains for different types of signaling proteins or cytosolic proteins with alternative functions. We report here the retrieval of the SBPs from 49 bacterial model strains with different lifestyles and phylogenetic distributions. Proteins were manually curated, and the ligands recognized were predicted bioinformatically. There were very large differences in the number and type of SBPs between strains, ranging from 7 SBPs in Helicobacter pylori 26695 to 189 SBPs in Sinorhizobium meliloti 1021. SBPs were found to represent 0.22 to 5.13% of the total protein-encoding genes. The abundance of SBPs was largely determined by strain phylogeny, and no obvious link with the bacterial lifestyle was noted. Most abundant (36%) were SBPs predicted to recognize amino acids or peptides, followed by those expected to bind different sugars (18%). To the best of our knowledge, this is the first comparative study of bacterial SBP repertoires. Given the importance of SBPs in nutrient uptake and signaling, this study enhances the knowledge of model bacteria and will permit the definition of SBP profiles of other strains.

**IMPORTANCE** SBPs are essential components for many transporters, but multiple pieces of more recent evidence indicate that the SBP-mediated stimulation of different transmembrane receptors is a general and widespread signal transduction mechanism in bacteria. The double function of SBPs in coordinating transport with signal transduction remains to a large degree unexplored and represents a major research need. The definition of the SBP repertoire of the 49 bacterial model strains examined here, along with information on their cognate ligand profiles forms the basis to close this gap in knowledge. Furthermore, this study provides information on the forces that have driven the evolution of transporters with different ligand specificities in bacteria that differ in phylogenetics and lifestyle. This article is also a first step in setting up automatic algorithms that permit the large-scale identification of the SBP repertoire in proteomes.

## INTRODUCTION

Bacterial life is enabled by the uptake of compounds from their environment. To this end, bacteria have evolved different types of transmembrane transporters that permit the specific uptake of a variety of organic and inorganic compounds. Several transporter families, like the ATP-binding cassette (ABC), tripartite ATP-independent periplasmic (TRAP), and tripartite tricarboxylate transporter (TTT) families, employ solute-binding proteins (SBPs) to capture the transport substrate in the extracytosolic space and to present it to the transmembrane receptor permeases (1). These proteins thus play a central role in defining the transporter substrate specificity.

Whereas SBPs in Gram-negative bacteria are present as diffusible proteins in the periplasm, they are tethered to the external face of the cytoplasmic membrane in Gram-positive bacteria and archaea (2). SBPs are found in all kingdoms of life (1) and form a superfamily composed of many protein families (3). Based on an analysis of their structural distance, SBPs have been classified into 6 or 7 clusters (1, 4, 5). Although SBPs vary greatly in size, from 20 to 65 kDa, they share the same overall topology, which consists of two lobes linked by a hinge region (5–7). The transport substrate binds to the interface of both lobes, a process that frequently induces structural rearrangements (8, 9).

Other major constituents of prokaryotic membranes are signal transduction receptors (10). These proteins sense extracytoplasmic signals to define a cellular response, leading to a more optimal adaptation to a given environmental condition. The most abundant signal transduction receptors are sensor kinases, chemoreceptors, diguanylate cyclases and phosphodiesterases, adenylate cyclases, extracytosolic function sigma factors, and Ser/Thr/Tyr kinases and phosphatases (10, 11). Many of these receptors are transmembrane proteins that contain an extracytoplasmic ligand-binding domain (LBD) that is flanked by two transmembrane regions. Signal binding to the LBD creates a molecular stimulus that is transmitted to the cytosolic part of the receptor, inducing signaling cascades for the definition of a response.

Transmembrane receptors employ many different LBD types for signal sensing (12, 13), as exemplified by the more than 80 different LBD types identified in chemoreceptors (12). Typically, transmembrane receptors are stimulated by the direct binding of signal molecules to LBDs, but there is an alternative mechanism for receptor activation that consists of the binding of ligand-loaded SBPs to signal transduction receptor LBDs. Several pieces of evidence suggest that this is a general and widespread mechanism: (i) different receptor types can be activated by SBP binding, including chemoreceptors (14, 15), sensor kinases (16, 17), diguanylate cyclases/cyclic di-GMP (c-di-GMP) phosphodiesterases (18, 19) or serine/threonine kinases (20, 21); (ii) this mechanism of receptor stimulation has a wide phylogenetic spread in archaea and Gram-positive and Gram-negative bacteria (22); (iii) a significant number of different receptor LBD types were found to bind SBPs (22); (iv) SBPs that belong to at least 13 families were found to bind to receptor LBDs (22); and (v) the most extensively studied chemoreceptors are the Escherichia coli proteins, and there is evidence that all four receptors with a periplasmic LBD can be activated by SBP binding (14, 23–25).

There is scarce information available on the number and type of SBPs present in different bacterial strains, information that forms the basis for establishing eventual links with bacterial physiology, lifestyle, and habitat. However, establishing the SBP repertoire of bacterial strains is hampered by a number of issues: (i) there are at least 45 different SBP Pfam families, most of which have been regrouped into 2 clans (CL0144 and CL0177) (26); (ii) there are SBP genes that are not in the vicinity of transporter genes (27); (iii) many domains recognized by Pfam signatures as SBPs are sensor domains of other signal transduction receptors, like sensor kinases or transcriptional regulators (26); and (iv) there are cytosolic single-domain proteins that carry out alternative functions and that are also recognized by Pfam SBP signatures (28).

Proteins of the SBP superfamily recognize a very diverse range of ligands, including amino acids, sugars, organic acids, polyamines, metal cations and oxyanions, inorganic anions, quaternary amines, peptides, and phosphonates (7, 27). Remarkably, the recent classification of SBPs into 7 clusters based on structural phylogenetics made it possible to establish a correlation between SBP clusters and ligand specificity (5). In addition, the TransportDB database, which was developed to annotate transport systems in sequenced genomes, contains an algorithm that predicts the ligands recognized by transport systems and their cognate SBPs (27). In this regard, in a previous study we assessed the precision of these TransportDB predictions. We purified 17 SBPs and determined their ligand profile experimentally, showing that the experimental data matched, to a large degree, the predictions by the TransportDB database (29).

In the present study, we developed an algorithm that identifies members of the SBP superfamily in bacterial strains. We used this algorithm to define the SBP repertoire of 49 model strains that show a wide phylogenetic spread and that differ in lifestyle and habitat. Our results reveal very large differences in the number and type of SBPs between strains. No obvious links between the bacterial lifestyles and the SBP repertoire were observed. In a number of cases, phylogenetically close bacteria with different lifestyles showed similar SBP repertoires.

## RESULTS

### Selection of model strains and data retrieval.

To compile and analyze SBPs, we selected 49 bacterial strains. The primary criteria for the selection were (i) their relevance in microbiological research, (ii) their phylogenetic distribution, (iii) their diversity in lifestyles and ecological niches, and (iv) the availability of the complete proteome in UniProtKB (30). These strains were then classified according to their lifestyle, ecological habitat, and isolation source into 11 groups, namely, sediment (marine/fresh water), human/animal pathogen, freshwater/marine water, soil, human intestinal microflora, food, oil reservoir, active sludge, plant pathogen, beneficial plant associated, and animal symbiont (Table 1). More detailed information on the classification of these different strains is found in Table S1 in the supplemental material. We then compiled a list of Pfam families that were shown or predicted to function as SBPs. The Pfam codes of the resulting 45 families are provided in Materials and Methods. We subsequently retrieved all proteins that match these Pfam signatures in the 49 proteomes. We found that many members of these 45 Pfam domain families were not SBPs but proteins with cytosolic location or sensor domains of cytosolic and transmembrane signal transduction systems. To distinguish between SBPs and proteins with alternative functions in this initial set of retrieved sequences, we identified those sequences (i) for which the protein segment covered by the SBP domain profile is less than 60% of the total protein length, (ii) which had predicted transmembrane regions, and (iii) which lacked a predicted signal peptide. These sequences were then curated manually using a number of approaches defined in Materials and Methods. A flowchart of the individual steps leading to the establishment of the SBP repertoire is shown in Fig. S1.

**TABLE 1:** Assignation of a bacterial lifestyle and ecological habitat to the different bacterial strains analyzed in this study^*a*^

Strain	Lifestyle/isolation source
Thermotoga maritima MSB8	Sediments (marine water/freshwater)
Chlamydia trachomatis D/UW-3/CX	Human/animal pathogen
Borrelia burgdorferi B31	Human/animal pathogen
Spirochaeta thermophila Z-1203 (=DSM 6578)	Freshwater/marine water
*Synechocystis* sp. strain PCC 6803	Freshwater/marine water
Anabaena cylindrica PCC 7122	Freshwater/marine water
Streptomyces coelicolor A3(2)	Soil
Nocardia brasiliensis ATCC 700358	Human/animal pathogen
Mycobacterium tuberculosis H37Rv	Human/animal pathogen
Clostridium botulinum A strain ATCC 3502	Human/animal pathogen
Lactobacillus acidophilus NCFM	Human intestinal microflora
Lactococcus lactis subsp. *lactis* Il1403	Food
Streptococcus pneumoniae R6	Human/animal pathogen
Staphylococcus aureus NCTC 8325	Human/animal pathogen
Geobacillus thermodenitrificans NG80-2	Oil reservoir
Bacillus amyloliquefaciens FZB42	Soil
Bacillus subtilis subsp. *subtilis* 168	Soil
Bdellovibrio bacteriovorus HD100	Soil
Helicobacter pylori 26695	Human/animal pathogen
Neisseria meningitidis MC58	Human/animal pathogen
Bordetella pertussis Tohama I	Human/animal pathogen
Comamonas testosteroni CNB-2	Active sludge
Burkholderia cepacia 383	Soil
Ralstonia solanacearum GMI1000	Plant pathogen
Sphingomonas wittichii RW1	Freshwater/marine water
Caulobacter crescentus CB15	Freshwater/marine water
Rhodobacter sphaeroides ATCC 17025	Freshwater/marine water
Azospirillum baldaniorum Sp245^T^ (formerly Azospirillum brasilense Sp245^T^)	Beneficial plant associated
Brucella abortus 2308	Human/animal pathogen
*Sinorhizobium* (*Ensifer*) *meliloti* 1021	Beneficial plant associated
*Agrobacterium fabrum* C58	Plant pathogen
Acinetobacter baumannii AB0057	Human/animal pathogen
Xanthomonas campestris pv. *campestris* ATCC 33913	Plant pathogen
Legionella pneumophila subsp. *pneumophila* strain Philadelphia-1	Human/animal pathogen
Shewanella oneidensis MR-1	Sediments (marine water/freshwater)
Aliivibrio fischeri MJ11	Animal symbiont
Vibrio cholerae O1 biovar El Tor strain N16961	Human/animal pathogen
Azotobacter vinelandii DJ	Soil
Pseudomonas syringae pv. *tomato* DC3000	Plant pathogen
Pseudomonas fluorescens Pf0-1	Soil
Pseudomonas putida KT2440	Soil
Pseudomonas aeruginosa PAO1	Human/animal pathogen
Pectobacterium atrosepticum SCRI1043	Plant pathogen
Photorhabdus luminescens subsp. *laumondii* TTO1	Human/animal pathogen
Yersinia pestis Nepal516	Human/animal pathogen
Serratia plymuthica S13	Beneficial plant associated
Salmonella enterica serovar Typhimurium LT2	Human/animal pathogen
Escherichia coli MG1655	Human intestinal microflora
Klebsiella pneumoniae HS11286	Human/animal pathogen

a The strains are ordered according to their phylogenetic relationship, as shown in Fig. 3. More detailed information on lifestyles and habitats is found in Table S1.

### Correlation between SBP families and ligand specificity: the central role of amino acid- and sugar-sensing SBPs.

**FIG 1:**
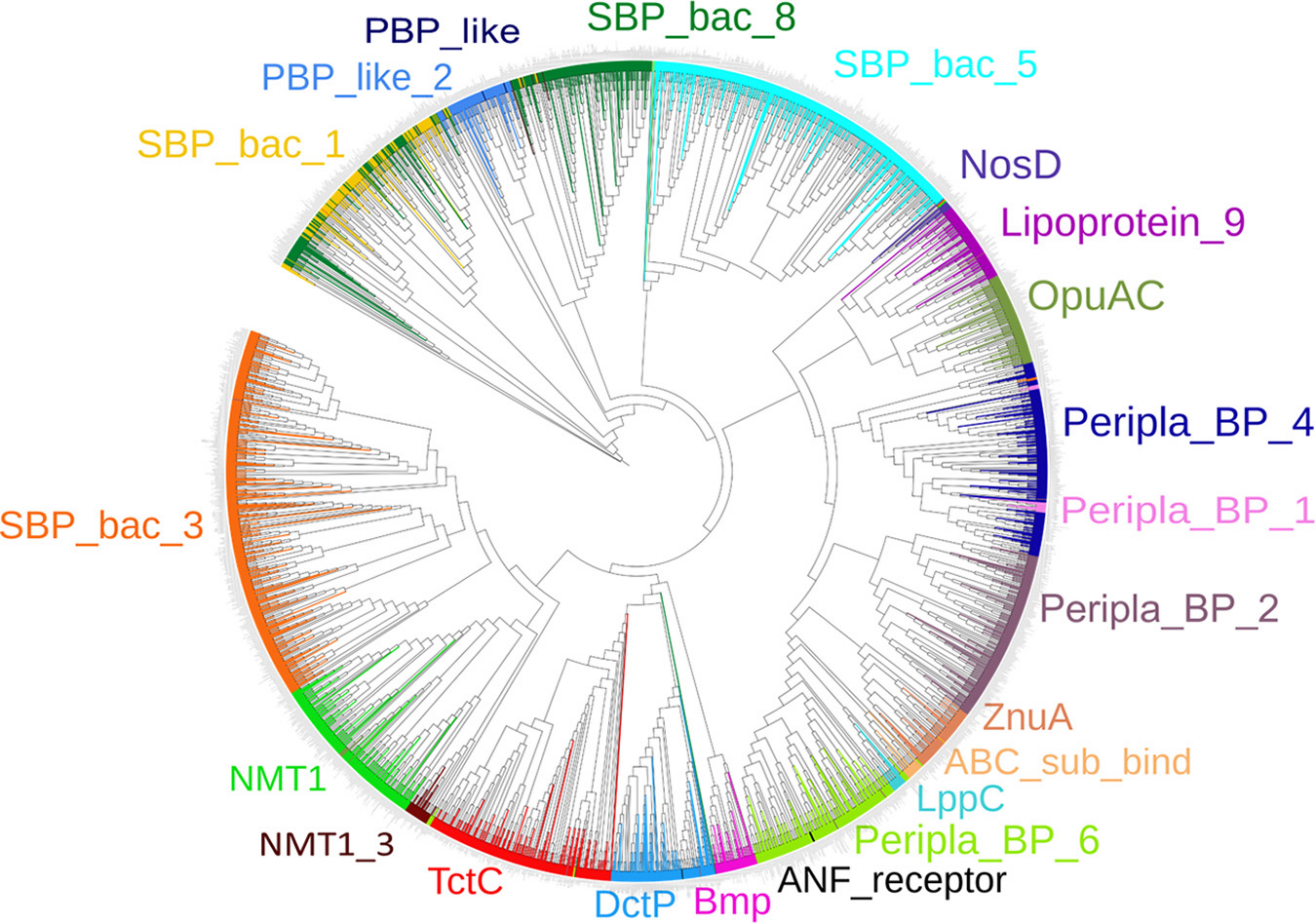
Unrooted phylogenetic tree of the solute-binding proteins present in the 49 bacterial strains analyzed in this study. Proteins are colored according to their Pfam families.

**FIG 2:**
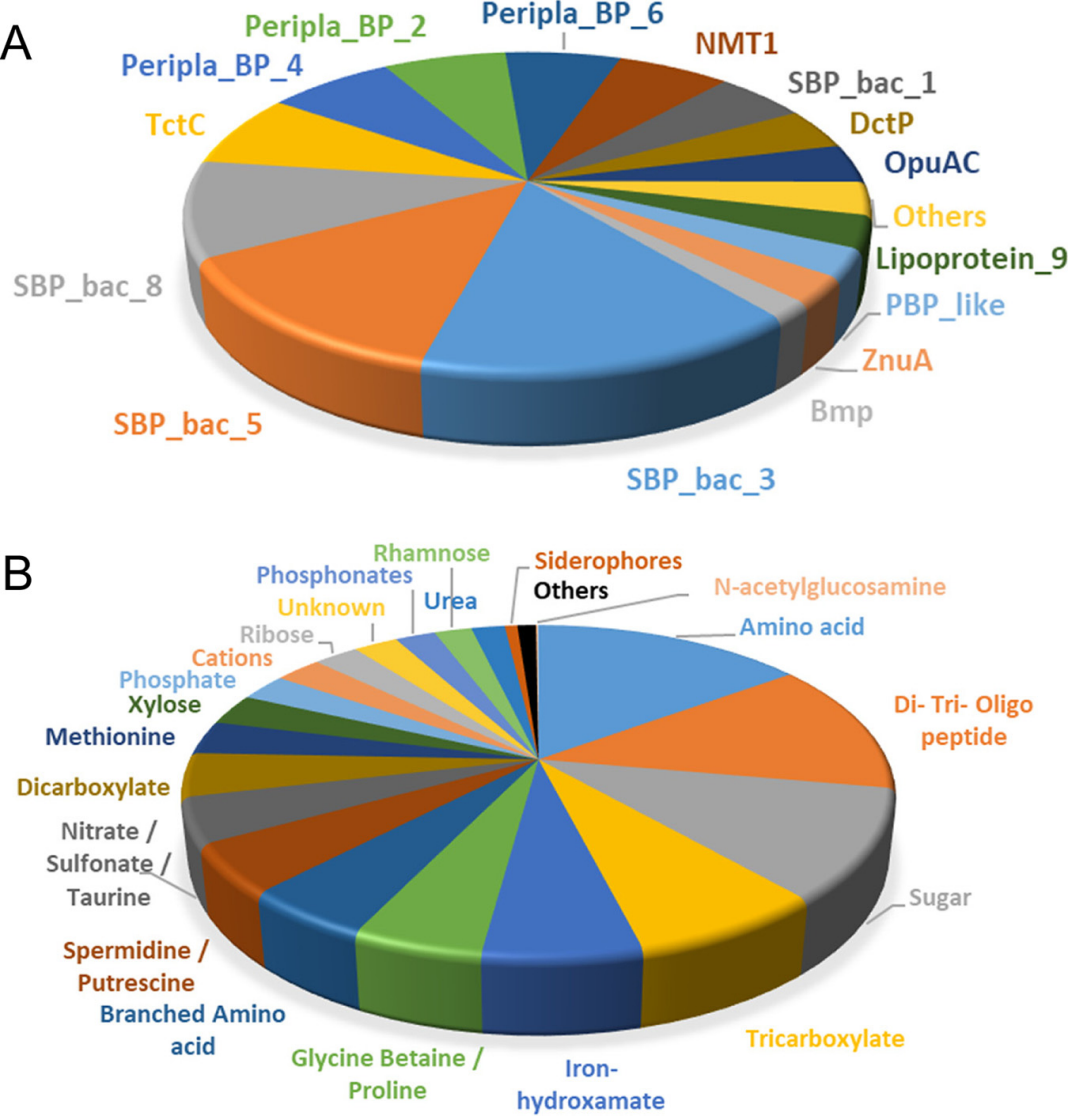
Classification of the solute-binding proteins from 49 strains according to their Pfam families (A) and ligands recognized as predicted by TransportDB (B). Less populated Pfam families and ligand categories have been regrouped in the category “Others.”

The final curated SBP repertoire of the 49 strains is found in Table S2. In total, these 49 strains contained 2,934 SBPs that belonged to 22 protein families (Table 2). Figure 1 shows the phylogenetic tree of the SBPs; sequences are colored according to their respective Pfam protein family. Whereas members of most families cluster together, members of the families SBP_bac_1, PBP_like_2, PBP_like, and SBP_bac_8 were mixed. The ligands predicted to be recognized for each of the proteins are provided in Table S2, and Table 2 summarizes the ligands predicted to be recognized by members of the individual SBP families. A number of conclusions can be derived from these data. (i) There is a clear correlation between the SBP family and the ligands recognized, since most families are predicted to recognize a particular ligand type. Ten families were predicted to bind a single ligand type (Table 2), including the well-populated families SBP_bac_3, TctC, and DctP, which were predicted to bind specifically only amino acids, tricarboxylates, and dicarboxylates, respectively. Another seven families were predicted to bind 2 or 3 ligand types. The data thus indicate that the type of ligand recognized is reflected in the protein sequence. (ii) Most populated were the SBP_bac_3 and SBP_bac_5 families (Table 2; Fig. 2). Members of these families have been predicted to bind almost exclusively amino acids or di-, tri-, and oligopeptides, respectively. In addition, the well-populated families Peripla_BP_6 and Lipoprotein_9 were predicted to bind mostly branched-chain amino acids and methionine, respectively (Table 2). Taken together, the SBPs predicted to bind amino acids or peptides amount to more than 36% of the total number of SBPs retrieved in this study, indicating a particular relevance in the uptake and sensing of these compounds. (iii) The second most abundant ligand family is sugars. Almost all members of the families SBP_bac_1 and Bmp were predicted to bind sugars, and the family Peripla_BP_4 is specific for xylose, rhamnose, and ribose (Table 2; Fig. 2). In total, 18% of all SBPs are predicted to bind sugars. (iv) Other populated ligand groups were tricarboxylates (7.5%), iron hydroxamate (6.1%), the quaternary amines betaine and proline (5%), the polyamines spermidine and putrescine (4.8%), the nitrate-sulfonate-taurine category (4.4%), and dicarboxylates (4.3%), which are recognized mostly by members of the TctC, Peripla_BP_2, OpuAC, SBP_bac_8, NMT1, and DctP families, respectively (Table 2; Fig. 2).

**TABLE 2:**
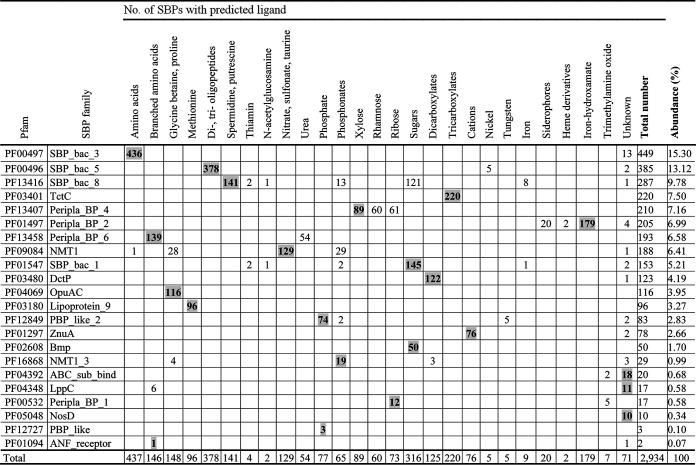
Prediction of the ligands recognized by the solute binding proteins from the 49 model strains according to TransportDB (27)^*a*^

a Values for the primary ligands for each family are in boldface and shading.

### Bioinformatic predictions by TransportDB strongly correlate with experimentally determined SBP ligands.

To assess the precision of ligand prediction by TransportDB, we extracted from the UniProt and Protein Data Bank (PDB) databases all articles (435) and three-dimensional (3D) structures (~600) associated with the 2,934 SBP sequences initially retrieved. After the inspection of these articles and protein structures, we were able to retrieve information on the ligands recognized by 166 SBPs. These data, along with the article references and PDB identification codes, are provided in Table S2. The analysis revealed that 71% of the ligands were predicted correctly by TransportDB. Furthermore, in 10% of the proteins, TransportDB predicted a single ligand, and the experimentally determined ligand belonged to the same family as the predicted ligand. For another 4%, the experimentally determined ligand was similar to the family of predicted ligands. Remarkably, TransportDB predicted the ligand incorrectly for only 15% of the proteins. Taken together, these data indicate an elevated precision of TransportDB-based ligand predictions.

### Strains differ widely in the number and type of SBPs as well as in the ligands recognized.

**FIG 3:**
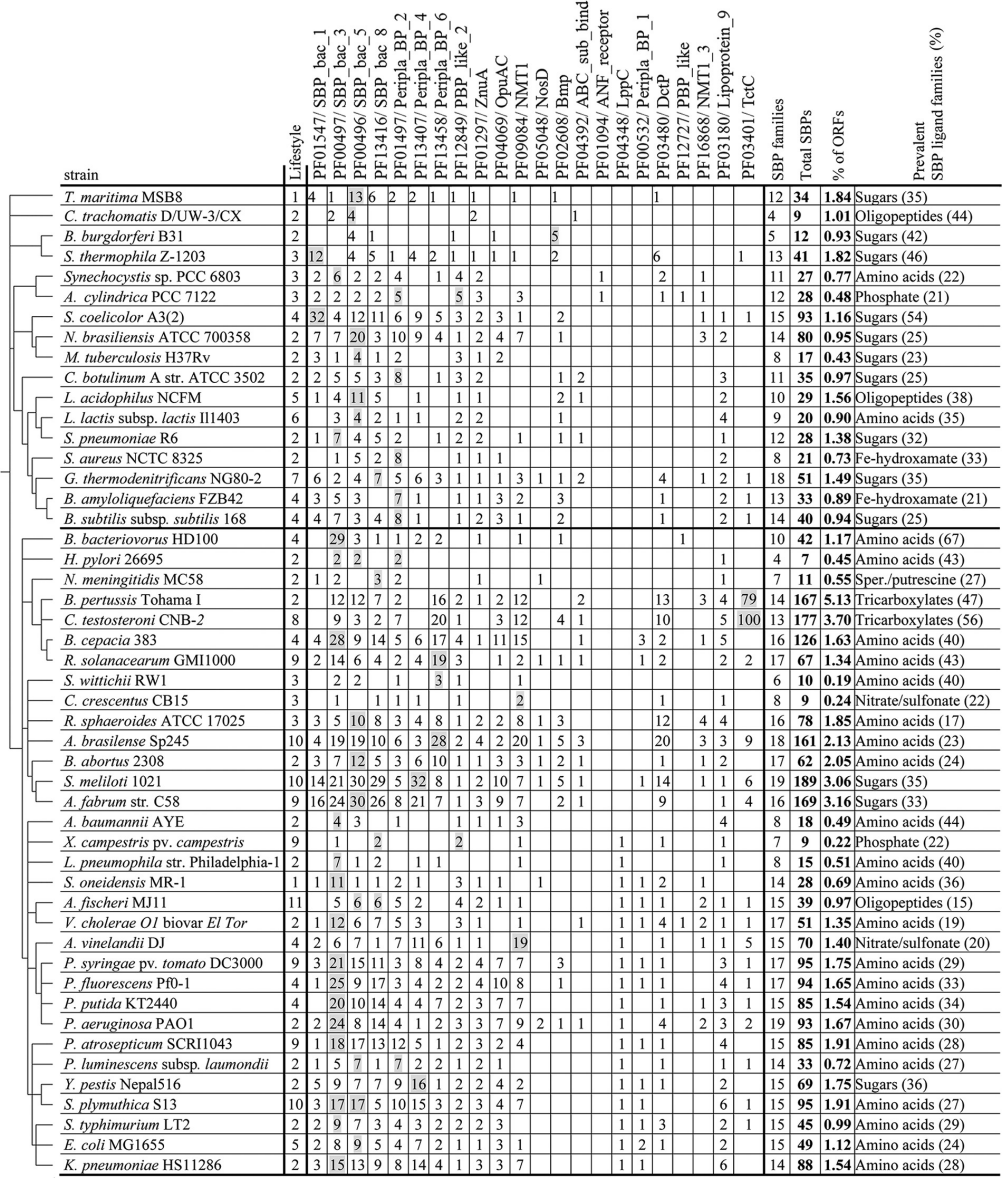
The solute-binding protein repertoires of 49 bacterial model strains. Members of the most abundant protein family for each strain are shaded. The second column indicates the bacterial lifestyle/ecological niche as detailed in Table S1. Lifestyles were classified as follows: 1, sediment (marine water/freshwater); 2, human/animal pathogen; 3: freshwater/marine water; 4, soil; 5, human intestinal microflora; 6, food; 7, oil reservoir; 8, active sludge; 9, plant pathogen; 10, beneficial plant associated; 11, animal symbiont. The ligand prediction was done using the TransportDB database (27), and ligands of the individual families correspond to the categories shown in Fig. 2B. In the last column, showing the most abundant ligand recognized by the SBPs of a given strain, “amino acids” corresponds to the sum of the categories “amino acids,” “branched amino acids,” and “methionine,” whereas “sugars” corresponds to the sum of “sugars,” “xylose,” “ribose,” and “rhamnose,” as detailed in Table 2 and Fig. 2B. The upper and lower branches of the phylogenetic tree are separated by a thick line.

**FIG 4:**
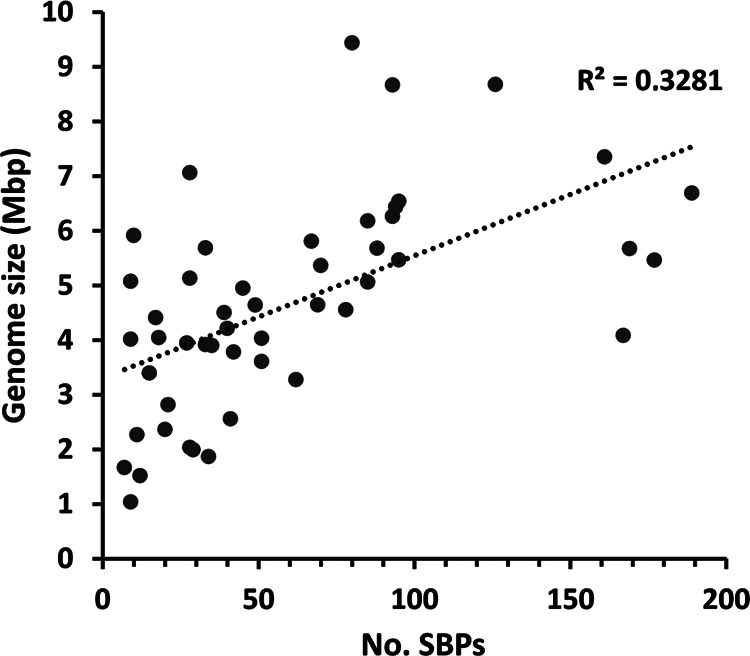
Abundance of solute binding proteins as a function of genome size in the 49 model bacterial strains. For the calculation of genome size, the presence of plasmids was considered.

On average, the strains analyzed contained 60 ± 49 SBPs. However, their abundance varied largely among strains (Fig. 3). Strains that contain very few SBPs include Helicobacter pylori 26695 (7 SBPs), Chlamydia trachomatis D/UW-3/CX (9 SBPs), Caulobacter crescentus CB15 (9 SBPs) and Xanthomonas campestris pv. *campestris* ATCC 33913 (9 SBPs), whereas the highest numbers were observed in Sinorhizobium meliloti 1021 (189 SBPs), Comamonas testosteroni CNB-2 (177 SBPs), Agrobacterium fabrum C58 (169 SBPs), and Bordetella pertussis Tohama I (167 SBPs). There was thus a 27-fold difference between the strains with most and fewest SBPs. The genomes of the strains analyzed differ significantly in size, from 1.042 Mbp (C. trachomatis D/UW-3/CX) to 9.436 Mbp (Nocardia brasiliensis ATCC 700358), and we found no correlation between the size of their genomes and the total number of SBPs (Fig. 4). In accordance, Fig. 3 shows the percentage of SBPs with respect to the total number of open reading frames (ORFs). The highest percentage of SBPs relative to the total number of ORFs was observed in Bordetella pertussis Tohama I (5.13%), whereas the lowest percentage was obtained with 0.19% in Sphingomonas wittichii RW1, resulting in a 27-fold difference (Fig. 3).

**FIG 5:**
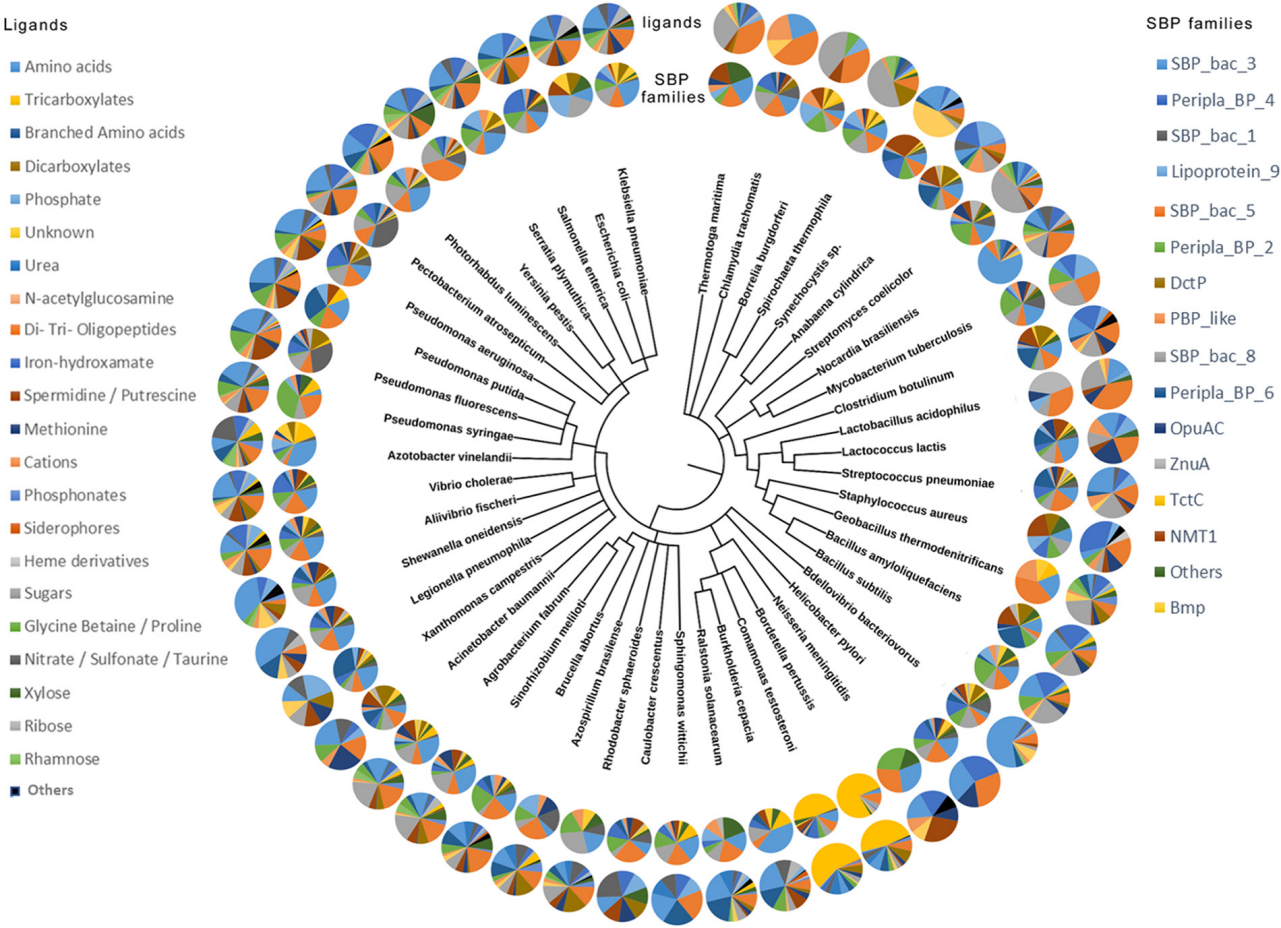
Phylogenetic tree of the 49 strains analyzed. The inner circle corresponds to the abundance of the SBP families for each strain, whereas the outer circle represents the abundance of ligands predicted for the SBPs of each strain.

Figure 5 presents a phylogenetic tree of the 49 strains analyzed in this work, including the relative abundance of the different SBP families and the predicted abundance of ligands for each strain. Strains can be distinguished that possess a balanced SBP ligand profile, whereas others possess a strong bias for a particular ligand family, caused mainly by a particularly abundant SBP family. E. coli MG1655 is an example of a strain with a balanced repertoire. It has 49 SBPs, which is close to the average number of SBPs in the strains analyzed in this study, and SBPs are distributed over 15 different families. It has a modest degree of redundancy, with 9, 8 and 7 members of the PF00496 (SBP_bac_5), PF00497 (SBP_bac_3), and PF13407 (Peripla_BP_4) families, which are specific for peptides, amino acids and sugars, respectively. This balanced SBP profile thus permits the sensing of a broad range of ligands. Other strains possess a SBP repertoire that is strongly biased toward the recognition of a particular ligand type. For example, 69% of Bdellovibrio bacteriovorus HD100 SBPs belonged to a single family, namely, the amino acid-specific family PF00497 (SBP_bac_3) (Table 2). B. bacteriovorus has a particular lifestyle, since it is a bacteriolytic ectoparasite that predates Gram-negative bacteria (31). Another example of an unbalanced SBP profile is the enormous abundance of TctC family members in the phylogenetically close B. pertussis Tohama I (79 members) and *C. testosteroni* CNB-2 (100 members) (Fig. 3). This abundance is in stark contrast to the remaining strains, which had either no or only 1 to 9 TctC family members (Fig. 3). All 220 TctC family members analyzed were predicted to bind tricarboxylates (Table 2; Table S2). Other examples of bacteria with unbalanced SBP profiles are Streptomyces coelicolor A3(2), Spirochaeta thermophila Z-1203, and Borrelia burgdorferi B31 for which about half of their SBPs were predicted to bind sugars, or the elevated abundance of peptide-binding SBPs in the phylogenetically close Thermotoga maritima MSB8, C. trachomatis D/UW-3/CX and B. burgdorferi B31. It remains to be established whether and to what degree the lifestyle is related to this ligand bias.

### The SBP repertoire is primarily related to phylogeny.

**FIG 6:**
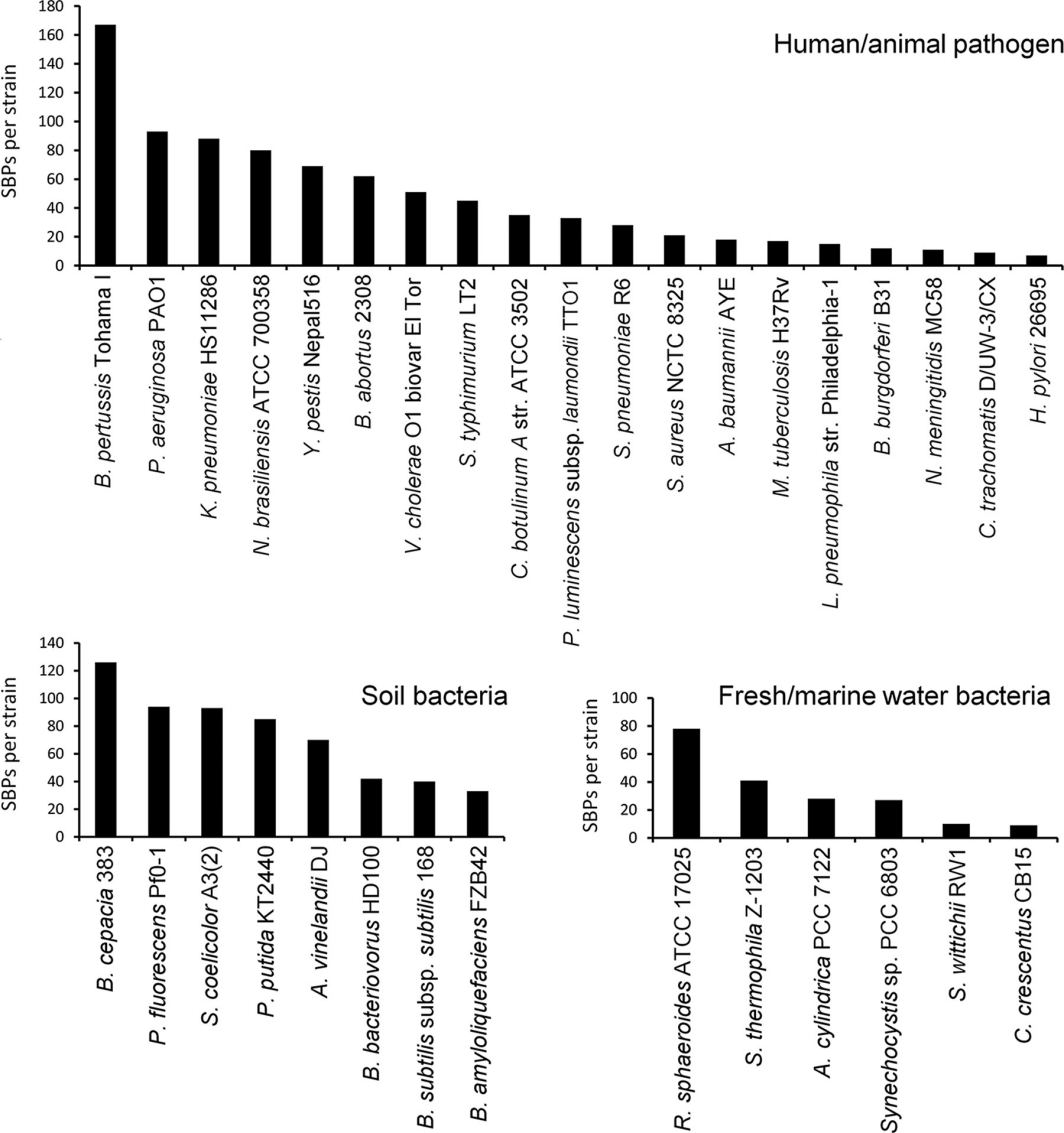
Abundance of solute-binding proteins in strains with different lifestyles. Shown are the abundances of proteins for strains that were classified as human/animal pathogens, soil bacteria, or freshwater/marine water bacteria.

Considering the large differences between strains in the total number of SBPs, the SBP profiles, and the diversity of ligands that these SBPs recognize, we investigated whether SBP abundance is a reflection of bacterial lifestyle. The analysis of the SBP abundance in bacteria with different lifestyles did not show any obvious correlation. This is illustrated by the three most populated lifestyle categories, namely, human/animal pathogens, soil bacteria, and freshwater/marine water bacteria (Fig. 6). The SBP abundance in human/animal pathogens spanned almost the entire range from 167 SBPs in B. pertussis to only 7 SBPs in H. pylori 26695. A similarly wide spread was observed for soil bacteria, which had between 126 (Burkholderia cepacia 383) and 33 (Bacillus amyloliquefaciens FZB42) SBPs, and freshwater/marine water bacteria, which harbored between 78 (Rhodobacter sphaeroides ATCC 17025) and 9 (C. crescentus CB15) SBPs (Fig. 6). Similarly, large differences were observed in the plant pathogens X. campestris pv. *campestris* ATCC 33913 (9 SBPs), Ralstonia solanacearum GMI1000 (67 SBPs), Pectobacterium atrosepticum SCRI1043 (85 SBPs), and Pseudomonas syringae pv. tomato DC3000 (95 SBPs).

Although the evolutionary mechanisms that have led to the differences in SBP content and abundance between strains are unknown, there are a number of observations indicating that phylogenetic closeness of strains rather than similarities in the lifestyle is related to the SBP repertoire. Thus, the phylogenetic tree in Fig. 3 can be divided into two main branches, with the members of the upper branch having a lower SBP content (35 ± 22 SBPs) than the lower branch (73 ± 53 SBPs). The last column in Fig. 3 shows the most abundant ligand family for each of the strains. In this analysis, the ligand categories shown in Fig. 2B “amino acids,” “branched amino acids” and “methionine” were combined into a single category called “amino acids,” and the categories “sugars,” “xylose,” “ribose” and “rhamnose” were combined into the category “sugars”. For almost half (i.e., 24) of the analyzed strains, amino acids formed the most populated ligand group, followed by 13 strains for which sugars were the predominant SBP ligand (Fig. 3). For both categories, a clear link with strain phylogenetics was observed. In the upper branch of the phylogenetic tree (Fig. 3), amino acids were most abundant for only 11.8% of the strains, whereas this ligand family was prevalent for 68.8% of the strains in the lower branch. Conversely, 58.8% of the strains in the upper branch preferentially bound sugars, which is in stark contrast to the only 9.4% of the strains in the lower branch.

In a number of cases, species on neighboring phylogenetic branches showed a similar number of total SBPs and a similar spread over the individual SBP families (Fig. 3). A first illustrative example is B. pertussis and *C. testosteroni*, two species that belong to the order *Burkholderiales* but that differ radically in lifestyle. Whereas B. pertussis Tohama I is a human pathogen that infects airways, causing whooping cough (32), *C. testosteroni* CNB-2 is a free-living bacterium, isolated from activated sludge, that is able to metabolize complex xenobiotic compounds (33). Despite these differences, the number and distribution of their SBPs are very similar (Fig. 3). B. pertussis Tohama I and *C. testosteroni* CNB-2 possess 167 and 177 SBPs, respectively, which are distributed similarly among the individual families (Fig. 3). As mentioned above, a striking feature is the presence of a very high number of the tricarboxylate-specific TctC family members, namely, 79 for B. pertussis Tohama I and 100 for *C. testosteroni* CNB-2.

Another significant example is the four pseudomonads included in this analysis, namely, the human pathogen Pseudomonas aeruginosa PAO1, the plant pathogen P. syringae pv. tomato DC3000, and the soil bacteria P. fluorescens Pf0-1 and P. putida KT2440. These strains possess very similar SBP numbers (84 to 95), and in all strains, the amino acid-specific family PF0497 (SBP_bac_3) is the most populated family. Further examples of similar SBP profiles of phylogenetically close bacteria are the soil bacterium S. coelicolor A3(2) and the human pathogen N. brasiliensis ATCC 700358, which possess 93 and 80 SBPs, respectively, and which both recognize sugars as the primary ligand (Fig. 3). Furthermore, the soil bacterium B. cepacia 383 and the plant pathogen R. solanacearum GMI1000, which are very close phylogenetically (Fig. 3), possess similar SBP/total-ORF ratios and show a preference for amino acids.

## DISCUSSION

SBPs play a major role in the delivery of organic and inorganic molecules to ABC, TRAP, and TTT transport systems, initiating transport across the membrane. These transport systems are substrate specific, and the initial sequence-based classification of SBPs did not show a correlation with their cognate ligands (34). We show here that SBPs form a large protein superfamily that is composed of proteins recognized by 45 different Pfam signatures. Members of this superfamily differ in size (20 to 65 kDa) and sequence but share the same topology, which consists of two structural lobes connected by a hinge (1). The increase in 3D structural information on SBP-ligand complexes has permitted the classification of SBPs into clusters and subclusters, which are characterized by a well-defined ligand specificity (5). The fact that the ligand profile is reflected in SBP 3D structure is also consistent with the precision of sequence-based prediction of SBP ligands by TransportDB (27, 29).

Previous studies revealed a link between genome size, the number of ABC transport systems, and bacterial lifestyle (35). Furthermore, a relation between transport systems and the bacterial habitat was observed, showing that TRAP systems are more frequent in deep-sea and saline environments (36). However, we did not observe any obvious correlation of the SBP profile with either lifestyle (Fig. 3 and 6) or genome size (Fig. 4). In contrast, there was evidence for phylogenetics-related similarities in the SBP repertoire. This study may form the basis for studies aimed at relating the SBP repertoire with the detailed metabolic maps of the individual strains that may provide insight into the reasons for the differences observed.

To the best of our knowledge, this is the first study to establish and compare the SBP repertoires of different strains. We show that there are very large differences in SBP numbers and types, as well as the predicted ligands. This study thus forms the basis to establish the SBP repertoires of other strains and to tackle central questions on this important superfamily, such as their role in stimulating different transmembrane receptors, thereby coordinating transport with signal transduction. Another central yet poorly understood question is the enormous apparent redundancy of SBPs, which is best illustrated by the 100 TctC family members of *C. testosteroni* CNB-2 that are predicted to be specific for tricarboxylates (Fig. 3). Further studies will show to what degree this redundancy of genes corresponds in fact to a redundancy of proteins with the same ligand spectrum. TctC members bind tricarboxylic acids, and future studies are required to identify the ligands identified by these proteins in order to assess the functional overlap between family members and to determine the proteins that indeed respond to the same ligands.

By far the most populated ligand category was that of SBPs that bind amino acids or peptides, indicating the central importance of amino acid sensing and uptake (Fig. 2B). In this respect, clear parallels to amino acid chemotaxis exist. Currently available data indicate that amino acid-sensing chemoreceptors represent the most abundant functional chemoreceptor family by far (37). Chemotaxis to amino acids facilitates their uptake, which may have driven the coevolution of mechanisms for amino acid chemotaxis and uptake. The importance of amino acid sensing is furthermore demonstrated by a recent study that identified an amino acid-specific subfamily of the dCache sensor domains that is present throughout the tree of life (38). This amino acid-specific domain is abundantly present not only in chemotaxis chemoreceptors but also in all major families of bacterial transmembrane signal transduction receptors, including sensor histidine kinases, diguanylate cyclases and phosphodiesterases, guanylate/adenylate cyclases, serine/threonine kinases and phosphatases, and phosphohydrolases (38). The presence of amino acid-specific sensor domains at these receptors underlies the role of amino acids not only as nutrients but also as central signal molecules that play key roles in regulating multiple processes such as gene expression or the control of second messenger levels, as recently exemplified in P. syringae pv. tomato DC3000 (39), one of the model strains in this work. Several studies have reported the activation of chemoreceptors by the binding of amino acid-loaded SBPs (40–42).

Clear links emerge between the SBP repertoire of a given strain and the information available on the corresponding chemotaxis chemoreceptors. As mentioned above, *C. testosteroni* CNB-2 was predicted to possess 100 SBPs of the tricarboxylate-specific TctC family, representing 2.1% of the total ORFs of this strain (Fig. 3). These SBPs feed substrates into the 66 Tct (tricarboxylate transport) and 9 TRAP transporters for carboxylic acid uptake (43) that were identified in this strain (33). These compounds are then metabolized by the complete tricarboxylic acid (TCA) cycle identified in this strain (33). However, CNB-2 is among the strains for which information on the function of chemotaxis chemoreceptors is available. Of its 20 chemoreceptors (11), so far three have been annotated with a function. Interestingly, all three receptors have been found to bind different tricarboxylic acids: MCP2201 bound exclusively 7 different TCA cycle intermediates (44), and MCP2983 was identified as a *cis*-aconitate specific receptor (45), whereas MCP2901 had a broader ligand range but has a preference for citrate (46). Another example of a link between the SBP and chemoreceptor repertoire is that of P. syringae pv. tomato DC3000, P. fluorescens PF0-1, and P. aeruginosa PAO1, for which amino acids were identified as the principal SBP ligand (Fig. 3). All three strains were found to possess three different amino acid-sensing chemoreceptors, namely, PctA, PctB, and PctC for P. aeruginosa (47, 48), CtaA, CtaB, and CtaC for P. fluorescens (49, 50), and PscA, PscB, and PscC for P. syringae (39, 51). Although these chemoreceptors are homologous and likely to be paralogous within a strain (48), their detailed amino acid ligand profiles differ.

Currently, 13 of the 22 SBP protein families identified in the 49 model bacteria analyzed in this work have been found to interact with either chemoreceptors, sensor kinases, diguanylate cyclase/c-di-GMP phosphodiesterases, or Ser/Thr kinases (22). Given the relatively low number of characterized signal transduction systems that are stimulated by SBP binding, it is likely that members of further SBP families also carry out a function in signal transduction. B. pertussis Tohama I has an elevated number (79) of members of the tricarboxylic-specific TctC family (Fig. 3). So far, there are only about 10 characterized examples of SBP binding to the periplasmic domain of sensor kinases, causing their activation (22). One of these is the B. pertussis TctC family member BctC (BP3867) (Table S2). BctC forms part of a BctCBA tripartite tricarboxylate transporter that mediates the uptake of citrate. It was shown that the citrate-loaded BctC interacts with the periplasmic sensor domain of the BctE sensor kinase, which in turn increases the expression of the *bctCBA* operon, causing an upregulation of the citrate transporter in the presence of citrate (52, 53). B. pertussis was also shown to possess a fully functional TCA cycle (54), but the reason for the enormous redundancy of TctC family members in *C. testosteroni* and B. pertussis is unknown.

Future work is needed to advance understanding of the evolutionary processes that have resulted in the generation of these largely differing SBP repertoires and to characterize and understand the reasons for the observed SBP redundancy in many strains. Furthermore, the inhibition of ligand binding to SBPs has been shown to be an efficient approach to combat pathogens (36, 55), and in this context, this study may be helpful for the development of approaches to fight pathogens of human, animal, and plant relevance.

## MATERIALS AND METHODS

### Sequence retrieval and curation.

The complete proteomes of the selected 49 bacterial strains were downloaded in compressed xml format from the proteome database of UniProtKB (30). A custom Python 3 script was coded for the full query and integrated analysis of the SBP sequences in these proteomes. For this purpose, protein sequences were retrieved when matching one of the following Pfam (26) signatures: PF00405, PF00496, PF00497, PF00532, PF00800, PF01094, PF01297, PF01379, PF01497, PF01547, PF01634, PF02030, PF02470, PF02608, PF02621, PF03180, PF03401, PF03466, PF03480, PF04069, PF04348, PF04392, PF05048, PF05494, PF06646, PF09084, PF09822, PF10613, PF12683, PF12727, PF12849, PF12916, PF12974, PF13343, PF13377, PF13379, PF13407, PF13416, PF13433, PF13458, PF13531, PF13531, PF14503, PF16868, and PF18610. This analysis generated a sequence list for each of the proteomes in SeqRecord format. This format allowed us to store for each of the sequence entries all information that is included in the UniProtKB database. Domains of the above families also serve as sensor domains in cytosolic and transmembrane signal transduction proteins and are thus not SBPs. To identify the SBPs among the initial sequence set, the proportion of the SBP domain with respect to the entire protein was determined, as well as the presence of transmembrane regions and signal peptides. Sequences for which the protein segment covered by the SBP domain profile corresponds to less than 60% of the total protein, which were predicted to have transmembrane regions, or which were not predicted to possess a signal peptide were manually curated. The value of 60% was determined empirically by comparing false-positive and false-negative results using a number of cutoff values. At the value of 60%, an optimal ratio of false-positive and -negative results was obtained. About 10% of automatically retrieved proteins contained a transmembrane region, and around 1% were predicted have a cytosolic location, whereas for about 12%, the SBP Pfam signature covered less than 60% of the total proteins, and the SBP domains are thus likely to be sensor domains of receptors. Manual curation was based on literature searches to retrieve information on protein function, generation of 3D models, prediction of transmembrane regions and signal peptides using Phyre2 (56), Phobius (57), and SignalIP (58), and assessment of the length of the protein segment recognized by the Pfam signature by JPred (59) and Phyre2 (56). Only the Pfam families that were present in at least a single sequence from these 49 proteomes were retained for further analyses.

### Data analysis.

The primary lifestyle/ecological niche was assigned to each of the strains. This was done by analyzing the scientific literature and considering the information on the isolation site as specified in the BioProject section of NCBI (60) and the Integrated Microbial Genomes and Microbiomes (IMG) database (61). A detailed analysis of the lifestyle/ecological niche for each of the strains is provided in Table S1. Subsequently, the ligands that were predicted to bind to the set of SBPs were retrieved from TransportDB (27). For SBPs from strains that were not included in TransportDB (namely, Staphylococcus aureus NCTC 8325 and Escherichia coli MG1655), a BLAST sequence search (62) was performed between the unannotated SBPs and the set of ligand-annotated SBPs. The ligand predicted for the closest homologue (lowest E value) was retained. Ligand and lifestyle annotations were stored in a file harboring the SBP set of the individual strains. Subsequently, predicted ligands were classified into groups following the ChEBI ontology (63) to generate more populated families for subsequent analyses.

In a second stage, the set of SBP sequences for the 49 strains was analyzed and organized according to (i) the number of sequences encoded per genome that match the above-mentioned Pfam families, the number of SBPs, and the ratio of SBPs relative to the total number of proteins per proteome; (ii) the number of SBP family members for each of the strains; (iii) the number of sequences that recognize ligands from each ligand category in each strain’s SBP set and the ratio over all SBP sequences in that strain; and (iv) the number of SBP families and the number of SBPs that recognize determined ligands in each lifestyle category. Finally, the script generated a list of all SBPs in each of the 49 strains, their sequences in FASTA format, and a list of all sequences belonging to each SBP protein family.

### Multiple sequence alignments and construction of phylogenetic trees.

The full list of sequences corresponding to SBPs of the selected 49 strains was subjected to a phylogenetic analysis using the TREND pipeline (64). We selected MAFFT (65) for the alignment of all sequences, with an L-INS-I algorithm, and FastTree (66) was then used for the phylogenetic tree generation (JTT+CAT substitution model and the Shimodaira-Hasegawa test for phylogeny). Five hundred pseudoreplicates were generated for the bootstrap confidence level calculation. The phylogenetic tree was subsequently analyzed, and sequences were annotated with their corresponding SBP Pfam family, colored accordingly, and represented by the iTOL graphical tool (67). Outlier sequences where manually analyzed, and their homology against HMM profiles of the different Pfam families was measured. The taxonomic tree of all strains used in this work was generated with the PhyloT (68) tool by the NCBI taxonomy facility (69). We used the strains’ NCBI TaxIDs assigned for each of the UniProtKB proteomes and represented the tree using the iTOL graphical tool. The pie chart representations of ligand and Pfam family distributions were added by the iTOL editor.

## References

[1] Scheepers GH, Lycklama ANJA, Poolman B. 2016. An updated structural classification of substrate-binding proteins. FEBS Lett 590:4393–4401. doi:10.1002/1873-3468.12445. 27714801

[2] Chu BC, Vogel HJ. 2011. A structural and functional analysis of type III periplasmic and substrate binding proteins: their role in bacterial siderophore and heme transport. Biol Chem 392:39–52. doi:10.1515/BC.2011.012. 21194366

[3] El-Gebali S, Mistry J, Bateman A, Eddy SR, Luciani A, Potter SC, Qureshi M, Richardson LJ, Salazar GA, Smart A, Sonnhammer ELL, Hirsh L, Paladin L, Piovesan D, Tosatto SCE, Finn RD. 2019. The Pfam protein families database in 2019. Nucleic Acids Res 47:D427–D432. doi:10.1093/nar/gky995. 30357350 PMC6324024

[4] Berntsson RP, Smits SH, Schmitt L, Slotboom DJ, Poolman B. 2010. A structural classification of substrate-binding proteins. FEBS Lett 584:2606–2617. doi:10.1016/j.febslet.2010.04.043. 20412802

[5] Fukamizo T, Kitaoku Y, Suginta W. 2019. Periplasmic solute-binding proteins: structure classification and chitooligosaccharide recognition. Int J Biol Macromol 128:985–993. doi:10.1016/j.ijbiomac.2019.02.064. 30771387

[6] Dwyer MA, Hellinga HW. 2004. Periplasmic binding proteins: a versatile superfamily for protein engineering. Curr Opin Struct Biol 14:495–504. doi:10.1016/j.sbi.2004.07.004. 15313245

[7] Vetting MW, Al-Obaidi N, Zhao S, San Francisco B, Kim J, Wichelecki DJ, Bouvier JT, Solbiati JO, Vu H, Zhang X, Rodionov DA, Love JD, Hillerich BS, Seidel RD, Quinn RJ, Osterman AL, Cronan JE, Jacobson MP, Gerlt JA, Almo SC. 2015. Experimental strategies for functional annotation and metabolism discovery: targeted screening of solute binding proteins and unbiased panning of metabolomes. Biochemistry 54:909–931. doi:10.1021/bi501388y. 25540822 PMC4310620

[8] Li L, Ghimire-Rijal S, Lucas SL, Stanley CB, Wright E, Agarwal PK, Myles DA, Cuneo MJ. 2017. Periplasmic binding protein dimer has a second allosteric event tied to ligand binding. Biochemistry 56:5328–5337. doi:10.1021/acs.biochem.7b00657. 28876049

[9] Song L, Zhang Y, Chen W, Gu T, Zhang SY, Ji Q. 2018. Mechanistic insights into staphylopine-mediated metal acquisition. Proc Natl Acad Sci USA 115:3942–3947. doi:10.1073/pnas.1718382115. 29581261 PMC5899449

[10] Galperin MY. 2018. What bacteria want. Environ Microbiol 20:4221–4229. doi:10.1111/1462-2920.14398. 30187651 PMC7020242

[11] Gumerov VM, Ortega DR, Adebali O, Ulrich LE, Zhulin IB. 2020. MiST 3.0: an updated microbial signal transduction database with an emphasis on chemosensory systems. Nucleic Acids Res 48:D459–D464. doi:10.1093/nar/gkz988. 31754718 PMC6943060

[12] Ortega A, Zhulin IB, Krell T. 2017. Sensory repertoire of bacterial chemoreceptors. Microbiol Mol Biol Rev 81:e00033-17. doi:10.1128/MMBR.00033-17. PMC5706747 29070658

[13] Upadhyay AA, Fleetwood AD, Adebali O, Finn RD, Zhulin IB. 2016. Cache domains that are homologous to, but different from PAS domains comprise the largest superfamily of extracellular sensors in prokaryotes. PLoS Comput Biol 12:e1004862. doi:10.1371/journal.pcbi.1004862. 27049771 PMC4822843

[14] Manson MD, Blank V, Brade G, Higgins CF. 1986. Peptide chemotaxis in *E. coli* involves the Tap signal transducer and the dipeptide permease. Nature 321:253–256. doi:10.1038/321253a0. 3520334

[15] Rico-Jimenez M, Reyes-Darias JA, Ortega A, Diez Pena AI, Morel B, Krell T. 2016. Two different mechanisms mediate chemotaxis to inorganic phosphate in *Pseudomonas aeruginosa*. Sci Rep 6:28967. doi:10.1038/srep28967. 27353565 PMC4926252

[16] Li J, Wang C, Yang G, Sun Z, Guo H, Shao K, Gu Y, Jiang W, Zhang P. 2017. Molecular mechanism of environmental d-xylose perception by a XylFII-LytS complex in bacteria. Proc Natl Acad Sci USA 114:8235–8240. doi:10.1073/pnas.1620183114. 28716923 PMC5547591

[17] Moore JO, Hendrickson WA. 2012. An asymmetry-to-symmetry switch in signal transmission by the histidine kinase receptor for TMAO. Structure 20:729–741. doi:10.1016/j.str.2012.02.021. 22483119 PMC3625974

[18] Trimble MJ, McCarter LL. 2011. Bis-(3'-5')-cyclic dimeric GMP-linked quorum sensing controls swarming in *Vibrio parahaemolyticus*. Proc Natl Acad Sci USA 108:18079–18084. doi:10.1073/pnas.1113790108. 22006340 PMC3207653

[19] Sobe RC, Bond WG, Wotanis CK, Zayner JP, Burriss MA, Fernandez N, Bruger EL, Waters CM, Neufeld HS, Karatan E. 2017. Spermine inhibits *Vibrio cholerae* biofilm formation through the NspS-MbaA polyamine signaling system. J Biol Chem 292:17025–17036. doi:10.1074/jbc.M117.801068. 28827313 PMC5641875

[20] Bhattacharyya N, Nkumama IN, Newland-Smith Z, Lin L-Y, Yin W, Cullen RE, Griffiths JS, Jarvis AR, Price MJ, Chong PY, Wallis R, O’Hare HM. 2018. An aspartate-specific solute-binding protein regulates protein kinase G activity to control glutamate metabolism in *Mycobacteria*. mBio 9:e00931-18. doi:10.1128/mBio.00931-18. 30065086 PMC6069109

[21] Rieck B, Degiacomi G, Zimmermann M, Cascioferro A, Boldrin F, Lazar-Adler NR, Bottrill AR, le Chevalier F, Frigui W, Bellinzoni M, Lisa MN, Alzari PM, Nguyen L, Brosch R, Sauer U, Manganelli R, O'Hare HM. 2017. PknG senses amino acid availability to control metabolism and virulence of *Mycobacterium tuberculosis*. PLoS Pathog 13:e1006399. doi:10.1371/journal.ppat.1006399. 28545104 PMC5448819

[22] Matilla MA, Ortega A, Krell T. 2021. The role of solute binding proteins in signal transduction. Comput Struct Biotechnol J 19:1786–1805. doi:10.1016/j.csbj.2021.03.029. 33897981 PMC8050422

[23] Zhang Y, Gardina PJ, Kuebler AS, Kang HS, Christopher JA, Manson MD. 1999. Model of maltose-binding protein/chemoreceptor complex supports intrasubunit signaling mechanism. Proc Natl Acad Sci USA 96:939–944. doi:10.1073/pnas.96.3.939. 9927672 PMC15329

[24] Kondoh H, Ball CB, Adler J. 1979. Identification of a methyl-accepting chemotaxis protein for the ribose and galactose chemoreceptors of *Escherichia coli*. Proc Natl Acad Sci USA 76:260–264. doi:10.1073/pnas.76.1.260. 370826 PMC382918

[25] Hegde M, Englert DL, Schrock S, Cohn WB, Vogt C, Wood TK, Manson MD, Jayaraman A. 2011. Chemotaxis to the quorum-sensing signal AI-2 requires the Tsr chemoreceptor and the periplasmic LsrB AI-2-binding protein. J Bacteriol 193:768–773. doi:10.1128/JB.01196-10. 21097621 PMC3021223

[26] Mistry J, Chuguransky S, Williams L, Qureshi M, Salazar GA, Sonnhammer ELL, Tosatto SCE, Paladin L, Raj S, Richardson LJ, Finn RD, Bateman A. 2021. Pfam: the protein families database in 2021. Nucleic Acids Res 49:D412–D419. doi:10.1093/nar/gkaa913. 33125078 PMC7779014

[27] Elbourne LD, Tetu SG, Hassan KA, Paulsen IT. 2017. TransportDB 2.0: a database for exploring membrane transporters in sequenced genomes from all domains of life. Nucleic Acids Res 45:D320–D324. doi:10.1093/nar/gkw1068. 27899676 PMC5210551

[28] O'Hara BP, Norman RA, Wan PT, Roe SM, Barrett TE, Drew RE, Pearl LH. 1999. Crystal structure and induction mechanism of AmiC-AmiR: a ligand-regulated transcription antitermination complex. EMBO J 18:5175–5186. doi:10.1093/emboj/18.19.5175. 10508151 PMC1171588

[29] Fernandez M, Rico-Jimenez M, Ortega A, Daddaoua A, Garcia Garcia AI, Martin-Mora D, Torres NM, Tajuelo A, Matilla MA, Krell T. 2019. Determination of ligand profiles for *Pseudomonas aeruginosa* solute binding proteins. Int J Mol Sci 20:5156. doi:10.3390/ijms20205156. 31627455 PMC6829864

[30] UniProt Consortium. 2021. UniProt: the universal protein knowledgebase in 2021. Nucleic Acids Res 49:D480–D489. doi:10.1093/nar/gkaa1100. 33237286 PMC7778908

[31] Rendulic S, Jagtap P, Rosinus A, Eppinger M, Baar C, Lanz C, Keller H, Lambert C, Evans KJ, Goesmann A, Meyer F, Sockett RE, Schuster SC. 2004. A predator unmasked: life cycle of *Bdellovibrio bacteriovorus* from a genomic perspective. Science 303:689–692. doi:10.1126/science.1093027. 14752164

[32] Parkhill J, Sebaihia M, Preston A, Murphy LD, Thomson N, Harris DE, Holden MTG, Churcher CM, Bentley SD, Mungall KL, Cerdeño-Tárraga AM, Temple L, James K, Harris B, Quail MA, Achtman M, Atkin R, Baker S, Basham D, Bason N, Cherevach I, Chillingworth T, Collins M, Cronin A, Davis P, Doggett J, Feltwell T, Goble A, Hamlin N, Hauser H, Holroyd S, Jagels K, Leather S, Moule S, Norberczak H, O'Neil S, Ormond D, Price C, Rabbinowitsch E, Rutter S, Sanders M, Saunders D, Seeger K, Sharp S, Simmonds M, Skelton J, Squares R, Squares S, Stevens K, Unwin L, et al. 2003. Comparative analysis of the genome sequences of *Bordetella pertussis, Bordetella parapertussis* and *Bordetella bronchiseptica*. Nat Genet 35:32–40. doi:10.1038/ng1227. 12910271

[33] Ma YF, Zhang Y, Zhang JY, Chen DW, Zhu Y, Zheng H, Wang SY, Jiang CY, Zhao GP, Liu SJ. 2009. The complete genome of *Comamonas testosteroni* reveals its genetic adaptations to changing environments. Appl Environ Microbiol 75:6812–6819. doi:10.1128/AEM.00933-09. 19734336 PMC2772449

[34] Tam R, Saier MH, Jr. 1993. Structural, functional, and evolutionary relationships among extracellular solute-binding receptors of bacteria. Microbiol Rev 57:320–346. doi:10.1128/mr.57.2.320-346.1993. 8336670 PMC372912

[35] Davidson AL, Dassa E, Orelle C, Chen J. 2008. Structure, function, and evolution of bacterial ATP-binding cassette systems. Microbiol Mol Biol Rev 72:317–364. doi:10.1128/MMBR.00031-07. 18535149 PMC2415747

[36] Davies JS, Currie MJ, Wright JD, Newton-Vesty MC, North RA, Mace PD, Allison JR, Dobson RCJ. 2021. Selective nutrient transport in bacteria: multicomponent transporter systems reign supreme. Front Mol Biosci 8:699222. doi:10.3389/fmolb.2021.699222. 34268334 PMC8276074

[37] Matilla MA, Velando F, Martin-Mora D, Monteagudo-Cascales E, Krell T. 2022. A catalogue of signal molecules that interact with sensor kinases, chemoreceptors and transcriptional regulators. FEMS Microbiol Rev 46:fuab043. doi:10.1093/femsre/fuab043. 34424339

[38] Gumerov VM, Andrianova EP, Matilla MA, Page KM, Monteagudo-Cascales E, Dolphin AC, Krell T, Zhulin IB. 2022. Amino acid sensor conserved from bacteria to humans. Proc Natl Acad Sci USA 119:e2110415119. doi:10.1073/pnas.2110415119. 35238638 PMC8915833

[39] Cerna-Vargas JP, Santamaria-Hernando S, Matilla MA, Rodriguez-Herva JJ, Daddaoua A, Rodriguez-Palenzuela P, Krell T, Lopez-Solanilla E. 2019. Chemoperception of specific amino acids controls phytopathogenicity in *Pseudomonas syringae* pv. tomato. mBio 10:e01868-19. doi:10.1128/mBio.01868-19. 31575767 PMC6775455

[40] Glekas GD, Mulhern BJ, Kroc A, Duelfer KA, Lei V, Rao CV, Ordal GW. 2012. The *Bacillus subtilis* chemoreceptor McpC senses multiple ligands using two discrete mechanisms. J Biol Chem 287:39412–39418. doi:10.1074/jbc.M112.413518. 23038252 PMC3501012

[41] Kokoeva MV, Oesterhelt D. 2000. BasT, a membrane-bound transducer protein for amino acid detection in *Halobacterium salinarum*. Mol Microbiol 35:647–656. doi:10.1046/j.1365-2958.2000.01735.x. 10672186

[42] Kokoeva MV, Storch KF, Klein C, Oesterhelt D. 2002. A novel mode of sensory transduction in archaea: binding protein-mediated chemotaxis towards osmoprotectants and amino acids. EMBO J 21:2312–2322. doi:10.1093/emboj/21.10.2312. 12006484 PMC125379

[43] Winnen B, Hvorup RN, Saier MH, Jr. 2003. The tripartite tricarboxylate transporter (TTT) family. Res Microbiol 154:457–465. doi:10.1016/S0923-2508(03)00126-8. 14499931

[44] Ni B, Huang Z, Fan Z, Jiang CY, Liu SJ. 2013. *Comamonas testosteroni* uses a chemoreceptor for tricarboxylic acid cycle intermediates to trigger chemotactic responses towards aromatic compounds. Mol Microbiol 90:813–823. doi:10.1111/mmi.12400. 24102855

[45] Ni B, Huang Z, Wu YF, Fan Z, Jiang CY, Liu SJ. 2015. A novel chemoreceptor MCP2983 from *Comamonas testosteroni* specifically binds to *cis*-aconitate and triggers chemotaxis towards diverse organic compounds. Appl Microbiol Biotechnol 99:2773–2781. doi:10.1007/s00253-014-6216-3. 25511821

[46] Huang Z, Ni B, Jiang CY, Wu YF, He YZ, Parales RE, Liu SJ. 2016. Direct sensing and signal transduction during bacterial chemotaxis toward aromatic compounds in *Comamonas testosteroni*. Mol Microbiol 101:224–237. doi:10.1111/mmi.13385. 27008921

[47] Taguchi K, Fukutomi H, Kuroda A, Kato J, Ohtake H. 1997. Genetic identification of chemotactic transducers for amino acids in *Pseudomonas aeruginosa*. Microbiology 143:3223–3229. doi:10.1099/00221287-143-10-3223. 9353923

[48] Gavira JA, Gumerov VM, Rico-Jimenez M, Petukh M, Upadhyay AA, Ortega A, Matilla MA, Zhulin IB, Krell T. 2020. How bacterial chemoreceptors evolve novel ligand specificities. mBio 11:e03066-19. doi:10.1128/mBio.03066-19. 31964737 PMC6974571

[49] Oku S, Komatsu A, Tajima T, Nakashimada Y, Kato J. 2012. Identification of chemotaxis sensory proteins for amino acids in *Pseudomonas fluorescens* Pf0-1 and their involvement in chemotaxis to tomato root exudate and root colonization. Microbes Environ 27:462–469. doi:10.1264/jsme2.me12005. 22972385 PMC4103555

[50] Ud-Din A, Khan MF, Roujeinikova A. 2020. Broad specificity of amino acid chemoreceptor CtaA of *Pseudomonas fluorescens* is afforded by plasticity of its amphipathic ligand-binding pocket. Mol Plant Microbe Interact 33:612–623. doi:10.1094/MPMI-10-19-0277-R. 31909676

[51] McKellar JL, Minnell JJ, Gerth ML. 2015. A high-throughput screen for ligand binding reveals the specificities of three amino acid chemoreceptors from *Pseudomonas syringae* pv. *actinidiae*. Mol Microbiol 96:694–707. doi:10.1111/mmi.12964. 25656450

[52] Antoine R, Huvent I, Chemlal K, Deray I, Raze D, Locht C, Jacob-Dubuisson F. 2005. The periplasmic binding protein of a tripartite tricarboxylate transporter is involved in signal transduction. J Mol Biol 351:799–809. doi:10.1016/j.jmb.2005.05.071. 16045930

[53] Antoine R, Jacob-Dubuisson F, Drobecq H, Willery E, Lesjean S, Locht C. 2003. Overrepresentation of a gene family encoding extracytoplasmic solute receptors in *Bordetella*. J Bacteriol 185:1470–1474. doi:10.1128/JB.185.4.1470-1474.2003. 12562821 PMC142875

[54] Izac M, Garnier D, Speck D, Lindley ND. 2015. A functional tricarboxylic acid cycle operates during growth of *Bordetella pertussis* on amino acid mixtures as sole carbon substrates. PLoS One 10:e0145251. doi:10.1371/journal.pone.0145251. 26684737 PMC4684311

[55] Ilari A, Pescatori L, Di Santo R, Battistoni A, Ammendola S, Falconi M, Berlutti F, Valenti P, Chiancone E. 2016. *Salmonella enterica* serovar Typhimurium growth is inhibited by the concomitant binding of Zn(II) and a pyrrolyl-hydroxamate to ZnuA, the soluble component of the ZnuABC transporter. Biochim Biophys Acta 1860:534–541. doi:10.1016/j.bbagen.2015.12.006. 26691136

[56] Kelley LA, Mezulis S, Yates CM, Wass MN, Sternberg MJ. 2015. The Phyre2 web portal for protein modeling, prediction and analysis. Nat Protoc 10:845–858. doi:10.1038/nprot.2015.053. 25950237 PMC5298202

[57] Kall L, Krogh A, Sonnhammer EL. 2004. A combined transmembrane and signal peptide predcition method. J Mol Biol 338:1027–1036. doi:10.1016/j.jmb.2004.03.016. 15111065

[58] Almagro Armenteros JJ, Tsirigos KD, Sønderby CK, Petersen TN, Winther O, Brunak S, von Heijne G, Nielsen H. 2019. SignalP 5.0 improves signal peptide predictions using deep neural networks. Nat Biotechnol 37:420–423. doi:10.1038/s41587-019-0036-z. 30778233

[59] Drozdetskiy A, Cole C, Procter J, Barton GJ. 2015. JPred4: a protein secondary structure prediction server. Nucleic Acids Res 43:W389–W394. doi:10.1093/nar/gkv332. 25883141 PMC4489285

[60] Sayers EW, Bolton EE, Brister JR, Canese K, Chan J, Comeau DC, Connor R, Funk K, Kelly C, Kim S, Madej T, Marchler-Bauer A, Lanczycki C, Lathrop S, Lu Z, Thibaud-Nissen F, Murphy T, Phan L, Skripchenko Y, Tse T, Wang J, Williams R, Trawick BW, Pruitt KD, Sherry ST. 2022. Database resources of the National Center for Biotechnology Information. Nucleic Acids Res 50:D20–D26. doi:10.1093/nar/gkab1112. 34850941 PMC8728269

[61] Chen IA, Chu K, Palaniappan K, Ratner A, Huang J, Huntemann M, Hajek P, Ritter S, Varghese N, Seshadri R, Roux S, Woyke T, Eloe-Fadrosh EA, Ivanova NN, Kyrpides NC. 2021. The IMG/M data management and analysis system v.6.0: new tools and advanced capabilities. Nucleic Acids Res 49:D751–D763. doi:10.1093/nar/gkaa939. 33119741 PMC7778900

[62] Altschul SF, Gish W, Miller W, Myers EW, Lipman DJ. 1990. Basic local alignment search tool. J Mol Biol 215:403–410. doi:10.1016/S0022-2836(05)80360-2. 2231712

[63] Hastings J, Owen G, Dekker A, Ennis M, Kale N, Muthukrishnan V, Turner S, Swainston N, Mendes P, Steinbeck C. 2016. ChEBI in 2016: improved services and an expanding collection of metabolites. Nucleic Acids Res 44:D1214–D1219. doi:10.1093/nar/gkv1031. 26467479 PMC4702775

[64] Gumerov VM, Zhulin IB. 2020. TREND: a platform for exploring protein function in prokaryotes based on phylogenetic, domain architecture and gene neighborhood analyses. Nucleic Acids Res 48:W72–W76. doi:10.1093/nar/gkaa243. 32282909 PMC7319448

[65] Katoh K, Rozewicki J, Yamada KD. 2019. MAFFT online service: multiple sequence alignment, interactive sequence choice and visualization. Brief Bioinform 20:1160–1166. doi:10.1093/bib/bbx108. 28968734 PMC6781576

[66] Price MN, Dehal PS, Arkin AP. 2010. FastTree 2–approximately maximum-likelihood trees for large alignments. PLoS One 5:e9490. doi:10.1371/journal.pone.0009490. 20224823 PMC2835736

[67] Letunic I, Bork P. 2021. Interactive Tree Of Life (iTOL) v5: an online tool for phylogenetic tree display and annotation. Nucleic Acids Res 49:W293–W296. doi:10.1093/nar/gkab301. 33885785 PMC8265157

[68] Letunic I. 2015. phyloT: Phylogenetic Tree Generator. http://phylot.biobyte.de/.

[69] Schoch CL, Ciufo S, Domrachev M, Hotton CL, Kannan S, Khovanskaya R, Leipe D, McVeigh R, O'Neill K, Robbertse B, Sharma S, Soussov V, Sullivan JP, Sun L, Turner S, Karsch-Mizrachi I. 2020. NCBI Taxonomy: a comprehensive update on curation, resources and tools. Database (Oxford) 2020:baaa062. doi:10.1093/database/baaa062. 32761142 PMC7408187

