# Supplementary material for: The Repertoire of Solute-Binding Proteins of Model Bacteria Reveals Large Differences in Number, Type, and Ligand Range
Source: Microbiol Spectr. 2022 Sep 19;10(5):e02054-22. doi: 10.1128/spectrum.02054-22 (PMC9602780; doi:10.1128/spectrum.02054-22)
Supplement: Supplemental file 1 — Download spectrum.02054-22-s0001.pdf, PDF file, 0.6 MB [file spectrum.02054-22-s0001.pdf]

Supplemental Material

to

The repertoire of solute binding proteins of model bacteria reveals large differences in number, type and ligand range

by

Álvaro Ortega, Miguel A. Matilla and Tino Krell

Figure S1) Flowchart of the individual steps leading to the establishment of the SBP repertoire of the 49 bacterial model strains used in this work.

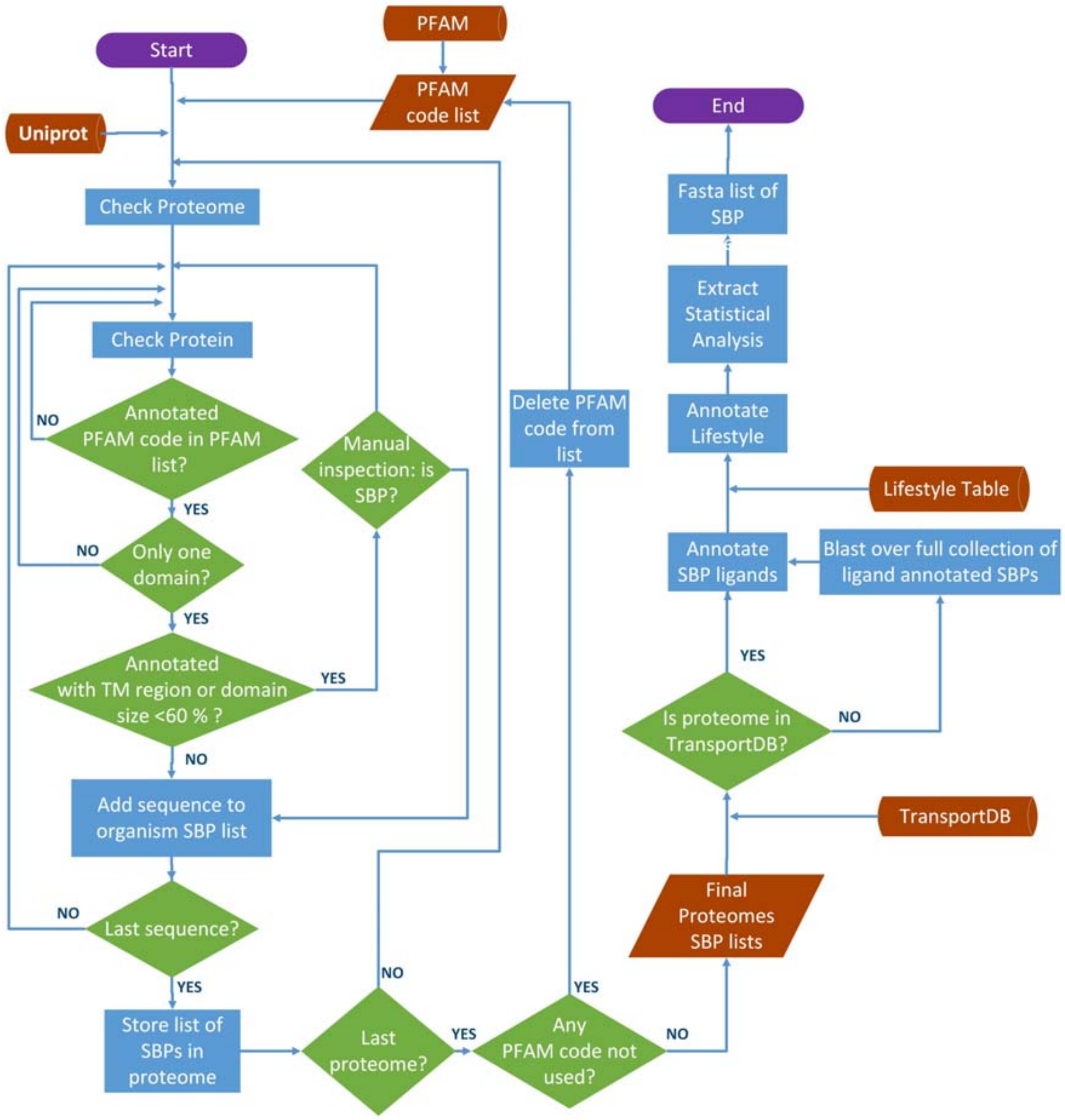

Table S1) Assignation of a lifestyle to the different bacterial strains.

Strain	Lifestyle/Isolation source	Comments	Ref.
Thermotoga maritima MSB8	Sediments (marine/fresh water)	Isolated from a geothermal heated marine sediment (anaerobic marine mud) at Porto di Levante, Vulcano, Italy. Free living, non-spore-forming, rod-shaped bacterium. Grows optimally at 80 ºC. Metabolizes many simple and complex carbohydrates including glucose, sucrose, starch, cellulose and xylan. Habitat: Marine, marine sediment, mud.	(1)
Chlamydia trachomatis D/UW-3/CX	Human/animal pathogen	Genitotropic isolate, isolated from the cervix of an asymptomatic female. Opportunistic human pathogen. Causal agent of preventable blindness and sexually transmitted disease (STD) in humans.	(2)
Borrelia burgdorferi B31	Human/animal pathogen	Isolated from ticks, *Ixodes dammini*, in New York in 1981. Human and animal pathogen, aetiologic agent of Lyme disease.	(3)
Spirochaeta thermophila Z-1203 (=DSM 6578)	Fresh/marine waters	Isolated from a marine hot spring in Shiashkoten island (Russia). Free living, non-pathogenic. Habitat: Aquatic, hot spring, marine.	[a]
Synechocystis sp. PCC 6803	Fresh/marine waters	Isolated from freshwater lake in 1968. Photosynthetic cyanobaceria, non-pathogenic.	(4)
Anabaena cylindrica PCC 7122	Fresh/marine waters	Isolated from water, most likely pond, in Cambridge (UK) in 1939. Photosynthetic bacterium. Free living, non-pathogenic.	[b]
Streptomyces coelicolor A3(2)	Soil	Saprophytic soil-dwelling, filamentous bacteria. Antibiotic producer. Free-living.	(5)
Nocardia brasiliensis ATCC 700358	Human/animal pathogen	Isolated from human mycetoma in Monterrey (Mexico) in 1989. Largely found in *in vitro* and experimental models of actinomycetoma. Soil	(6)

		inhabitant actinobacterium. Human pathogen.	
Mycobacterium tuberculosis H37Rv	Human/animal pathogen	Isolated from a human lung in 1905 and passaged outside of the human host. Human and animal pathogen. Susceptible to drugs and amenable to genetic manipulation. Causal agent of tuberculosis.	(7)
Clostridium botulinum A str. ATCC 3502	Human/animal pathogen	Isolated from a case of foodborne botulism associated with canned peas in California in the early 1920s. Causative agent of botulism in humans and animals. Can live in soils.	(8)
Lactobacillus acidophilus NCFM	Human intestinal microflora	First isolated in the 1970s from human feces. Contribute to the organisms' gastric survival and promote interactions with the intestinal mucosa and microbiota. Habitat: Host, intestinal tract, oral cavity, vagina. Probiotic strain. Non-pathogenic.	(9)
Lactococcus lactis subsp. *lactis* Il1403	Food	The strain IL1403 is a plasmid-free derivative of the strain IL594, isolated from a cheese starter culture. Now used as a lab workhorse. Free-living.	(10)
Streptococcus pneumoniae R6	Human/animal pathogen	Avirulent laboratory-adapted *Streptococcus pneumoniae* D39 derivative that lacks polysaccharide capsule. D39 is a clinical isolate from 1916. Human and animal pathogen. Causal agent of pneumococcal disease. Strain R6 is genetically tractable and is used worldwide as a standard laboratory strain.	(11)
Staphylococcus aureus NCTC 8325	Human/animal pathogen	Isolated in 1960 from a sepsis patient. Host associated. Causative agent of staphylococcal scarlet fever and toxic-shock syndrome. Human pathogen.	(12)
Geobacillus thermodenitrificans NG80-2	Oil reservoir	Isolated from a deep-subsurface oil reservoir in Dagang oilfield, Northern China. Non-pathogenic. Habitat: Fresh water, oil fields.	(13)

Bacillus amyloliquefaciens FZB42	Soil	Isolated from a plant-pathogen infested soil and its organic material. Plant root-colonizing bacterium. Stimulates plant growth and produces secondary metabolites that suppress soil-borne plant pathogens.	(14)
Bacillus subtilis subsp. *subtilis* 168	Soil	Radiation induced mutant of the *Bacillus subtilis* Marburg strain in 1947. Highly transformable and tryptophan auxotroph strain. Used worldwide as a standard laboratory strain. Nonpathogenic, aerobic, endospore-forming, rod-shaped bacterium. *Bacillus subtilis* species are commonly found in soils.	(15)
Bdellovibrio bacteriovorus HD100	Soil	Soil isolate. Free living. Predator of other Gram-negative bacteria. Habitat: Fresh water, soil. Bacteriolytic, ectoparasite.	(16)
Helicobacter pylori 26695	Human/animal pathogen	Isolated from a patient in the United Kingdom with gastritis. Human pathogen. Causes gastric inflammation and ulcer.	(17)
Neisseria meningitidis MC58	Human/animal pathogen	Isolated from a patient with meningococcal meningitis in the UK. Human pathogen. Causes meningococcal infection and septicemia.	(18)
Bordetella pertussis Tohama I	Human/animal pathogen	Originally isolated from a patient with whooping cough in Japan in the 1950s, and used as a vaccine strain. Human pathogen. Causal agent of Pertussis - respiratory infection	(19)
Comamonas testosteroni CNB-2	Active sludge	Strain CNB-2 is a mutant of *C. testosteroni* CNB-1 that lost the degrading plasmid pCNB1. Strain CNB-1 was isolated from activated sludge. Capable of mineralizing complex and xenobiotic compounds, such as testosterone and 4-chloronitrobenzene (CNB). Free living.	(20)
Burkholderia cepacia 383	Soil	Isolated from forest soil in Trinidad in 1958. Nitrogen fixer. *Burkholderia cepacia* strains are common opportunistic pathogens that cause lung infections in immunocompromised and cystic fibrosis patients. Habitat: Host, rhizosphere, soil.	[c]

Ralstonia solanacearum GMI1000	Plant pathogen	Isolated from a wilted tomato plant in the French Guyana. Plant pathogen. *Ralstonia solanacearum* species are devastating, soil-borne plant pathogens that are the agents causing bacterial wilt disease in over 200 plant species, important crops like potato, tomato, peanut, eggplant and banana.	(21)
Sphingomonas wittichii RW1	Fresh/marine waters	Isolated from river Elbe (Germany) due to its ability to grow on toxic dioxin pollutants as the sole carbon and energy source.	(22)
Caulobacter crescentus CB15	Fresh/marine waters	Isolated from a pond water in the USA. Free-living non-pathogenic bacterium.	(23)
Rhodobacter sphaeroides ATCC 17025	Fresh/marine waters	Unknown isolation source. Metabolically diverse, nonsufur, denitrifier, phototrophic bacterium. Can detoxify metal oxides. Non-pathogenic. This species is frequently found in soils, in anoxic zones of waters, mud, sludge, and in organic-rich water habitats. Free living.	(24)
Azospirillum baldaniorum Sp245^T (formerly *Azospirillum brasilense* Sp245^T)	Beneficial plant associated	Isolated from a wheat (*Triticum aestivum* L.) root in Brazil. Bacteria of the genus *Azospirillum* are found primarily in terrestrial habitats, where they colonize roots of important cereals and other grasses and promote plant growth by several mechanisms, including its ability to fix atmospheric nitrogen and to produce plant-beneficial compounds. Habitat: Rhizosphere, soil.	(25)
Brucella abortus 2308	Human/animal pathogen	Highly virulent strain recovered in 1940 from an aborted fetus of a cow. The host range is humans, cattle, and certain other domestic animals. Host-associated. Intracellular parasite. Causes spontaneous abortion.	(26)
Sinorhizobium (*Ensifer*) *meliloti* 1021	Beneficial plant associated	Endosymbiont of alfalfa (*Medicago sativa*) and its close relative *Medicago truncatula.* Forms agronomically important N_2-fixing root nodules in legumes. The strain 1021 is a streptomycin resistant derivative of the strain 2011 - a nitrogen-fixing symbiont capable of	(27)

		living either free in the soil or in symbiotic association with roots of legume plants such as the model legume *Medicago truncatula*.	
Agrobacterium fabrum C58	Plant pathogen	Isolated from a cherry tree (*Prunus* sp. Cv. Montmorency) tumor. Plant pathogen. Causal agent of crown gall disease in plants.	(28)
Acinetobacter baumannii AB0057	Human/animal pathogen	Isolated from a patient with bloodstream infection at Walter Reed Army Medical Center (USA). Human pathogen. *Acinetobacter baumannii* is the leading cause of opportunistic nosocomial infections in immunocompromised hosts. This human pathogen is responsible for a vast array of infections, of which ventilator-associated pneumonia and urinary tract and bloodstream infections are the most common.	(29)
Xanthomonas campestris pv. *campestris* ATCC 33913)	Plant pathogen	Isolated from cabbage (*Brassica oleracea* var. *gemmifera*). Plant pathogen. Causes black rot, which affects crucifers such as Brassica and Arabidopsis.	(30)
Legionella pneumophila subsp. *pneumophila* str. Philadelphia-1	Human/animal pathogen	Isolated from a lung specimen of a patient who had died from Legionnaires' disease. Human pathogen. Causal agent of Legionellosis.	(31)
Shewanella oneidensis MR-1	Sediments (marine/fresh water)	Sediment isolate from the Oneida lake in New York (USA). Facultatively aerobic bacterium with diverse respiratory capacities (i.e. reduction of iron and manganese oxides). Model organism for bioremediation studies.	(32)
Aliivibrio fischeri MJ11	Animal symbiont	Isolated from the light organ of the japanese pinecone fish, *Monocentris japonica*, in Japan. Fish symbiont. Host-associated.	(33)
Vibrio cholerae O1 biovar El Tor str. N16961	Human/animal pathogen	Isolated from a stool from a cholera patient in an epidemic outbreak in Bangladesh in 1971. Hemolysin producer. Habitat: Gastrointestinal tract. Human pathogen. Causal agent of cholera disease.	(34)

Azotobacter vinelandii DJ	Soil	A high-frequency transforming variant of *Azotobacter vinelandii* UW generated in 1984 through multiple rounds of transformation. UW is a soil bacterium related to the *Pseudomonas* genus that fixes nitrogen under aerobic conditions, while simultaneously protecting nitrogenase from oxygen damage. Stores polyhydroxybutyrate. Free living.	(35)
Pseudomonas syringae pv. *tomato* DC3000	Plant pathogen	Isolated in 1960 from tomato in the Channel Islands in Guernsey, United Kingdom. Causes bacterial speck disease in plants including tomato and *Arabidopsis thaliana*. Habitat: Plant endophyte, fresh water, soil.	(36)
Pseudomonas fluorescens Pf0-1	Soil	Isolated in 1987 from an agricultural loam soil in Massachusetts (USA). Considered as a bacterium potentially important for biocontrol and bioremediation. Rhizosphere colonizer. Habitat: Soil, terrestrial, rhizosphere. Non-pathogenic.	(37)
Pseudomonas putida KT2440	Soil	Saprophytic soil isolate. Rhizosphere colonizer, plant-growth promoter and biocontrol agent. Plasmid-free derivative of a toluene-degrading bacterium, *P. putida* mt-2. Certified as a biosafety host for the cloning of foreign genes.	(38)
Pseudomonas aeruginosa PAO1	Human/animal pathogen	A spontaneous chloramphenicol-resistant mutant of the original PAO strain (earlier called *P. aeruginosa* strain 1) that had been isolated in 1954 from a wound in Melbourne, Australia. Now a lab workhorse. Intrinsically resistant to antibiotics and disinfectants. Opportunistic human pathogen. Habitat: Fresh water, host, soil, wastewater.	(39)
Pectobacterium atrosepticum SCRI1043	Plant pathogen	Isolated from a potato stem with blackleg disease symptoms in 1985 in Perthshire, Scotland. Plant pathogen. Causative agent of soft rot and blackleg potato diseases.	(40)
Photorhabdus luminescens subsp.	Human/animal pathogen	A symbiont of the nematode *Heterorhabditis bacteriophora* isolated on Trinidad and Tobago. Host associated. Can cause septicemia and	(41)

laumondii TTO1		toxemia. Symbiotic with soil entomopathogenic nematodes and pathogenic to a wide range of insects.	
Yersinia pestis Nepal516	Human/animal pathogen	Isolated from a human infection in Nepal, possibly from a 1967 outbreak of pneumonic plague. Causal agent of bubonic and pneumonic plague. Human pathogen. Habitat: host and soil.	(42)
Serratia plymuthica S13	Beneficial plant associated	Isolated from the anthosphere of styrian oil pumpkin (*Cucurbita pepo* L. subsp. *pepo* var. *styriaca*). Rhizosphere and endosphere colonizer. Biocontrol agent.	(43)
Salmonella typhimurium serovar Typhimurium LT2	Human/animal pathogen	Isolated from a human clinical infection at Stoke Mandeville hospital (UK) in the 1940s. Model strain to studying the genetics of *Salmonella* worldwide. Human and animal pathogen. Leading cause of human gastroenteritis.	(44)
Escherichia coli MG1655	Human intestinal microflora	MG1655 was derived from the strain W1485, which was derived from a stab-culture descendant of the original K-12 isolate. This original *E. coli* strain K-12 was obtained from a stool sample of a diphtheria patient in Palo Alto (California, USA) in 1922. Colonizes the lower gut of humans and animals. Maintained as a laboratory strain with minimal genetic manipulation, having only been cured of the temperate bacteriophage lambda and F plasmid by ultraviolet light and acridine orange, respectively.	(45)
Klebsiella pneumoniae HS11286	Human/animal pathogen	Human sputum isolate from a patient in Shanghai (China). Antibiotic resistant. Human pathogen.	(46)

[a] NC_017583.1

[b] NC_019771.1 (chromosome), NC_019772.1, NC_020157.1, NC_019773.1, NC_019774.1, NC_020056.1, NC_019775.1 (six plasmids)

[c] NC_007509.1,NC_007510.1,NC_007511.1 (three chromosomes)

REFERENCES

1. Latif H, Lerman JA, Portnoy VA, Tarasova Y, Nagarajan H, Schrimpe-Rutledge AC, Smith RD, Adkins JN, Lee D-H, Qiu Y, Zengler K. 2013. The genome organization of Thermotoga maritima reflects its lifestyle. PLoS Genet 9:e1003485.
2. Stephens RS, Kalman S, Lammel C, Fan J, Marathe R, Aravind L, Mitchell W, Olinger L, Tatusov RL, Zhao Q, Koonin EV, Davis RW. 1998. Genome sequence of an obligate intracellular pathogen of humans: Chlamydia trachomatis. Science 282:754–759.
3. Fraser CM, Casjens S, Huang WM, Sutton GG, Clayton R, Lathigra R, White O, Ketchum KA, Dodson R, Hickey EK, Gwinn M, Dougherty B, Tomb JF, Fleischmann RD, Richardson D, Peterson J, Kerlavage AR, Quackenbush J, Salzberg S, Hanson M, van Vugt R, Palmer N, Adams MD, Gocayne J, Weidman J, Utterback T, Watthey L, McDonald L, Artiach P, Bowman C, Garland S, Fuji C, Cotton MD, Horst K, Roberts K, Hatch B, Smith HO, Venter JC. 1997. Genomic sequence of a Lyme disease spirochaete, Borrelia burgdorferi. Nature 390:580–586.
4. Kaneko T, Sato S, Kotani H, Tanaka A, Asamizu E, Nakamura Y, Miyajima N, Hirosawa M, Sugiura M, Sasamoto S, Kimura T, Hosouchi T, Matsuno A, Muraki A, Nakazaki N, Naruo K, Okumura S, Shimpo S, Takeuchi C, Wada T, Watanabe A, Yamada M, Yasuda M, Tabata S. 1996. Sequence analysis of the genome of the unicellular cyanobacterium Synechocystis sp. strain PCC6803. II. Sequence determination of the entire genome and assignment of potential protein-coding regions. DNA Res 3:109–136.
5. Bentley SD, Chater KF, Cerdeño-Tárraga A-M, Challis GL, Thomson NR, James KD, Harris DE, Quail MA, Kieser H, Harper D, Bateman A, Brown S, Chandra G, Chen CW, Collins M, Cronin A, Fraser A, Goble A, Hidalgo J, Hornsby T, Howarth S, Huang C-H, Kieser T, Larke L, Murphy L, Oliver K, O'Neil S, Rabbinowitsch E, Rajandream M-A, Rutherford K, Rutter S, Seeger K, Saunders D, Sharp S, Squares R, Squares S, Taylor K, Warren T, Wietzorrek A, Woodward J, Barrell BG, Parkhill J, Hopwood DA. 2002. Complete genome sequence of the model actinomycete Streptomyces coelicolor A3(2). Nature 417:141–147.
6. Vera-Cabrera L, Ortiz-Lopez R, Elizondo-Gonzalez R, Perez-Maya AA, Ocampo-Candiani J. 2012. Complete genome sequence of Nocardia brasiliensis HUJEG-1. J Bacteriol 194:2761–2762.
7. Cole ST, Brosch R, Parkhill J, Garnier T, Churcher C, Harris D, Gordon SV, Eiglmeier K, Gas S, Barry CE, Tekaia F, Badcock K, Basham D, Brown D, Chillingworth T, Connor R, Davies R, Devlin K, Feltwell T, Gentles S, Hamlin N, Holroyd S, Hornsby T, Jagels K, Krogh A, McLean J, Moule S, Murphy L, Oliver K, Osborne J, Quail MA, Rajandream MA, Rogers J, Rutter S, Seeger K, Skelton J, Squares R, Squares S, Sulston JE, Taylor K, Whitehead S, Barrell BG. 1998. Deciphering the biology of Mycobacterium tuberculosis from the complete genome sequence. Nature 393:537–544.
8. Sebaihia M, Peck MW, Minton NP, Thomson NR, Holden MTG, Mitchell WJ, Carter AT, Bentley SD, Mason DR, Crossman L, Paul CJ, Ivens A, Wells-Bennik MHJ, Davis IJ, Cerdeño-Tárraga AM, Churcher C, Quail MA, Chillingworth T, Feltwell T, Fraser A, Goodhead I, Hance Z, Jagels K, Larke N, Maddison M, Moule S, Mungall K, Norbertczak H, Rabbinowitsch E, Sanders M, Simmonds M, White B, Whithead S, Parkhill J. 2007. Genome sequence of a proteolytic (Group I) Clostridium botulinum strain Hall A and comparative analysis of the clostridial genomes. Genome Res 17:1082–1092.

9. Altermann E, Russell WM, Azcarate-Peril MA, Barrangou R, Buck BL, McAuliffe O, Souther N, Dobson A, Duong T, Callanan M, Lick S, Hamrick A, Cano R, Klaenhammer TR. 2005. Complete genome sequence of the probiotic lactic acid bacterium Lactobacillus acidophilus NCFM. Proc Natl Acad Sci U S A 102:3906–3912.
10. Bolotin A, Wincker P, Mauger S, Jaillon O, Malarme K, Weissenbach J, Ehrlich SD, Sorokin A. 2001. The complete genome sequence of the lactic acid bacterium Lactococcus lactis ssp. lactis IL1403. Genome Res 11:731–753.
11. Hoskins J, Alborn WE, Arnold J, Blaszczak LC, Burgett S, DeHoff BS, Estrem ST, Fritz L, Fu DJ, Fuller W, Geringer C, Gilmour R, Glass JS, Khoja H, Kraft AR, Lagace RE, LeBlanc DJ, Lee LN, Lefkowitz EJ, Lu J, Matsushima P, McAhren SM, McHenney M, McLeaster K, Mundy CW, Nicas TI, Norris FH, O'Gara M, Peery RB, Robertson GT, Rockey P, Sun PM, Winkler ME, Yang Y, Young-Bellido M, Zhao G, Zook CA, Baltz RH, Jaskunas SR, Rosteck PR, Skatrud PL, Glass JI. 2001. Genome of the bacterium Streptococcus pneumoniae strain R6. J Bacteriol 183:5709–5717.
12. Gillaspy AF, Worrell V, Orvis J, Roe BA, Dyer DW, Iandolo JJ. 2006. The Staphylococcus aureus NCTC 8325 Genome, p. 381–412. *In* Gram-Positive Pathogens. John Wiley & Sons, Ltd.
13. Feng L, Wang W, Cheng J, Ren Y, Zhao G, Gao C, Tang Y, Liu X, Han W, Peng X, Liu R, Wang L. 2007. Genome and proteome of long-chain alkane degrading Geobacillus thermodenitrificans NG80-2 isolated from a deep-subsurface oil reservoir. Proc Natl Acad Sci U S A 104:5602–5607.
14. Chen XH, Koumoutsi A, Scholz R, Eisenreich A, Schneider K, Heinemeyer I, Morgenstern B, Voss B, Hess WR, Reva O, Junge H, Voigt B, Jungblut PR, Vater J, Süssmuth R, Liesegang H, Strittmatter A, Gottschalk G, Borriss R. 2007. Comparative analysis of the complete genome sequence of the plant growth-promoting bacterium Bacillus amyloliquefaciens FZB42. Nat Biotechnol 25:1007–1014.
15. Kunst F, Ogasawara N, Moszer I, Albertini AM, Alloni G, Azevedo V, Bertero MG, Bessières P, Bolotin A, Borchert S, Borriss R, Boursier L, Brans A, Braun M, Brignell SC, Bron S, Brouillet S, Bruschi CV, Caldwell B, Capuano V, Carter NM, Choi SK, Cordani JJ, Connerton IF, Cummings NJ, Daniel RA, Denziot F, Devine KM, Düsterhöft A, Ehrlich SD, Emmerson PT, Entian KD, Errington J, Fabret C, Ferrari E, Foulger D, Fritz C, Fujita M, Fujita Y, Fuma S, Galizzi A, Galleron N, Ghim SY, Glaser P, Goffeau A, Golightly EJ, Grandi G, Guiseppi G, Guy BJ, Haga K, Haiech J, Harwood CR, Hènaut A, Hilbert H, Holsappel S, Hosono S, Hullo MF, Itaya M, Jones L, Joris B, Karamata D, Kasahara Y, Klaerr-Blanchard M, Klein C, Kobayashi Y, Koetter P, Koningstein G, Krogh S, Kumano M, Kurita K, Lapidus A, Lardinois S, Lauber J, Lazarevic V, Lee SM, Levine A, Liu H, Masuda S, Mauël C, Médigue C, Medina N, Mellado RP, Mizuno M, Moestl D, Nakai S, Noback M, Noone D, O'Reilly M, Ogawa K, Ogiwara A, Oudega B, Park SH, Parro V, Pohl TM, Portelle D, Porwollik S, Prescott AM, Presecan E, Pujic P, Purnelle B, Rapoport G, Rey M, Reynolds S, Rieger M, Rivolta C, Rocha E, Roche B, Rose M, Sadaie Y, Sato T, Scanlan E, Schleich S, Schroeter R, Scoffone F, Sekiguchi J, Sekowska A, Seror SJ, Serror P, Shin BS, Soldo B, Sorokin A, Tacconi E, Takagi T, Takahashi H, Takemaru K, Takeuchi M, Tamakoshi A, Tanaka T, Terpstra P, Togoni A, Tosato V, Uchiyama S, Vandebol M, Vannier F, Vassarotti A, Viari A, Wambutt R, Wedler H, Weitzenegger T, Winters P, Wipat A, Yamamoto H, Yamane K, Yasumoto K, Yata K, Yoshida K, Yoshikawa HF, Zumstein E, Yoshikawa H, Danchin A. 1997. The complete genome sequence of the gram-positive bacterium Bacillus subtilis. Nature 390:249–256.

16. Rendulic S, Jagtap P, Rosinus A, Eppinger M, Baar C, Lanz C, Keller H, Lambert C, Evans KJ, Goesmann A, Meyer F, Sockett RE, Schuster SC. 2004. A predator unmasked: life cycle of Bdellovibrio bacteriovorus from a genomic perspective. Science 303:689–692.
17. Tomb JF, White O, Kerlavage AR, Clayton RA, Sutton GG, Fleischmann RD, Ketchum KA, Klenk HP, Gill S, Dougherty BA, Nelson K, Quackenbush J, Zhou L, Kirkness EF, Peterson S, Loftus B, Richardson D, Dodson R, Khalak HG, Glodek A, McKenney K, Fitzegerald LM, Lee N, Adams MD, Hickey EK, Berg DE, Gocayne JD, Utterback TR, Peterson JD, Kelley JM, Cotton MD, Weidman JM, Fujii C, Bowman C, Watthey L, Wallin E, Hayes WS, Borodovsky M, Karp PD, Smith HO, Fraser CM, Venter JC. 1997. The complete genome sequence of the gastric pathogen Helicobacter pylori. Nature 388:539–547.
18. Tettelin H, Saunders NJ, Heidelberg J, Jeffries AC, Nelson KE, Eisen JA, Ketchum KA, Hood DW, Peden JF, Dodson RJ, Nelson WC, Gwinn ML, DeBoy R, Peterson JD, Hickey EK, Haft DH, Salzberg SL, White O, Fleischmann RD, Dougherty BA, Mason T, Ciecko A, Parksey DS, Blair E, Cittone H, Clark EB, Cotton MD, Utterback TR, Khouri H, Qin H, Vamathevan J, Gill J, Scarlato V, Masignani V, Pizza M, Grandi G, Sun L, Smith HO, Fraser CM, Moxon ER, Rappuoli R, Venter JC. 2000. Complete genome sequence of Neisseria meningitidis serogroup B strain MC58. Science 287:1809–1815.
19. Parkhill J, Sebaihia M, Preston A, Murphy LD, Thomson N, Harris DE, Holden MTG, Churcher CM, Bentley SD, Mungall KL, Cerdeño-Tárraga AM, Temple L, James K, Harris B, Quail MA, Achtman M, Atkin R, Baker S, Basham D, Bason N, Cherevach I, Chillingworth T, Collins M, Cronin A, Davis P, Doggett J, Feltwell T, Goble A, Hamlin N, Hauser H, Holroyd S, Jagels K, Leather S, Moule S, Norberczak H, O'Neil S, Ormond D, Price C, Rabbinowitsch E, Rutter S, Sanders M, Saunders D, Seeger K, Sharp S, Simmonds M, Skelton J, Squares R, Squares S, Stevens K, Unwin L, Whitehead S, Barrell BG, Maskell DJ. 2003. Comparative analysis of the genome sequences of Bordetella pertussis, Bordetella parapertussis and Bordetella bronchiseptica. Nat Genet 35:32–40.
20. Ma Y-F, Zhang Y, Zhang J-Y, Chen D-W, Zhu Y, Zheng H, Wang S-Y, Jiang C-Y, Zhao G-P, Liu S-J. 2009. The complete genome of Comamonas testosteroni reveals its genetic adaptations to changing environments. Appl Environ Microbiol 75:6812–6819.
21. Salanoubat M, Genin S, Artiguenave F, Gouzy J, Mangenot S, Arlat M, Billault A, Brottier P, Camus JC, Cattolico L, Chandler M, Choisne N, Claudel-Renard C, Cunnac S, Demange N, Gaspin C, Lavie M, Moisan A, Robert C, Saurin W, Schiex T, Siguier P, Thébault P, Whalen M, Wincker P, Levy M, Weissenbach J, Boucher CA. 2002. Genome sequence of the plant pathogen Ralstonia solanacearum. Nature 415:497–502.
22. Miller TR, Delcher AL, Salzberg SL, Saunders E, Detter JC, Halden RU. 2010. Genome sequence of the dioxin-mineralizing bacterium Sphingomonas wittichii RW1. J Bacteriol 192:6101–6102.
23. Nierman WC, Feldblyum TV, Laub MT, Paulsen IT, Nelson KE, Eisen J, Heidelberg JF, Alley MRK, Ohta N, Maddock JR, Potocka I, Nelson WC, Newton A, Stephens C, Phadke ND, Ely B, DeBoy RT, Dodson RJ, Durkin AS, Gwinn ML, Haft DH, Kolonay JF, Smit J, Craven MB, Khouri H, Shetty J, Berry K, Utterback T, Tran K, Wolf A, Vamathevan J, Ermolaeva M, White O, Salzberg SL, Venter JC, Shapiro L, Fraser CM. 2001. Complete genome sequence of Caulobacter crescentus. Proc Natl Acad Sci U S A 98:4136–4141.

24. Choudhary M, Zanhua X, Fu YX, Kaplan S. 2007. Genome analyses of three strains of Rhodobacter sphaeroides: evidence of rapid evolution of chromosome II. J Bacteriol 189:1914–1921.
25. Dos Santos Ferreira N, Hayashi Sant' Anna F, Massena Reis V, Ambrosini A, Gazolla Volpiano C, Rothballer M, Schwab S, Baura VA, Balsanelli E, Pedrosa F de O, Pereira Passaglia LM, Maltempi de Souza E, Hartmann A, Cassan F, Zilli JE. 2020. Genome-based reclassification of Azospirillum brasilense Sp245 as the type strain of Azospirillum baldaniorum sp. nov. Int J Syst Evol Microbiol 70:6203–6212.
26. Suárez-Esquivel M, Ruiz-Villalobos N, Castillo-Zeledón A, Jiménez-Rojas C, Roop II RM, Comerci DJ, Barquero-Calvo E, Chacón-Díaz C, Caswell CC, Baker KS, Chaves-Olarte E, Thomson NR, Moreno E, Letesson JJ, De Bolle X, Guzmán-Verri C. 2016. Brucella abortus Strain 2308 Wisconsin Genome: Importance of the Definition of Reference Strains. Front Microbiol 7:1557.
27. Capela D, Barloy-Hubler F, Gouzy J, Bothe G, Ampe F, Batut J, Boistard P, Becker A, Boutry M, Cadieu E, Dréano S, Gloux S, Godrie T, Goffeau A, Kahn D, Kiss E, Lelaure V, Masuy D, Pohl T, Portetelle D, Pühler A, Purnelle B, Ramsperger U, Renard C, Thébault P, Vandenbol M, Weidner S, Galibert F. 2001. Analysis of the chromosome sequence of the legume symbiont Sinorhizobium meliloti strain 1021. Proc Natl Acad Sci U S A 98:9877–9882.
28. Wood DW, Setubal JC, Kaul R, Monks DE, Kitajima JP, Okura VK, Zhou Y, Chen L, Wood GE, Almeida NF, Woo L, Chen Y, Paulsen IT, Eisen JA, Karp PD, Bovee D, Chapman P, Clendenning J, Deatherage G, Gillet W, Grant C, Kutyavin T, Levy R, Li MJ, McClelland E, Palmieri A, Raymond C, Rouse G, Saenphimmachak C, Wu Z, Romero P, Gordon D, Zhang S, Yoo H, Tao Y, Biddle P, Jung M, Krespan W, Perry M, Gordon-Kamm B, Liao L, Kim S, Hendrick C, Zhao ZY, Dolan M, Chumley F, Tingey SV, Tomb JF, Gordon MP, Olson MV, Nester EW. 2001. The genome of the natural genetic engineer Agrobacterium tumefaciens C58. Science 294:2317–2323.
29. Adams MD, Goglin K, Molyneaux N, Hujer KM, Lavender H, Jamison JJ, MacDonald IJ, Martin KM, Russo T, Campagnari AA, Hujer AM, Bonomo RA, Gill SR. 2008. Comparative genome sequence analysis of multidrug-resistant Acinetobacter baumannii. J Bacteriol 190:8053–8064.
30. da Silva ACR, Ferro JA, Reinach FC, Farah CS, Furlan LR, Quaggio RB, Monteiro-Vitorello CB, Van Sluys MA, Almeida NF, Alves LMC, do Amaral AM, Bertolini MC, Camargo LEA, Camarotte G, Cannavan F, Cardozo J, Chambergo F, Ciapina LP, Cicarelli RMB, Coutinho LL, Cursino-Santos JR, El-Dorry H, Faria JB, Ferreira AJS, Ferreira RCC, Ferro MIT, Formighieri EF, Franco MC, Greggio CC, Gruber A, Katsuyama AM, Kishi LT, Leite RP, Lemos EGM, Lemos MVF, Locali EC, Machado MA, Madeira AMBN, Martinez-Rossi NM, Martins EC, Meidanis J, Menck CFM, Miyaki CY, Moon DH, Moreira LM, Novo MTM, Okura VK, Oliveira MC, Oliveira VR, Pereira HA, Rossi A, Sena J a. D, Silva C, de Souza RF, Spinola L a. F, Takita MA, Tamura RE, Teixeira EC, Tezza RID, Trindade dos Santos M, Truffi D, Tsai SM, White FF, Setubal JC, Kitajima JP. 2002. Comparison of the genomes of two Xanthomonas pathogens with differing host specificities. Nature 417:459–463.
31. Chien M, Morozova I, Shi S, Sheng H, Chen J, Gomez SM, Asamani G, Hill K, Nuara J, Feder M, Rineer J, Greenberg JJ, Steshenko V, Park SH, Zhao B, Teplitskaya E, Edwards JR, Pampou S, Georghiou A, Chou I-C, Iannuccilli W, Ulz ME, Kim DH, Geringer-Sameth A, Goldsberry C, Morozov P, Fischer SG, Segal G, Qu X, Rzhetsky A, Zhang P, Cayanis E, De Jong PJ, Ju J, Kalachikov S, Shuman HA, Russo JJ. 2004. The

genomic sequence of the accidental pathogen Legionella pneumophila. Science 305:1966–1968.

32. Heidelberg JF, Paulsen IT, Nelson KE, Gaidos EJ, Nelson WC, Read TD, Eisen JA, Seshadri R, Ward N, Methe B, Clayton RA, Meyer T, Tsapin A, Scott J, Beanan M, Brinkac L, Daugherty S, DeBoy RT, Dodson RJ, Durkin AS, Haft DH, Kolonay JF, Madupu R, Peterson JD, Umayam LA, White O, Wolf AM, Vamathevan J, Weidman J, Impraim M, Lee K, Berry K, Lee C, Mueller J, Khouri H, Gill J, Utterback TR, McDonald LA, Feldblyum TV, Smith HO, Venter JC, Nealson KH, Fraser CM. 2002. Genome sequence of the dissimilatory metal ion-reducing bacterium Shewanella oneidensis. Nat Biotechnol 20:1118–1123.
33. Mandel MJ, Stabb EV, Ruby EG. 2008. Comparative genomics-based investigation of resequencing targets in Vibrio fischeri: Focus on point miscalls and artefactual expansions. BMC Genomics 9:138.
34. Heidelberg JF, Eisen JA, Nelson WC, Clayton RA, Gwinn ML, Dodson RJ, Haft DH, Hickey EK, Peterson JD, Umayam L, Gill SR, Nelson KE, Read TD, Tettelin H, Richardson D, Ermolaeva MD, Vamathevan J, Bass S, Qin H, Dragoi I, Sellers P, McDonald L, Utterback T, Fleishmann RD, Nierman WC, White O, Salzberg SL, Smith HO, Colwell RR, Mekalanos JJ, Venter JC, Fraser CM. 2000. DNA sequence of both chromosomes of the cholera pathogen Vibrio cholerae. Nature 406:477–483.
35. Setubal JC, dos Santos P, Goldman BS, Ertesvåg H, Espin G, Rubio LM, Valla S, Almeida NF, Balasubramanian D, Cromes L, Curatti L, Du Z, Godsy E, Goodner B, Hellner-Burris K, Hernandez JA, Houmiel K, Imperial J, Kennedy C, Larson TJ, Latreille P, Ligon LS, Lu J, Maerk M, Miller NM, Norton S, O'Carroll IP, Paulsen I, Raulfs EC, Roemer R, Rosser J, Segura D, Slater S, Stricklin SL, Studholme DJ, Sun J, Viana CJ, Wallin E, Wang B, Wheeler C, Zhu H, Dean DR, Dixon R, Wood D. 2009. Genome sequence of Azotobacter vinelandii, an obligate aerobe specialized to support diverse anaerobic metabolic processes. J Bacteriol 191:4534–4545.
36. Buell CR, Joardar V, Lindeberg M, Selengut J, Paulsen IT, Gwinn ML, Dodson RJ, Deboy RT, Durkin AS, Kolonay JF, Madupu R, Daugherty S, Brinkac L, Beanan MJ, Haft DH, Nelson WC, Davidsen T, Zafar N, Zhou L, Liu J, Yuan Q, Khouri H, Fedorova N, Tran B, Russell D, Berry K, Utterback T, Van Aken SE, Feldblyum TV, D'Ascenzo M, Deng W-L, Ramos AR, Alfano JR, Cartinhour S, Chatterjee AK, Delaney TP, Lazarowitz SG, Martin GB, Schneider DJ, Tang X, Bender CL, White O, Fraser CM, Collmer A. 2003. The complete genome sequence of the Arabidopsis and tomato pathogen Pseudomonas syringae pv. tomato DC3000. Proc Natl Acad Sci U S A 100:10181–10186.
37. Silby MW, Cerdeño-Tárraga AM, Vernikos GS, Giddens SR, Jackson RW, Preston GM, Zhang X-X, Moon CD, Gehrig SM, Godfrey SAC, Knight CG, Malone JG, Robinson Z, Spiers AJ, Harris S, Challis GL, Yaxley AM, Harris D, Seeger K, Murphy L, Rutter S, Squares R, Quail MA, Saunders E, Mavromatis K, Brettin TS, Bentley SD, Hothersall J, Stephens E, Thomas CM, Parkhill J, Levy SB, Rainey PB, Thomson NR. 2009. Genomic and genetic analyses of diversity and plant interactions of Pseudomonas fluorescens. Genome Biol 10:R51.
38. Belda E, van Heck RGA, José Lopez-Sanchez M, Cruveiller S, Barbe V, Fraser C, Klenk H-P, Petersen J, Morgat A, Nikel PI, Vallenet D, Rouy Z, Sekowska A, Martins Dos Santos VAP, de Lorenzo V, Danchin A, Médigue C. 2016. The revisited genome of Pseudomonas putida KT2440 enlightens its value as a robust metabolic chassis. Environ Microbiol 18:3403–3424.

39. Stover CK, Pham XQ, Erwin AL, Mizoguchi SD, Warrener P, Hickey MJ, Brinkman FS, Hufnagle WO, Kowalik DJ, Lagrou M, Garber RL, Goltry L, Tolentino E, Westbrock-Wadman S, Yuan Y, Brody LL, Coulter SN, Folger KR, Kas A, Larbig K, Lim R, Smith K, Spencer D, Wong GK, Wu Z, Paulsen IT, Reizer J, Saier MH, Hancock RE, Lory S, Olson MV. 2000. Complete genome sequence of Pseudomonas aeruginosa PAO1, an opportunistic pathogen. Nature 406:959–964.
40. Bell KS, Sebaihia M, Pritchard L, Holden MTG, Hyman LJ, Holeva MC, Thomson NR, Bentley SD, Churcher LJC, Mungall K, Atkin R, Bason N, Brooks K, Chillingworth T, Clark K, Doggett J, Fraser A, Hance Z, Hauser H, Jagels K, Moule S, Norbertczak H, Ormond D, Price C, Quail MA, Sanders M, Walker D, Whitehead S, Salmond GPC, Birch PRJ, Parkhill J, Toth IK. 2004. Genome sequence of the enterobacterial phytopathogen Erwinia carotovora subsp. atroseptica and characterization of virulence factors. Proc Natl Acad Sci U S A 101:11105–11110.
41. Duchaud E, Rusniok C, Frangeul L, Buchrieser C, Givaudan A, Taourit S, Bocs S, Boursaux-Eude C, Chandler M, Charles J-F, Dassa E, Derose R, Derzelle S, Freyssinet G, Gaudriault S, Médigue C, Lanois A, Powell K, Siguier P, Vincent R, Wingate V, Zouine M, Glaser P, Boemare N, Danchin A, Kunst F. 2003. The genome sequence of the entomopathogenic bacterium Photorhabdus luminescens. Nat Biotechnol 21:1307–1313.
42. Chain PSG, Hu P, Malfatti SA, Radnedge L, Larimer F, Vergez LM, Worsham P, Chu MC, Andersen GL. 2006. Complete genome sequence of Yersinia pestis strains Antiqua and Nepal516: evidence of gene reduction in an emerging pathogen. J Bacteriol 188:4453–4463.
43. Müller H, Fürnkranz M, Grube M, Berg G. 2013. Genome Sequence of Serratia plymuthica Strain S13, an Endophyte with Germination- and Plant-Growth-Promoting Activity from the Flower of Styrian Oil Pumpkin. Genome Announc 1:e00594-13.
44. McClelland M, Sanderson KE, Spieth J, Clifton SW, Latreille P, Courtney L, Porwollik S, Ali J, Dante M, Du F, Hou S, Layman D, Leonard S, Nguyen C, Scott K, Holmes A, Grewal N, Mulvaney E, Ryan E, Sun H, Florea L, Miller W, Stoneking T, Nhan M, Waterston R, Wilson RK. 2001. Complete genome sequence of Salmonella enterica serovar Typhimurium LT2. Nature 413:852–856.
45. Blattner FR, Plunkett G, Bloch CA, Perna NT, Burland V, Riley M, Collado-Vides J, Glasner JD, Rode CK, Mayhew GF, Gregor J, Davis NW, Kirkpatrick HA, Goeden MA, Rose DJ, Mau B, Shao Y. 1997. The complete genome sequence of Escherichia coli K-12. Science 277:1453–1462.
46. Liu P, Li P, Jiang X, Bi D, Xie Y, Tai C, Deng Z, Rajakumar K, Ou H-Y. 2012. Complete genome sequence of Klebsiella pneumoniae subsp. pneumoniae HS11286, a multidrug-resistant strain isolated from human sputum. J Bacteriol 194:1841–1842.

Table S2) The SBP repertoire of the 49 bacterial model strains.

lignad family correctly predicted by TransportDB

ligand family not correctly predicted by TransportDB

single lignad predicted and experimentally determined ligands belong to the ligand family of the predicted compound

predicted ligand family is similar to the family of experimentally determined ligand(s)

***Rhodobacter sphaeroides* ATCC 17025**					
Number of ORFs in the proteome: 4208					
Number of SBPs: 78 (1.85% of total)					
Lifestyle Summary: FRESH/MARINE WATERS					
**SeqID**	**Gene Locus**	**Pfam**	**Ligand prediction by TransportDB**	**Experimentally identified ligands**	**References/pdb accession codes**
A4WVU2	Rsph17025_2619	PF13407	xylose		
A4WP86	Rsph17025_0290	PF16868	phosphonates		
A4WWG6	Rsph17025_2844	PF00496	dipeptide/oligopeptide		
A4WSS3	Rsph17025_1543	PF03480	C4-dicarboxylate		
A4X0L4	Rsph17025_4333	PF03180	methionine		
A4WWG5	Rsph17025_2843	PF00496	dipeptide/oligopeptide		
A4WUI2	Rsph17025_2156	PF00496	dipeptide/oligopeptide		
A4WSB1	Rsph17025_1378	PF13416	spermidine/putrescine		
A4WYU1	Rsph17025_3688	PF16868	phosphonate		
A4WQM8	Rsph17025_0786	PF03180	methionine		
A4WYI9	Rsph17025_3584	PF13416	sugar		
A4WXN7	Rsph17025_3267	PF16868	Unclassified		
A4WP45	Rsph17025_0249	PF01297	manganese/zinc ion		
A4WSN6	Rsph17025_1506	PF00496	oligopeptide		
A4WXX2	Rsph17025_3355	PF13407	rhamnose		
A4WWF0	Rsph17025_2828	PF04069	glycine betaine		
A4WR14	Rsph17025_0926	PF13458	leucine/valine		
A4WZK3	Rsph17025_3963	PF00496	dipeptide/oligopeptide		
A4WVH7	Rsph17025_2503	PF00497	amino acid (glutamine/glutamate/aspartate?)		

A4WU89	Rsph17025_2062	PF00497	amino acid (glutamine/glutamate/aspartate?)		
A4WYH5	Rsph17025_3570	PF03480	C4-dicarboxylate		
A4WWL2	Rsph17025_2890	PF00496	oligopeptide		
A4WXV8	Rsph17025_3341	PF03480	C4-dicarboxylate		
A4WUY8	Rsph17025_2313	PF04069	choline		
A4WXN8	Rsph17025_3268	PF13458	urea		
A4WQ28	Rsph17025_0586	PF09084	nitrate/sulfonate/taurine		
A4WQK6	Rsph17025_0764	PF09084	nitrate/sulfonate/taurine		
A4WSH6	Rsph17025_1446	PF13416	spermidine/putrescine		
A4WQJ3	Rsph17025_0751	PF09084	nitrate/sulfonate/taurine		
A4WSB6	Rsph17025_1383	PF01497	iron-hydroxamate		
A4WP30	Rsph17025_0234	PF13407	ribose		
A4WTV3	Rsph17025_1926	PF09084	nitrate/sulfonate/taurine		
A4WS65	Rsph17025_1330	PF13416	spermidine/putrescine		
A4WSJ6	Rsph17025_1466	PF09084	nitrate/sulfonate/taurine		
A4WXE1	Rsph17025_3171	PF00497	amino acid (glutamine/glutamate/aspartate?)		
A4WTX0	Rsph17025_1943	PF00497	amino acid (glutamine/glutamate/aspartate?)		
A4WQK5	Rsph17025_0763	PF09084	glycine betaine		
A4WPW8	Rsph17025_0526	PF00496	oligopeptide		
A4WXU8	Rsph17025_3331	PF01497	iron-hydroxamate		
A4WXQ5	Rsph17025_3285	PF13416	sugar		
A4WP39	Rsph17025_0243	PF03480	C4-dicarboxylate		
A4WRN4	Rsph17025_1147	PF01547	sugar		
A4WXR2	Rsph17025_3292	PF13458	urea		
A4WPG4	Rsph17025_0372	PF00496	dipeptide/oligopeptide		
A4WXT9	Rsph17025_3320	PF05048	Not annotated in TransportDB		
A4WZI2	Rsph17025_3942	PF02608	sugar		
A4WT60	Rsph17025_1681	PF01547	sugar		
A4WW44	Rsph17025_2721	PF13458	urea		
A4WW08	Rsph17025_2685	PF13407	xylose		

A4X0E7	Rsph17025_4264	PF01497	iron-hydroxamate		
A4WXM8	Rsph17025_3258	PF03480	C4-dicarboxylate		
A4WTU6	Rsph17025_1919	PF12849	phosphate		
A4WYQ5	Rsph17025_3651	PF01547	sugar		
A4WUP7	Rsph17025_2221	PF13458	leucine/valine		
A4WXC1	Rsph17025_3151	PF01297	zinc ion		
A4WUV8	Rsph17025_2282	PF02608	sugar		
A4WZE2	Rsph17025_3901	PF13458	urea		
A4WYF8	Rsph17025_3553	PF13416	sugar		
A4WR38	Rsph17025_0951	PF02608	sugar		
A4WXC9	Rsph17025_3159	PF13416	iron(III)		
A4WT65	Rsph17025_1686	PF03480	C4-dicarboxylate		
A4WQM5	Rsph17025_0783	PF03180	methionine		
A4WPR2	Rsph17025_0470	PF13458	leucine/valine		
A4WXR5	Rsph17025_3295	PF16868	phosphonates		
A4WV85	Rsph17025_2411	PF03480	C4-dicarboxylate		
A4WZ00	Rsph17025_3750	PF03180	methionine		
A4WQV8	Rsph17025_0869	PF00497	amino acid (glutamine/glutamate/aspartate?)		
A4WU71	Rsph17025_2044	PF03480	C4-dicarboxylate		
A4WWU7	Rsph17025_2975	PF09084	nitrate/sulfonate/taurine		
A4WSY8	Rsph17025_1609	PF03480	C4-dicarboxylate		
A4WQL9	Rsph17025_0777	PF09084	nitrate/sulfonate/taurine		
A4WSS2	Rsph17025_1542	PF03480	C4-dicarboxylate		
A4WY04	Rsph17025_3391	PF00496	dipeptide/oligopeptide		
A4WTW8	Rsph17025_1941	PF13458	leucine/valine		
A4WZA8	Rsph17025_3867	PF03480	C4-dicarboxylate		
A4WST0	Rsph17025_1550	PF03480	C4-dicarboxylate		
A4WXP5	Rsph17025_3275	PF00496	dipeptide/oligopeptide		
A4WQA9	Rsph17025_0667	PF13416	spermidine/putrescine		
***Neisseria meningitidis* MC58**					
Number of ORFs in the proteome: 2001					

Number of SBPs: 11 (0.55% of total)					
Lifestyle Summary: HUMAN/ANIMAL PATHOGEN					
**SeqID**	**Gene Locus**	**Pfam Code**	**Ligand prediction by TransportDB**	**Experimentally identified ligands**	**References/pdb accession codes**
P0A0Y4	NMB0634	PF01547	iron(III)		
Q9JYG7	NMB1594	PF13416	spermidine/putrescine		
Q9K0L5	NMB0578	PF05048	Not annotated in TransportDB		
Q9K0K8	NMB0586	PF01297	manganese/zinc ion		
Q9K0H8	NMB0623	PF13416	spermidine/putrescine		
Q9K0U8	NMB0462	PF13416	spermidine/putrescine		
Q9JYF0	NMB1612	PF00497	amino acid (glutamine/glutamate/aspartate?)		
Q9K035	NMB0787	PF00497	amino acid (glutamine/glutamate/aspartate?)		
Q7DD63	NMB1946	PF03180	methionine		
Q9JXU5	NMB1880	PF01497	iron-hydroxamate		
Q9JXL2	NMB1989	PF01497	iron-hydroxamate		
***Helicobacter pylori* 26695**					
Number of ORFs in the proteome: 1552					
Number of SBPs: 7 (0.45% of total)					
Lifestyle Summary: HUMAN/ANIMAL PATHOGEN					
**SeqID**	**Gene Locus**	**Pfam Code**	**Ligand prediction by TransportDB**	**Experimentally identified ligands**	**References/pdb accession codes**
O25845	HP_1252	PF00496	oligopeptide		
O26084	HP_1564	PF03180	methionine		
O25786	HP_1172	PF00497	amino acid (glutamine/glutamate/aspartate?)		
O26083	HP_1562	PF01497	iron-hydroxamate		
O25069	HP_0298	PF00496	dipeptide/oligopeptide		
O26082	HP_1561	PF01497	iron-hydroxamate		
O25594	HP_0940	PF00497	amino acid (glutamine/glutamate/aspartate?)		

***Chlamydia trachomatis* D/UW-3/CX**					
Number of ORFss in the proteome: 895					
Number of SBPs: 9 (1.01% of total)					
Lifestyle Summary: HUMAN/ANIMAL PATHOGEN					
**SeqID**	**Gene Locus**	**Pfam Code**	**Ligand prediction by TransportDB**	**Experimentally identified ligands**	**References/pdb accession codes**
Q9S529	CT_067	PF01297	manganese/zinc ion	Fe^{3+}, Mn^{2+}, Ni^{2+}, Fe^{2+}	PDB: 6NSI; DOI: 10.1128/JB.00580-19
O84420	CT_415	PF01297	manganese/zinc ion		
O84385	CT_381	PF00497	amino acid (glutamine/glutamate/aspartate?)	Arginine	DOI: 10.1074/jbc.M110.118513
O84396	CT_391	PF04392	Unclassified		
O84178	CT_175	PF00496	oligopeptide		
O84493	CT_486	PF00497	amino acid (glutamine/glutamate/aspartate?)		
O84201	CT_198	PF00496	oligopeptide		
O84486	CT_480	PF00496	dipeptide/oligopeptide		
O84141	CT_139	PF00496	dipeptide/oligopeptide		
***Pseudomonas putida* KT2440**					
Number of ORFs in the proteome: 5527					
Number of SBPs: 85 (1.54% of total)					
Lifestyle Summary: SOIL					
**SeqID**	**Gene Locus**	**Pfam Code**	**Ligand prediction by TransportDB**	**Experimentally identified ligands**	**References/pdb accession codes**
Q88DF8	PP_4867	PF13458	leucine/valine		
Q88DS5	PP_4748	PF00497	amino acid (glutamine/glutamate/aspartate?)		
Q88Q41	PP_0657	PF00497	amino acid (glutamine/glutamate/aspartate?)		
Q88KM6	PP_2264	PF13416	sugar		
Q88Q76	PP_0619	PF13458	urea		

Q88CK0	PP_5180	PF13416	spermidine/putrescine		
Q88H37	PP_3528	PF09084	glycine betaine		
Q88N00	PP_1418	PF03401	tricarboxylate		
Q88CJ9	PP_5181	PF13416	spermidine/putrescine		
Q88K38	PP_2454	PF13407	ribose		
Q88P38	PP_1015	PF13416	sugar		
Q88HY0	PP_3223	PF00496	dipeptide/oligopeptide		
Q88RQ3	PP_0076	PF04069	choline		
Q88RA0	PP_0233	PF09084	glycine betaine		
Q88FF1	PP_4146	PF00496	oligopeptide		
Q88RB2	PP_0221	PF03180	methionine		
Q88KB8	PP_2372	PF13407	ribose		
Q88CM3	PP_5157	PF00497	amino acid (glutamine/glutamate/aspartate?)		
Q88QS6	PP_0412	PF13416	spermidine/putrescine		
Q88HY6	PP_3217	PF09084	nitrate/sulfonate/taurine		
Q88CL5	PP_5165	PF03180	methionine		
Q88RA6	PP_0227	PF00497	amino acid (glutamine/glutamate/aspartate?)		
Q88FX3	PP_3954	PF16868	Unclassified		
Q88MT2	PP_1486	PF13416	spermidine/putrescine		
Q88PG5	PP_0885	PF00496	dipeptide/oligopeptide		
Q88C54	PP_5329	PF12849	phosphate		
Q88R96	PP_0237	PF09084	glycine betaine		
Q88HX4	PP_3229	PF09084	nitrate/sulfonate/taurine		
Q88J92	PP_2757	PF13407	ribose		
Q88FF0	PP_4147	PF00496	oligopeptide		
Q88GC3	PP_3801	PF01297	manganese/zinc ion		
Q88PG6	PP_0884	PF00496	dipeptide/oligopeptide		
Q88JJ3	PP_2656	PF12849	phosphate		
Q88J91	PP_2758	PF13407	ribose		
Q88I54	PP_3147	PF13416	spermidine/putrescine		
Q88H07	PP_3558	PF04069	glycine betaine		

Q88F29	PP_4270	PF00497	amino acid (glutamine/glutamate/aspartate?)		
Q88IX9	PP_2870	PF13416	spermidine/putrescine		
Q88JQ8	PP_2591	PF01497	iron-hydroxamate		
Q88PI0	PP_0870	PF04069	glycine betaine		
Q88NY2	PP_1071	PF00497	amino acid (glutamine/glutamate/aspartate?)		
Q88QG9	PP_0524	PF01497	iron-hydroxamate		
Q88N87	PP_1325	PF04348	Not annotated in TransportDB		
Q88RK9	PP_0120	PF01297	zinc ion		
Q88HL4	PP_3342	PF00496	nickel		
Q88GB0	PP_3814	PF13416	spermidine/putrescine		
Q88NB5	PP_1297	PF00497	amino acid (glutamine/glutamate/aspartate?)		
Q88R38	PP_0296	PF04069	choline		
Q88D05	PP_5024	PF00497	amino acid (glutamine/glutamate/aspartate?)		
Q88IC1	PP_3078	PF00496	oligopeptide		
Q88D72	PP_4955	PF00497	amino acid (glutamine/glutamate/aspartate?)		
Q88K73	PP_2418	PF01497	cobalamin		
Q88G80	PP_3845	PF13416	spermidine/putrescine		
Q88EH7	PP_4486	PF00497	amino acid (glutamine/glutamate/aspartate?)		
Q88NR4	PP_1141	PF13458	leucine/valine		
Q88GX4	PP_3593	PF00497	amino acid (glutamine/glutamate/aspartate?)		
Q88C42	PP_5341	PF13416	spermidine/putrescine		
Q88PH7	PP_0873	PF13416	spermidine/putrescine		
Q88HY9	PP_3213	PF09084	nitrate/sulfonate/taurine		
Q88J74	PP_2775	PF04069	glycine betaine		
Q88R30	PP_0304	PF04069	choline		
Q88RL7	PP_0112	PF03180	methionine		

Q88EM6	PP_4428	PF00497	amino acid (glutamine/glutamate/aspartate?)		
Q88DX9	PP_4689	PF01497	iron-hydroxamate		
Q88R52	PP_0282	PF00497	amino acid (glutamine/glutamate/aspartate?)		
Q88C98	PP_5283	PF00496	dipeptide/oligopeptide		
Q88EK4	PP_4458	PF00496	dipeptide/oligopeptide		
Q88D49	PP_4979	PF00497	amino acid (glutamine/glutamate/aspartate?)		
Q88J97	PP_2752	PF13458	leucine/valine		
Q88QH2	PP_0521	PF00497	amino acid (glutamine/glutamate/aspartate?)		
Q88PG8	PP_0882	PF00496	dipeptide/oligopeptide		
Q88J79	PP_2770	PF13458	branched-chain amino acid		
Q88GU3	PP_3624	PF00497	amino acid (glutamine/glutamate/aspartate?)		
Q88GJ3	PP_3729	PF00497	amino acid (glutamine/glutamate/aspartate?)		
Q88M34	PP_1741	PF04069	glycine betaine		
Q88HX5	PP_3228	PF09084	nitrate/sulfonate/taurine		
Q88KU4	PP_2195	PF13416	spermidine/putrescine		
Q88NN8	PP_1169	PF03480	C4-dicarboxylate		
Q88F98	PP_4200	PF00497	amino acid (glutamine/glutamate/aspartate?)		
Q88F53	PP_4246	PF00497	amino acid (glutamine/glutamate/aspartate?)		
Q88GC0	PP_3804	PF01297	manganese/zinc ion		
Q88J98	PP_2751	PF13458	leucine/valine		
Q88GK2	PP_3719	PF13416	spermidine/putrescine		
A0A140FW9	PP_5538	PF13458	leucine/valine		
Q88JH3	PP_2676	PF00497	amino acid (glutamine/glutamate/aspartate?)		

Vibrio cholerae O1 biovar El Tor str. N16961					
Number of ORFs in the proteome: 3782					
Number of SBPs: 51 (1.35% of total)					
Lifestyle Summary: HUMAN/ANIMAL PATHOGEN					
**SeqID**	**Gene Locus**	**Pfam Code**	**Ligand prediction by TransportDB**	**Experimentally identified ligands**	**References/pdb accession codes**
Q9KR64	VC_1779	PF03480	C4-dicarboxylate	Sialic acid	DOI: 10.1074/jbc.M111.281030; PDB: 5LTC
Q9KTJ7	VC_0905	PF03180	methionine		
Q9KUE0	VC_0581	PF04348	leucine/valine		
Q9KU25	VC_0704	PF13416	spermidine/putrescine	Norspermidine	DOI: 10.1128/JB.187.21.7434-7443.2005
Q9KQR9	VC_1929	PF03480	C4-dicarboxylate	Sialic acid	DOI: 10.1099/mic.0.059659-0
Q9KLK6	VC_A0737	PF13407	xylose		
Q9KPI6	VC_2381	PF01497	iron-hydroxamate		
Q9KUA3	VC_0620	PF00496	dipeptide/oligopeptide	Chitin, 2-acetamido-2-deoxy-beta-D-glucopyranose-(1-4)-2-acetamido-2-deoxy-beta-D-glucopyranose	PDB: 1ZU0, 4GFR,
Q9KS97	VC_1362	PF00497	amino acid (glutamine/glutamate/aspartate?)		
Q9KUT5	VC_0430	PF16868	glycine betaine		
Q9KKR3	VC_A1039	PF00497	amino acid (glutamine/glutamate/aspartate?)		
Q9KVE4	VC_0202	PF01497	iron-hydroxamate		
Q9KKK6	VC_A1098	PF00496	dipeptide/oligopeptide		
Q9KT04	VC_1101	PF04392	Unclassified	Phenylalanine	PDB: 3LKV
Q9KS36	VC_1425	PF13416	spermidine/putrescine		
Q9KRG2	VC_1680	PF00496	oligopeptide		
Q9KS37	VC_1424	PF13416	spermidine/putrescine		
Q9KN35	VC_A0130	PF13407	ribose		
Q9KN95	VC_A0070	PF12849	phosphate		

Q9KN22	VC_A0144	PF16868	phosphonates		
Q9KM06	VC_A0583	PF00497	amino acid (glutamine/glutamate/aspartate?)		
Q9KRT7	VC_1549	PF13416	sugar		
Q9KT14	VC_1091	PF00496	oligopeptide		
Q9KKR8	VC_A1033	PF01547	sugar		
Q9KVX8	VC_0010	PF00497	amino acid (glutamine/glutamate/aspartate?)		
H9L4S6	VC_0776	PF01497	iron-hydroxamate		
Q9KS90	VC_1369	PF09084	nitrate/sulfonate/taurine		
Q9KQB9	VC_2081	PF01297	zinc		
Q9KRH8	VC_1664	PF13416	spermidine/putrescine		
Q9KSI3	VC_1273	PF03480	C4-dicarboxylate		
Q9KLN3	VC_A0710	PF00532	trimethylamine N-oxide		
Q9KRN3	VC_1603	PF00497	Unclassified		
Q9KKJ4	VC_A1113	PF13416	spermidine/putrescine		
Q9KLD9	VC_A0807	PF12727	phosphate		
Q9KLZ8	VC_A0591	PF00496	oligopeptide		
Q9KSD3	VC_1325	PF13407	xylose		
Q9KMU2	VC_A0227	PF01497	iron-hydroxamate	Enterobactin	DOI: 10.1016/j.febslet.2012.03.043
Q9KSC4	VC_1334	PF03401	tricarboxylate		
Q9KKW9	VC_A0981	PF00497	amino acid (glutamine/glutamate/aspartate?)		
Q9KRW1	VC_1523	PF12849	tungsten		
Q9KL05	VC_A0945	PF13416	sugar (maltose?)		
Q9KKX2	VC_A0978	PF00497	amino acid (glutamine/glutamate/aspartate?)		
Q9KU09	VC_0721	PF12849	phosphate		
Q9KNV8	VC_2622	PF00497	amino acid (glutamine/glutamate/aspartate?)		
Q9KVH5	VC_0171	PF00496	dipeptide/oligopeptide		

Q9KLI5	VC_A0759	PF00497	amino acid (glutamine/glutamate/aspartate?)		
Q9KL36	VC_A0913	PF01497	iron-hydroxamate		
Q9KRN8	VC_1598	PF00497	amino acid (glutamine/glutamate/aspartate?)		
Q9KUM7	VC_0488	PF03480	C4-dicarboxylate		
Q9KRV6	VC_1528	PF00497	Unclassified		
Q9KQY4	VC_1863	PF00497	amino acid (glutamine/glutamate/aspartate?)		
***Streptococcus pneumoniae* R6**					
Number of ORFs in the proteome: 2030					
Number of SBPs: 28 (1.38% of total)					
Lifestyle Summary: HUMAN/ANIMAL PATHOGEN					
**SeqID**	**Gene Locus**	**Pfam Code**	**Ligand prediction by TransportDB**	**Experimentally identified ligands**	**References/pdb accession codes**
P0A4G3	spr1494	PF01297	zinc		
Q8DPB1	spr1257	PF12849	phosphate		
Q8DN64	spr1895	PF12849	phosphate		
P0A4G1	spr1382	PF00496	oligopeptide		
P59214	spr1918	PF13416	sugar (maltose?)		
Q8DMZ7	spr2003	PF09084	nitrate/sulfonate/taurine		
Q8DQC2	spr0747	PF02608	sugar		
Q8DQ09	spr0906	PF01297	zinc	Zinc	DOI: 10.1016/j.jmb.2008.05.068; PDB: 3CX3
Q8DP50	spr1353	PF00497	amino acid (glutamine/glutamate/aspartate?)		
Q8DRI6	spr0101	PF00497	amino acid (glutamine/glutamate/aspartate?)		
Q8DRG2	spr0146	PF00497	amino acid (glutamine/glutamate/aspartate?)	Glutathione	PDB: 4EQ9
Q8DQL3	spr0620	PF00497	amino acid (glutamine/glutamate/aspartate?)		

Q8DPF2	spr1194	PF00496	dipeptide/oligopeptide		
Q8DNH8	spr1712	PF13416	sugar		
Q8CWT0	spr0545	PF00497	amino acid (glutamine/glutamate/aspartate?)		
Q8DPC5	spr1243	PF13416	spermidine/putrescine		
Q8DPY6	spr0934	PF01497	iron-hydroxamate	Bis, tris- propane	PDB: 4H59
Q8DNJ2	spr1687	PF01497	iron-hydroxamate		
Q8CWT1	spr0534	PF00497	amino acid (glutamine/glutamate/aspartate?)		
Q8DNI1	spr1707	PF00496	oligopeptide		
Q8DNU2	spr1534	PF01547	sugar		
Q8DPW4	spr0975	PF04392	Unclassified		
Q8DNU8	spr1527	PF13416	sugar		
Q8DQI1	spr0659	PF13458	leucine/valine		
Q8DRG1	spr0147	PF03180	methionine		
Q8DNN7	spr1620	PF13416	sugar		
Q8DR61	spr0327	PF00496	oligopeptide		
Q8DPB7	spr1251	PF00497	amino acid (glutamine/glutamate/aspartate?)		
***Legionella pneumophila* subsp. *pneumophila* str. Philadelphia-1**					
Number of ORFs in the proteome: 2930					
Number of SBPs: 15 (0.51% of total)					
Lifestyle Summary: HUMAN/ANIMAL PATHOGEN					
**SeqID**	**Gene Locus**	**Pfam Code**	**Ligand prediction by TransportDB**	**Experimentally identified ligands**	**References/pdb accession codes**
Q5ZR88	lpg2995	PF04348	leucine/valine		
Q5ZZH3	lpg0037	PF00497	amino acid (glutamine/glutamate/aspartate?)		
Q5ZY81	lpg0491	PF00497	Unclassified		
Q5ZWG0	lpg1125	PF00497	amino acid (glutamine/glutamate/aspartate?)		
Q5ZWX5	lpg0959	PF00496	oligopeptide		

Q5ZZC3	lpg0088	PF00497	amino acid (glutamine/glutamate/aspartate?)		
Q5ZWG2	lpg1123	PF00497	amino acid (glutamine/glutamate/aspartate?)		
Q5ZT89	lpg2273	PF13416	sugar		
Q5ZUG3	lpg1835	PF03180	methionine		
Q5ZXQ3	lpg0678	PF00497	amino acid (glutamine/glutamate/aspartate?)		
Q5ZV75	lpg1565	PF09084	nitrate/sulfonate/taurine		
Q5ZWE7	lpg1138	PF13416	spermidine/putrescine		
Q5ZY74	lpg0498	PF13458	leucine/valine		
Q5ZV85	lpg1555	PF00497	amino acid (glutamine/glutamate/aspartate?)		
Q5ZZ27	lpg0184	PF13407	ribose		
***Escherichia coli* MG1655**					
Number of ORFs in the proteome: 4391					
Number of SBPs: 49 (1.12% of total)					
Lifestyle Summary: HUMAN INTESTINAL MICROFLORA					
**SeqID**	**Gene Locus**	**Pfam Code**	**Ligand prediction by TransportDB**	**Experimentally identified ligands**	**References/pdb accession codes**
P76223	b1754	PF13416	spermidine/putrescine		
P75853	b0936	PF09084	nitrate/sulfonate/taurine		
P38683	b0994	PF00532	trimethylamine N-oxide		
P76042	b1310	PF01547	sugar		
P37676	b3579	PF03480	C4-dicarboxylate	2,3-diketo-l-gulonate (2,3-DKG)	DOI: 10.1099/mic.0.28334-0
P39172	b1857	PF01297	zinc ion	Zinc	DOI: 10.1046/j.1365-2958.1998.00883.x; PDB: 2OGW, 2OSV, 2PRS, 2PS0, 2PS3, 2PS9,

P0AFK9	b1123	PF13416	spermidine/putrescine	Polyamine (Spermidine / Putrescine)	DOI: 10.1002/pro.5560051004; DOI: 10.1074/jbc.271.16.9519; DOI: 10.1016/0014-5793(94)00435-8; PMID: 1939142; PDB: 1POT, 1POY
Q47537	b0365	PF04069	glycine betaine	Taurine, 2-Aminoethylphosphonic acid, N-(2-Acetamido)-2-aminoethanesulfonic acid, 2-(N-Morpholino)ethanesulfonic acid (MES)	DOI: 10.1128/jb.178.18.5438-5446.1996; PDB: 6SSY, 6ST0, 6ST1, 6STL
P39325	b4227	PF13407	ribose	Galactose	DOI: 10.1074/jbc.M109.054296
DOI: 10.107[illegible]	b3453	PF13416	sugar	Maltose, Glycerol-3-Phosphate	DOI: 10.1111/j.1365-2958.1988.tb00088.x
Q47622	b1294	PF00496	oligopeptide		
DOI: 10.111[illegible]	b3566	PF13407	xylose	Xylose	PMID: 8581399
P0AEM9	b1920	PF00497	amino acid (glutamine/glutamate/aspartate?)	Cystine / Cysteine	DOI: 10.1016/0024-3205(93)90103-a; PMID: 4564569
P33913	b2177	PF00496	oligopeptide		
P77269	b2548	PF13407	ribose		
DOI: 10.101[illegible]	b3020	PF00496	oligopeptide	Deoxycholate	DOI: 10.1111/1462-2920.12749
PMID: 8581[illegible]	b1243	PF00496	oligopeptide	Tripeptide (Gly-Leu-125I-Tyr)	PMID: 2187863; PDB: 3TCF, 3TCG
P04816	b3458	PF13458	leucine/valine	Leucine, Isoleucine, Valine, Threonine, Alanine	PMID: 3891753; 10.1002/jcb.240290305; 10.1016/0022-2836(89)90532-9
P77348	b1329	PF00496	oligopeptide	Murein tripeptide; Pro-Phe-Lys	DOI: 10.1128/JB.180.5.1215-1223.1998

PMID: 3891[illegible]	b3544	PF00496	dipeptide/oligopeptide	Dipeptide; Pro-Gly; Gly-Leu	DOI: 10.1128/jb.173.1.234-244.1991; DOI: 10.1111/j.1365-2958.1991.tb01876.x; DOI: 10.1002/pro.5560041110; DOI: 10.1021/bi00051a006; PDB: 1DPP,
P45766	JW3236	PF00497	amino acid (glutamine/glutamate/aspartate?)		
P33590	b3476	PF00496	nickel	Nickel, nickel butane-1,2,4-tricarboxylate, Fe-L1, Fe-L2, Ru(bpza)CO,	DOI: 10.1111/j.1365-2958.1993.tb01247.x; DOI: 10.1074/jbc.M307941200; PDB: 1UIV, 3DP8, 3E3K, 5ON0, 5ON1, 5ON4, 5ON5, 5ON8, 5ON9, 6R4Q
P76108	b1440	PF13416	spermidine/putrescine	dsDNA	DOI: 10.1016/j.bbrc.2016.01.137;
P33362	b2131	PF04069	glycine betaine	Choline-O-sulfate, glycine betaine, and dimethylsulfoniopropionate	DOI: 10.1021/acs.biochem.5b00274
P0AEU0	b2309	PF00497	amino acid (glutamine/glutamate/aspartate?)	Histidine	DOI: 10.1021/bi00182a004
DOI: 10.102[illegible]	b0197	PF03180	methionine	Methionine	DOI: 10.1128/JB.184.17.4930-4932.2002; PDB: 4YAH,
DOI: 10.100[illegible]	b0811	PF00497	amino acid (glutamine/glutamate/aspartate?)	Glutamine	DOI: 10.1007/BF00430437; DOI: 10.1006/jmbi.1998.1675

P0AEX9	b4034	PF01547	sugar (maltose?)	Maltose, maltotriose, maltodextrin, cyclodextrin	DOI: 10.1111/j.1432-1033.1976.tb10383.x; PMID: 2155217; DOI: 10.2210/pdb1mbp/pdb; DOI: 10.1021/bi00159a003; DOI: 10.1016/s0969-2126(97)00253-0; PDB: 1A7L, 1ANF, 1DMB
P0AD96	b3460	PF13458	leucine/valine	Leucine, Isoleucine, Valine	pdb: 1Z15, 1Z16, 1Z17, 1Z18; PMID: 3891753; 10.1002/jcb.240290305; 10.1016/0022-2836(89)90532-9; DOI: 10.1016/0014-5793(77)80331-1; DOI: 10.1016/0022-2836(89)90531-7
P45464	JW3116	PF04348	Not annotated in TransportDB	Penicillin	
P04846	b3661	PF03180	methionine		
P31133	b0854	PF13416	spermidine/putrescine	Polyamine, putrescine	pdb: 1A99, 4JDF; PMID: 8416922; DOI: 10.1007/s00726-013-1517-x; DOI: 10.1074/jbc.273.28.17604
P0AG82	b3728	PF12849	phosphate	Phosphate	pdb: 1A40, 1A40, 1A54, 1A55, 1IXG, 1IXH, 1IXI, 1OIB, 1PBP, 1QUI, 1QUJ, 1QUK, 1QUL, 2ABH, DOI: 10.1038/347402a0; DOI: 10.1038/nsb0797-519; DOI: 10.1021/bi980428z
P0AFM2	b2679	PF04069	glycine betaine	Glycine-betaine, proline-betaine	pdb: 1R9L, 1R9Q; PMID: 3305496; DOI: 10.1007/BF00290728; DOI: 10.3109/09687688.2012.754060; DOI: 10.1074/jbc.M309771200

P09551	b2310	PF00497	amino acid (glutamine/glutamate/aspartate?)		
P76128	b1487	PF00496	dipeptide/oligopeptide		
P30859	b0863	PF00497	amino acid (glutamine/glutamate/aspartate?)	Arginine	DOI: 10.1111/j.1365-2958.1995.mmi_17040675.x
P30860	b0860	PF00497	amino acid (glutamine/glutamate/aspartate?)		
P76142	b1516	PF13407	rhamnose	AI-2	DOI: 10.1128/JB.187.1.238-248.2005; DOI: 10.1128/JB.187.6.2066-2076.2005
P0AEL6	b0592	PF01497	iron-hydroxamate	Ferric enterobactin	pdb: 2M6K, 2M6L; DOI: 10.1099/13500872-141-7-1647
P15028	b4290	PF01497	iron-hydroxamate	Fe(3+) dicitrate	DOI: 10.1128/jb.171.5.2626-2633.1989
P39265	b4088	PF13407	ribose	D-Allose	pdb: 1GUB, 1GUD, 1RPJ; DOI: 10.1128/jb.179.24.7631-7637.1997; DOI: 10.1006/jmbi.1999.2571
P07822	b0152	PF01497	iron-hydroxamate	Gallichrome, hydroxamate-type siderophores, albomycin	pdb: 1EFD, 1ESZ, 1K2V, 1K7S; DOI: 10.1038/74048; DOI: 10.1074/jbc.M109385200; DOI: 10.1007/BF02456611
P02924	b1901	PF00532	ribose	L-arabinose, galactose	pdb: 1ABE, 1ABF, 1APB, 1BAP, 2WRZ, 5ABP, 6ABP, 7ABP, 8ABP, 9ABP; DOI: 10.1016/0022-2836(87)90607-3; PMID: 6885805; PMID: 326784; DOI: 10.1016/S0022-2836(05)80163-9; PMID: 7031057; PMID: 326785; PMID: 2204627
P37902	b0655	PF00497	amino acid (glutamine/glutamate/aspartate?)	Glutamate, aspartate	PMID: 1091635; PMID: 1091636

P75797	b0830	PF00496	dipeptide/oligopeptide	Glutathione	DOI: 10.1128/JB.187.17.5861-5867.2005; DOI: 10.1155/2018/3429569
P0AEE5	b2150	PF13407	xylose	Sugars, galactose	pdb: 1GLG, 2FVY, 2FW0, 2GBP, 2HPH, 2IPL. 2IPM, 2IPN, 2QW1; DOI: 10.1007/BF00267469; DOI: 10.1007/BF00330450; PMID: 7012152; DOI: 10.1073/pnas.80.7.1792; DOI: 10.1038/327635a0; DOI: 10.1126/science.3057628
P37028	b0158	PF01497	iron-hydroxamate	Cobalamin (Vit. B12)	pdb: 1N2Z, 1N4A, 1N4D, 2QI9, 4DBL, 4FI3, 5M29, 5M2Q, 5M34, 5M3B, 5OVW; DOI: 10.1128/JB.184.3.706-717.2002; DOI: 10.1073/pnas.262659699; DOI: 10.1074/jbc.M212239200
P02925	b3751	PF13407	ribose	Ribose	pdb: 1BA2, 1DBP, 1DRJ, 1DRK, 1URP, 2DRI, 2GX6; PMID: 6313683; PMID: 3011793; PMID: 4608146; DOI: 10.1128/jb.158.2.674-682.1984; 25533465; DOI: 10.1016/0022-2836(92)91033-l; PMID: 7982928; 9641984
***Agrobacterium fabrum* str. C58**					
Number of ORFs in the proteome: 5344					
Number of SBPs: 169 (3.16% of total)					
Lifestyle Summary: PLANT PATHOGEN					
**SeqID**	**Gene Locus**	**Pfam Code**	**Ligand prediction by TransportDB**		
Q8UB32	Atu3185	PF13416	sugar		

P35120	Atu6027	PF00497	amino acid (glutamine/glutamate/aspartate?)	Octopine, nopaline, pyronopaline, histopine, octopinic acid, noroctopinic acid,	pdb: 4P0I, 4POW, 4POX, 4PP0, 5ITO, 5ITP, 5OT8, 5OT9, 5OTA, 5OTC, 5OVZ; DOI: 10.1128/jb.174.3.841-849.1992
P25548	Atu2348	PF13407	xylose	Arabinose, galactose, galacturonate, D-xylose, D-fucose	pdb: 3URM, 3UUG, DOI: 10.1128/jb.172.11.6442-6446.1990; PMID: 8253785
A9CH17	Atu4727	PF03401	tricarboxylate		
Q7CTI9	Atu3881	PF13407	rhamnose		
Q7CYI8	Atu1789	PF02608	sugar		
A9CF36	Atu3372	PF13407	xylose		
Q7CVZ2	Atu4826	PF03480	C4-dicarboxylate		
Q7CRM8	Atu3114	PF13416	sugar		
A9CLC1	Atu5343	PF00496	dipeptide/oligopeptide		
Q7CVL1	Atu4687	PF00497	amino acid (glutamine/glutamate/aspartate?)		
A9CL62	Atu5459	PF01547	sugar		
Q7CZ25	Atu1521	PF01297	zinc ion		
A9CFT2	Atu3891	PF13416	sugar		
A9CKI0	Atu0249	PF03480	C4-dicarboxylate		
A9CEX5	Atu3253	PF03480	C4-dicarboxylate		
A9CLG5	Atu5268	PF03480	C4-dicarboxylate		
A9CES0	Atu3158	PF03480	C4-dicarboxylate		
A9CF33	Atu3368	PF03480	C4-dicarboxylate		
A9CET7	Atu3178	PF01297	manganese/zinc ion		
Q7CRP0	Atu3130	PF01547	sugar		
A9CKR7	Atu0063	PF13407	ribose		
Q7CV16	Atu4471	PF01297	manganese/zinc ion		
Q7D447	Atu5006	PF00497	amino acid (glutamine/glutamate/aspartate?)	Glucopine, deoxy-fructosyl-glutamine	pdb: 5L9O, 5LOM, , DOI: 10.1074/jbc.M116.745562
Q7D2V5	Atu5522	PF13458	branched-chain amino acid		
A9CLR3	Atu5058	PF13416	sugar		
A9CGS7	Atu4525	PF00496	dipeptide/oligopeptide		

A9CF11	Atu3338	PF01547	sugar		
A9CLD8	Atu5312	PF01497	iron-hydroxamate		
A9CEV8	Atu3222	PF13407	ribose		
A9CKB6	Atu0394	PF13416	sugar		
A9CEN1	Atu3088	PF13416	spermidine/putrescine		
A9CGV5	Atu4596	PF09084	phosphonates		
A9CK51	Atu0609	PF13416	spermidine/putrescine		
Q7CRP6	Atu3137	PF03480	C4-dicarboxylate		
A9CHW9	Atu2391	PF09084	phosphonates		
A9CL48	Atu5482	PF00497	amino acid (glutamine/glutamate/aspartate?)		
Q7CS92	Atu3357	PF00497	amino acid (glutamine/glutamate/aspartate?)		
A9CKE7	Atu0305	PF13416	sugar		
A9CLG7	Atu5265	PF13416	sugar		
A9CH15	Atu4719	PF00496	dipeptide/oligopeptide		
A9CLH3	Atu5253	PF00496	dipeptide/oligopeptide		
A9CGA5	Atu4243	PF13416	spermidine/putrescine	GABA	pdb: 4EQ7, 4EUO, DOI: 10.1111/mmi.12043
A9CEL8	Atu3063	PF13407	rhamnose		
A9CL91	Atu5403	PF13416	sugar		
A9CGI0	Atu4361	PF01547	sugar	Maltotriose, maltose, sucrose	pdb: 4QRZ, 4QSC, 4QSD
Q7CWQ0	Atu2587	PF00496	dipeptide/oligopeptide		
A9CF22	Atu3352	PF01547	sugar		
A9CGY7	Atu4654	PF13416	sugar		
Q7CV59	Atu4519	PF13458	urea		
A9CLH5	Atu5249	PF13416	spermidine/putrescine		
A9CHR1	Atu2505	PF01547	sugar		
A9CF60	Atu3409	PF00496	dipeptide/oligopeptide		
Q7CTQ1	Atu3957	PF04069	glycine betaine		
A9CEU5	Atu3198	PF13407	xylose		
Q7CTE3	Atu3821	PF13407	ribose		

A9CEW7	Atu3239	PF13416	sugar		
Q7CZC4	Atu1399	PF00497	amino acid (glutamine/glutamate/aspartate?)		
Q7CS07	Atu3260	PF03480	C4-dicarboxylate		
A9CI34	Atu2276	PF13458	leucine/valine		
A9CER5	Atu3151	PF13416	sugar		
A9CGU1	Atu4568	PF13416	spermidine/putrescine		
Q7D356	Atu5411	PF13416	2-aminoethylphosphonate		
Q7D260	Atu0116	PF03401	tricarboxylate		
A9CKN9	Atu0126	PF02608	sugar		
A9CGT6	Atu4552	PF13407	xylose		
Q7CSG3	Atu3433	PF00496	dipeptide/oligopeptide		
A9CF88	Atu3455	PF00496	dipeptide/oligopeptide		
A9CKY0	Atu6073	PF00496	dipeptide/oligopeptide		
Q7CU99	Atu4192	PF00496	dipeptide/oligopeptide		
A9CKK7	Atu0199	PF04069	glycine betaine		
A9CH32	Atu4755	PF00497	amino acid (glutamine/glutamate/aspartate?)		
A9CHT0	Atu2469	PF03401	tricarboxylate		
A9CLC6	Atu5330	PF03401	tricarboxylate		
Q7D348	Atu5420	PF13416	spermidine/putrescine		
Q7CX03	Atu2460	PF01497	iron-hydroxamate		
Q7CSE8	Atu3415	PF00497	amino acid (glutamine/glutamate/aspartate?)		
A9CHF0	Atu2744	PF03480	C4-dicarboxylate		
A9CFA6	Atu3487	PF13407	rhamnose		
Q7CSG2	Atu3432	PF09084	phosphonates		
Q7CS16	Atu3269	PF00496	dipeptide/oligopeptide		
Q7CVF6	Atu4626	PF00496	dipeptide/oligopeptide		
A9CFZ8	Atu4046	PF04069	glycine betaine		
A9CKL4	Atu0187	PF00496	oligopeptide		
Q7CVI9	Atu4661	PF00496	dipeptide/oligopeptide		
A9CGB3	Atu4252	PF01547	sugar		

Q7D230	Atu0157	PF09084	nitrate/sulfonate/taurine		
A9CJS1	Atu0895	PF04069	glycine betaine		
Q7CSP9	Atu3533	PF13407	rhamnose		
A9CLJ7	Atu5220	PF04069	glycine betaine		
A9CGQ9	Atu4489	PF03180	methionine		
A9CEN9	Atu3102	PF01547	sugar		
A9CF80	Atu3444	PF00496	dipeptide/oligopeptide		
Q7CV87	Atu4551	PF13407	xylose		
A9CET3	Atu3174	PF01547	sugar		
A9CHX9	Atu2365	PF00497	amino acid (glutamine/glutamate/aspartate?)		
A9CGF3	Atu4320	PF13407	xylose		
Q7CUZ5	Atu4447	PF01547	sugar		
Q7CRS2	Atu3165	PF01547	sugar		
A9CF37	Atu3375	PF00496	dipeptide/oligopeptide		
A9CJ45	Atu1404	PF01547	sugar		
Q7CWZ6	Atu2467	PF13416	2-aminoethylphosphonate		
A9CKY3	Atu6070	PF13407	xylose		
A9CHP9	Atu2518	PF00496	dipeptide/oligopeptide		
A9CEK8	Atu3041	PF00496	dipeptide/oligopeptide		
A9CIN5	Atu1774	PF00496	oligopeptide		
Q8U4Z3	Atu4233	PF00497	amino acid (glutamine/glutamate/aspartate?)		
A9CKZ1	Atu6059	PF09084	nitrate/sulfonate/taurine		
Q7CRM0	Atu3106	PF13416	sugar		
Q7CZR9	Atu1202	PF00496	dipeptide/oligopeptide		
Q7CXZ0	Atu2060	PF04069	choline		
A9CGZ5	Atu4678	PF00497	amino acid (glutamine/glutamate/aspartate?)		
A9CII3	Atu1879	PF00497	amino acid (glutamine/glutamate/aspartate?)		
A9CG31	Atu4113	PF00496	dipeptide/oligopeptide		

A9CIW8	Atu1577	PF00497	amino acid (glutamine/glutamate/aspartate?)		
A9CGD4	Atu4284	PF00497	amino acid (glutamine/glutamate/aspartate?)		
A9CFV8	Atu3967	PF13416	sugar		
A9CLI2	Atu5237	PF00497	amino acid (glutamine/glutamate/aspartate?)		
Q7CVH6	Atu4647	PF04069	choline		
Q7CUS0	Atu4369	PF13407	rhamnose		
Q7CV99	Atu4564	PF13416	sugar		
Q7CYA2	Atu1899	PF13407	xylose		
A9CF49	Atu3391	PF01497	iron-hydroxamate		
A9CF48	Atu3390	PF01497	iron-hydroxamate		
Q7CW05	Atu4842	PF13407	rhamnose		
Q7CV72	Atu4535	PF00497	amino acid (glutamine/glutamate/aspartate?)		
A9CLQ4	Atu5071	PF00496	dipeptide/oligopeptide		
A9CEN5	Atu3096	PF13416	sugar		
Q7CT32	Atu3688	PF01497	iron-hydroxamate		
A9CJ39	Atu1413	PF13458	leucine/valine		
A9CG80	Atu4208	PF01547	sugar (maltose?)		
A9CL69	Atu5441	PF00496	dipeptide/oligopeptide		
A9CFE7	Atu3576	PF13407	xylose		
Q7CWI2	Atu2672	PF04392	Unclassified		
A9CGM8	Atu4431	PF00496	dipeptide/oligopeptide		
A9CK59	Atu0591	PF01547	sugar		
A9CL88	Atu5409	PF00497	amino acid (glutamine/glutamate/aspartate?)		
Q7CRG5	Atu3049	PF00496	dipeptide/oligopeptide		
A9CJJ9	Atu1046	PF09084	nitrate/sulfonate/taurine		
Q7CW67	Atu2817	PF13407	rhamnose		
A9CKA7	Atu0420	PF12849	phosphate		

Q7D1E1	Atu0479	PF00497	amino acid (glutamine/glutamate/aspartate?)		
A9CFZ3	Atu4033	PF13407	rhamnose		
Q7CVL9	Atu4695	PF00496	dipeptide/oligopeptide		
A9CH25	Atu4744	PF13407	rhamnose		
Q7CTC2	Atu3800	PF04069	glycine betaine		
A9CF65	Atu3416	PF00497	amino acid (glutamine/glutamate/aspartate?)		
A9CEY9	Atu3282	PF01547	sugar		
A9CG38	Atu4123	PF13458	urea		
Q7CS34	Atu3287	PF09084	phosphonates		
Q7CX36	Atu2422	PF13458	leucine/valine	Val, Pro, Ala, GABA	pdb: 3IP5, 3IP6, 3IP7, 3IP9, 3IPA, 3IPC, DOI: 10.1074/jbc.M110.140715
A9CHS7	Atu2473	PF01497	iron-hydroxamate		
A9CEJ8	Atu3024	PF01547	sugar		
A9CI54	Atu2229	PF00497	amino acid (glutamine/glutamate/aspartate?)		
A9CHL4	Atu2601	PF13416	sugar		
A9CEX0	Atu3245	PF00497	amino acid (glutamine/glutamate/aspartate?)		
A9CGN3	Atu4438	PF00496	dipeptide/oligopeptide		
A9CJ57	Atu1388	PF00497	amino acid (glutamine/glutamate/aspartate?)		
A9CF79	Atu3442	PF00496	dipeptide/oligopeptide		
A9CGB6	Atu4259	PF00496	oligopeptide		
A9CGT1	Atu4534	PF00497	amino acid (glutamine/glutamate/aspartate?)		
A9CEX1	Atu3246	PF00497	amino acid (glutamine/glutamate/aspartate?)		
Q7CTW0	Atu4023	PF01497	iron-hydroxamate		
Q7CXG0	Atu2281	PF04069	choline		
A9CLM6	Atu5129	PF13416	spermidine/putrescine		

A9CF53	Atu3396	PF01497	cobalamin		
A9CGM0	Atu4421	PF13416	2-aminoethylphosphonate		
Q7CYE9	Atu1838	PF13458	leucine/valine		
Q52012	Atu6139	PF00496	dipeptide/oligopeptide	Agrocinopine A, agrocin 84, D-Glucose-2-phosphate, agrocinopine-3'-O-benzoate, L-arabinose-2-phosphate, L-arabinose-2-isopropylphosphate, 2-glucose-2-O-lactic acid phosphate	pdb: 4RA1, 4ZE8, 4ZE9, 4ZEB, 4ZEC, 4ZED, 4ZEI, 4ZEK, 6I7W, DOI: 10.1128/jb.179.23.7559-7572.1997; DOI: 10.1371/journal.ppat.1005071
***Xanthomonas campestris* pv. *campestris* (strain ATCC 33913 / DSM 3586 / NCPPB 528 / LMG 568 / P 25)**					
Number of ORFs in the proteome: 4127					
Number of SBPs: 9 (0.22% of total)					
Lifestyle Summary: PLANT PATHOGEN					
**SeqID**	**Gene Locus**	**Pfam Code**	**Ligand prediction by TransportDB**	**Experimentally identified ligands**	**References/pdb accession codes**
Q8P511	XCC3537	PF00497	amino acid (glutamine/glutamate/aspartate?)		
Q8PAF7	XCC1527	PF12849	phosphate		
Q8PAF6	XCC1528	PF12849	phosphate		
Q8P899	XCC2344	PF13416	spermidine/putrescine		
Q8PCG0	XCC0774	PF09084	nitrate/sulfonate/taurine		
Q8P8N4	XCC2206	PF13416	sugar		
Q8P4S9	XCC3628	PF03180	methionine		
Q8PCL3	XCC0711	PF04348	Not annotated in TransportDB		
Q8PE37	XCC0146	PF03480	C4-dicarboxylate		
***Salmonella typhimurium* (strain LT2 / SGSC1412 / ATCC 700720)**					
Number of ORFs in the proteome: 4533					
Number of SBPs: 45 (0.99% of total)					

Lifestyle Summary: HUMAN/ANIMAL PATHOGEN					
**SeqID**	**Gene Locus**	**Pfam Code**	**Ligand prediction by TransportDB**	**Experimentally identified ligands**	**References/pdb accession codes**
P43020	STM4054	PF03480	C4-dicarboxylate		
Q8ZML1	STM2811	PF04069	glycine betaine		
Q7CPK0	STM3557	PF13416	sugar		
P36634	STM1692	PF00496	oligopeptide	Antimicrobial peptides	DOI: 10.1002/j.1460-2075.1993.tb06089.x
P96062	STM0429	PF01547	2-aminoethylphosphonate	Phosphonates	DOI: 10.1128/jb.177.22.6411-6421.1995
P0A1W6	STM3564	PF13458	leucine/valine	Branched chain amino acids	DOI: 10.1093/oxfordjournals.jbchem.a123026
P19576	STM4229	PF01547	sugar (maltose?)	maltose, sugars, acarbose	pdb: 3JYR, 6L0Z, 6L3E, DOI: 10.1007/BF00331269; DOI: 10.1016/0167-4781(92)90492-i
P0A2C7	STM1222	PF13416	spermidine/putrescine		
Q8ZKQ1	STM4077	PF13407	rhamnose	Autoinducer-2, (2R,4S)-2-methyl-2,3,3,4-tetrahydroxytetrahydrofuran (R-THMF),	pdb: 1TJY, 1TM2, DOI: 10.1046/j.1365-2958.2001.02669.x
P17215	STM3567	PF13458	leucine/valine	Branched chain amino acids	DOI: 10.1093/oxfordjournals.jbchem.a123026
P02910	STM2354	PF00497	amino acid (glutamine/glutamate/aspartate?)	Histidine	pdb: 1HPB; DOI: 10.1038/298723a0; DOI: 10.1073/pnas.78.10.6038; PMID: 7007375; DOI: 10.1073/pnas.95.7.3495; DOI: 10.1074/jbc.274.2.739; PMID: 8307974

Q8ZPK2	STM1493	PF04069	glycine betaine	Glycine betaine, choline-O-sulfate	DOI: 10.1128/JB.00495-12
P0A2C5	STM3884	PF13407	ribose	Ribose	PMID: 6415058
P06202	STM1746	PF00496	oligopeptide	Oligopeptides	pdb: 1B05, 1B0H, 1B1H, 1B2H, 1B32, 1B3F, 1B3G, 1B3H, 1B3L, 1B40, 1B46, 1B4H, 1B4Z, 1B51, 1B52, 1B58, 1B5H, 1B5I, 1B5J, 1B6H, 1B7H, 1B9J, 1JET, 1JEU, 1JEV, 1OLA, 1OLC, 1QKA, 1QKB, 1RKM, 2OLB, 2RKM, DOI: 10.1016/0022-2836(87)90332-9; DOI: 10.1126/science.8202710; DOI: 10.1016/s0969-2126(01)00276-3; DOI: 10.1110/ps.8.7.1432; https://doi.org/10.1006/jmbi.1999.2929
Q9ZF60	STM0665	PF00497	amino acid (glutamine/glutamate/aspartate?)		
P02911	STM2355	PF00497	amino acid (glutamine/glutamate/aspartate?)	Arginine, positively charge amino acids	pdb: 1LAF, 1LAG, 1LAH, 1LST, 2LAO, 5OWF, 6FT2, 6MKU, 6MKW, 6MKX, 6ML0, 6ML9, 6MLA, 6MLD, 6MLE, 6MLG, 6MLI, 6MLJ, 6MLN, 6MLO, 6MLP, 6MLV, DOI: 10.1073/pnas.78.10.6038; DOI: 10.1016/j.bbamem.2013.08.024; PMID: 1748660; PMID: 8496186
Q8ZQM3	STM0849	PF00496	dipeptide/oligopeptide		
Q8ZRP7	STM0206	PF01497	iron-hydroxamate	Cobalamin	DOI: 10.1128/JB.181.17.5539-5541.1999

P23905	STM2190	PF13407	xylose	Glucose; galactose; (2R)-glyceryl-beta-D-galactopyranoside	doi: 10.1006/jmbi.1993.1549; doi: 10.1111/j.1742-4658.2009.06945.x; PMID: 1967096; DOI: 10.1007/BF00330498; DOI: 10.2210/pdb1gbp/pdb; DOI: 10.1006/jmbi.1993.1549; PMID: 8132630
Q8ZRN1	STM0245	PF03180	methionine		
Q8ZLA9	STM3630	PF00496	dipeptide/oligopeptide		
Q8ZPA3	STM1633	PF00497	amino acid (glutamine/glutamate/aspartate?)	D-Alanine	PDB: 4F3S;
Q8ZM02	STM3169	PF03480	C4-dicarboxylate		
Q8ZNM8	STM2165	PF04069	glycine betaine		
Q8ZKX5	STM3857	PF12849	phosphate		
Q8XG72	STM0890	PF00497	amino acid (glutamine/glutamate/aspartate?)		
Q8ZM19	STM3142	PF01497	iron-hydroxamate		
Q8ZNK0	STM2216	PF00496	oligopeptide		
Q8ZP68	STM1679	PF00496	oligopeptide		
Q9FA46	STM2786	PF03401	tricarboxylate		
Q8ZLH2	STM3528	PF12849	phosphate		
Q7CQV2	STM0887	PF00497	amino acid (glutamine/glutamate/aspartate?)		
Q8ZNV8	STM1891	PF01297	zinc ion	Zinc	doi: 10.1016/j.jmb.2011.04.036; DOI: 10.1016/j.jmb.2011.04.036
Q8ZR34	STM0594	PF01497	iron-hydroxamate		
Q9S4Z1	STM0510	PF03180	methionine		
Q8ZRQ0	STM0193	PF01497	iron-hydroxamate		
Q8ZLU4	STM3264	PF04348	Not annotated in TransportDB		
Q8ZKZ4	STM3825	PF00532	trimethylamine N-oxide		
Q8ZQK0	STM0877	PF13416	spermidine/putrescine		

H9L419	STM1954	PF00497	amino acid (glutamine/glutamate/aspartate?)		
Q7CPX8	STM2861	PF01297	manganese/zinc ion		
Q7CQW0	STM0830	PF00497	amino acid (glutamine/glutamate/aspartate?)		
Q8ZL79	STM3673	PF03480	C4-dicarboxylate		
Q8ZKA9	STM4351	PF00497	amino acid (glutamine/glutamate/aspartate?)	Arginine	doi: 10.1002/prot.23060
Q8ZPY3	STM1255	PF00496	dipeptide/oligopeptide		
***Bacillus amyloliquefaciens* FZB42**					
Number of ORFs in the proteome: 3711					
Number of SBPs: 33 (0.89% of total)					
Lifestyle Summary: SOIL					
**SeqID**	**Gene Locus**	**Pfam Code**	**Ligand prediction by TransportDB**	**Experimentally identified ligands**	**References/pdb accession codes**
A7Z1K0	RBAM_004770	PF03480	C4-dicarboxylate		
A7Z3C6	RBAM_011380	PF00496	dipeptide/oligopeptide		
A7Z8W3	RBAM_031080	PF04069	glycine betaine		
A7Z7G3	RBAM_025810	PF01547	sugar		
A7Z0U0	RBAM_002120	PF01497	iron-hydroxamate		
A7Z147	RBAM_003240	PF04069	glycine betaine		
A7Z2Q1	RBAM_009120	PF09084	phosphonates		
A7Z2D8	RBAM_007800	PF03401	tricarboxylate		
A7Z6R3	RBAM_023290	PF12849	phosphate		
A7Z7Z3	RBAM_027610	PF09084	nitrate/sulfonate/taurine		
A7ZAF9	RBAM_036560	PF01497	iron-hydroxamate		
A7Z3R8	RBAM_012810	PF00496	oligopeptide		
A7Z869	RBAM_028640	PF02608	sugar		
A7Z8T8	RBAM_030830	PF00497	amino acid (glutamine/glutamate/aspartate?)		
A7Z8I6	RBAM_029810	PF03180	methionine		

A7Z1A1	RBAM_003780	PF00497	amino acid (glutamine/glutamate/aspartate?)		
A7Z9G7	RBAM_033130	PF13407	xylose		
A7Z2S6	RBAM_009380	PF03180	methionine		
A7Z1B5	RBAM_003920	PF00497	amino acid (glutamine/glutamate/aspartate?)		
A7Z134	RBAM_003110	PF01297	manganese/zinc ion		
A7Z393	RBAM_011050	PF02608	sugar		
A7Z3D1	RBAM_011430	PF00496	oligopeptide		
A7Z1D1	RBAM_004080	PF01497	iron-hydroxamate		
A7Z339	RBAM_010510	PF01497	iron-hydroxamate		
A7Z8H1	RBAM_029660	PF01547	sugar		
A7Z738	RBAM_024540	PF00497	amino acid (glutamine/glutamate/aspartate?)		
A7Z7V1	RBAM_027190	PF01547	sugar		
A7ZA87	RBAM_035840	PF01497	iron-hydroxamate		
A7Z8V8	RBAM_031030	PF04069	glycine betaine		
A7Z8P9	RBAM_030440	PF01497	iron-hydroxamate		
A7Z3B8	RBAM_011300	PF02608	sugar		
A7Z6G3	RBAM_022280	PF00497	amino acid (glutamine/glutamate/aspartate?)		
A7Z8N6	RBAM_030310	PF01497	iron-hydroxamate		
***Synechocystis* sp. PCC 6803**					
Number of ORFs in the proteome: 3507					
Number of SBPs: 27 (0.77% of total)					
Lifestyle Summary: FRESH/MARINE WATERS					
**SeqID**	**Gene Locus**	**Pfam Code**	**Ligand prediction by TransportDB**	**Experimentally identified ligands**	**References/pdb accession codes**
Q55200	sll0679	PF12849	phosphate		
Q55471	slr0529	PF01547	Unclassified	Glucosylglycerol, sucrose, trehalose	DOI: 10.1007/s002030000201

P72827	slr1295	PF13416	thiamin	Iron (II) and iron (III)	doi: 10.1074/jbc.M704136200; DOI: 10.1128/JB.183.9.2779-2784.2001; DOI: 10.1093/pcp/pce106; DOI: 10.1099/00221287-148-10-3293;
Q55146	sll0064	PF00497	amino acid (glutamine/glutamate/aspartate?)		
P72610	fecB	PF01497	Gene locus not annotated in Uniprot		
Q55199	pstS	PF12849	Gene locus not annotated in Uniprot		
Q55633	slr0341	PF00497	amino acid (glutamine/glutamate/aspartate?)		
P73732	slr1740	PF00496	dipeptide/oligopeptide		
P73325	srrA	PF01547	Gene locus not annotated in Uniprot		
P73615	slr1865	PF13416	sugar		
P74223	glnH	PF00497	Gene locus not annotated in Uniprot		
P73785	pstS	PF12849	Gene locus not annotated in Uniprot		
P73085	zntC	PF01297	Gene locus not annotated in Uniprot	Zinc	doi: 10.1016/j.jmb.2003.09.008; doi: 10.1021/bi700763w
P72611	fecB	PF01497	Gene locus not annotated in Uniprot		
P72593	fecB	PF01497	Gene locus not annotated in Uniprot		
P73693	sll1699	PF00496	dipeptide/oligopeptide		
P72598	sll1202	PF01497	iron-hydroxamate		
P72707	sll0224	PF00497	amino acid (glutamine/glutamate/aspartate?)		
Q55723	sll0606	PF13458	leucine/valine		
Q55556	sll0174	PF00497	amino acid (glutamine/glutamate/aspartate?)		
P72750	sll1037	PF16868	phosphonates		
Q55387	natB	PF01094	Gene locus not annotated in Uniprot		
P73589	dctP	PF03480	Gene locus not annotated in Uniprot		
Q79EF9	mntC	PF01297	Gene locus not annotated in Uniprot	Manganese	doi: 10.1016/j.jmb.2005.03.006; doi: 10.1107/S174430911300153X

Q55410	sll0540	PF12849	phosphate		
P74100	slr1962	PF03480	C4-dicarboxylate		
P73643	sll1762	PF00497	amino acid (glutamine/glutamate/aspartate?)		
***Ralstonia solanacearum* GMI1000**					
Number of ORFs in the proteome: 5002					
Number of SBPs: 67 (1.34% of total)					
Lifestyle Summary: PLANT PATHOGEN					
**SeqID**	**Gene Locus**	**Pfam Code**	**Ligand prediction by TransportDB**	**Experimentally identified ligands**	**References/pdb accession codes**
Q8XU51	RSc3342	PF13458	urea		
Q8XY77	RSc1886	PF00496	dipeptide/oligopeptide		
Q8Y255	RSc0481	PF00497	amino acid (glutamine/glutamate/aspartate?)		
Q8XQC9	RSp1298	PF13458	urea		
Q8Y071	RSc1173	PF00496	oligopeptide		
Q8XPR3	RSp1575	PF00497	amino acid (glutamine/glutamate/aspartate?)		
Q8XYM5	RSc1733	PF13416	spermidine/putrescine		
Q8Y0M6	RSc1017	PF13407	ribose		
Q8XV97	RSc2934	PF13416	iron(III)		
Q8XU03	RSc3391	PF09084	nitrate/sulfonate/taurine		
Q8XXL0	RSc2103	PF00497	amino acid (glutamine/glutamate/aspartate?)		
Q8XX72	RSc2244	PF13458	urea		
Q8Y0S0	RSc0973	PF00497	amino acid (glutamine/glutamate/aspartate?)		
Q8XWN2	RSc2441	PF13458	leucine/valine		
Q8XXM0	RSc2093	PF12849	2-aminoethylphosphonate		
Q8XPP6	RSp1592	PF13458	leucine/valine		
Q8XX55	RSc2261	PF00497	amino acid (glutamine/glutamate/aspartate?)		

Q8XY84	RSc1879	PF13458	leucine/valine		
Q8XSD7	RSp0539	PF13458	leucine/valine		
Q8XXE8	RSc2166	PF03480	C4-dicarboxylate		
Q8XQS9	RSp1140	PF00497	amino acid (glutamine/glutamate/aspartate?)		
Q8Y2Q2	RSc0283	PF01497	iron-hydroxamate		
Q8XVG7	RSc2864	PF13458	leucine/valine		
Q8XQ39	RSp1447	PF13458	leucine/valine		
Q8XUP0	RSc3147	PF13458	leucine/valine		
Q8XUA6	RSc3285	PF13458	urea		
Q8XUU5	RSc3088	PF03401	tricarboxylate		
Q8XTU1	RSp0013	PF13458	leucine/valine		
Q8XV68	RSc2963	PF03401	tricarboxylate		
Q8XQA3	RSp1383	PF09084	nitrate/sulfonate/taurine		
Q8XXP3	RSc2070	PF13458	urea		
Q8XV94	RSc2937	PF13416	iron(III)		
Q8Y0X1	RSc0922	PF03180	methionine		
Q8XQB6	RSp1370	PF05048	Not annotated in TransportDB		
Q8XWG2	RSc2512	PF00497	amino acid (glutamine/glutamate/aspartate?)		
Q8XTY5	RSc3410	PF00497	amino acid (glutamine/glutamate/aspartate?)		
Q8XU64	RSc3329	PF13458	leucine/valine		
Q8XZL5	RSc1380	PF00496	dipeptide/oligopeptide		
Q8XSJ3	RSp0481	PF00497	amino acid (glutamine/glutamate/aspartate?)		
Q8XWP5	RSc2428	PF13458	leucine/valine		
Q8XPK7	RSp1633	PF13407	xylose		
Q8XXD8	RSc2177	PF02608	sugar		
Q8XU93	RSc3300	PF13458	leucine/valine		
Q8XZY1	RSc1264	PF13416	sugar		
Q8Y396	RSc0084	PF00497	amino acid (glutamine/glutamate/aspartate?)		

Q8XYK8	RSc1750	PF13458	urea		
Q8XVG1	RSc2870	PF00496	dipeptide/oligopeptide		
Q8XUY2	RSc3051	PF01547	sugar		
Q8XQ56	RSp1430	PF13458	leucine/valine		
Q8Y3D6	RSc0044	PF00496	dipeptide/oligopeptide		
Q8XTC7	RSp0186	PF00497	amino acid (glutamine/glutamate/aspartate?)		
Q8XTP2	RSp0064	PF04069	glycine betaine		
Q8XR42	RSp1026	PF13407	rhamnose		
Q8XZ75	RSc1529	PF12849	phosphate		
Q8XU83	RSc3310	PF12849	phosphate		
Q8XXH0	RSc2144	PF01547	sugar		
Q8XUX2	RSc3061	PF13458	leucine/valine	Leucine	PDB: 3LOP
Q8XYL8	RSc1740	PF04392	Unclassified		
Q8XWS6	RSc2394	PF01497	iron-hydroxamate		
Q8Y359	RSc0122	PF00497	amino acid (glutamine/glutamate/aspartate?)		
Q8XWG4	RSc2510	PF03480	C4-dicarboxylate		
Q8XRC7	RSp0931	PF00497	amino acid (glutamine/glutamate/aspartate?)		
Q8XX59	RSc2257	PF00497	amino acid (glutamine/glutamate/aspartate?)		
Q8Y004	RSc1241	PF13407	xylose		
Q8XU08	RSc3386	PF03180	methionine		
Q8XXV7	RSc2006	PF00496	dipeptide/oligopeptide		
Q8XVS1	RSc2758	PF00532	ribose		
***Bacillus subtilis* subsp. *subtilis* 168**					
Number of ORFs in the proteome: 4260					
Number of SBPs: 40 (0.94% of total)					
Lifestyle Summary: SOIL					
**SeqID**	**Gene Locus**	**Pfam Code**	**Ligand prediction by TransportDB**	**Experimentally identified ligands**	**References/pdb accession codes**

O34805	BSU33180	PF01497	iron-hydroxamate		
P42199	BSU03610	PF00497	amino acid (glutamine/glutamate/aspartate?)	Cysteine	DOI: 10.1128/JB.186.15.4875-4884.2004
C0SP94	BSU10330	PF01497	iron-hydroxamate	Siderofores	https://doi.org/10.1046/j.1365-2958.2002.03113.x
P54941	BSU39610	PF01497	iron-hydroxamate	Siderofores	DOI: 10.1128/JB.188.10.3664-3673.2006
P54952	BSU39500	PF00497	amino acid (glutamine/glutamate/aspartate?)	Cysteine	DOI: 10.1128/JB.186.15.4875-4884.2004
P54594	BSU09110	PF03180	methionine	Copper	DOI: 10.1099/13500872-142-11-3021
Q795R2	BSU30160	PF13416	sugar		
O32156	BSU32600	PF01547	sugar		
P40400	BSU08840	PF09084	glycine betaine	Alkanesulfonates, taurine, isethionate, sulfoacetate	DOI: 10.1099/00221287-144-9-2555
O34439	BSU07600	PF03401	tricarboxylate		
P94421	BSU03830	PF01497	iron-hydroxamate	Siderophore (petrobactin)	DOI: 10.1073/pnas.0904793106
O31518	BSU06970	PF13416	sugar	2,6-anhydro-3-deoxy-L-threo-hex-2-enonic acid-(1-2)-[beta-D-galactopyranose-(1-4)]alpha-L-rhamnopyranose (trisacharide)	PDB: 5Z6B; DOI: 10.1128/AEM.00147-07
O34335	BSU30270	PF01547	sugar	Sugars of raffinose family	DOI: 10.1128/JB.00109-19
O34852	BSU29370	PF00497	amino acid (glutamine/glutamate/aspartate?)	Cysteine	DOI: 10.1128/JB.186.15.4875-4884.2004
P42400	BSU03380	PF00497	amino acid (glutamine/glutamate/aspartate?)		

O31567	BSU08440	PF01497	iron-hydroxamate	Siderofores	https://doi.org/10.1046/j.1365-2958.2002.03113.x
O34966	BSU02850	PF01297	manganese/zinc ion	Manganese, zinc	PDB: 2O1E; DOI: 10.1128/JB.180.22.5815-5821.1998; DOI: 10.1128/JB.184.23.6508-6514.2002; DOI: 10.1093/jb/mvr098
O34348	BSU07520	PF01497	iron-hydroxamate	Sidorofore	DOI: 10.1128/JB.188.10.3664-3673.2006
O06989	BSU34610	PF13416	sugar (maltose?)	Maltose	DOI: 10.1128/JB.00213-06
P37580	BSU33320	PF01497	iron-hydroxamate	Sidorofore, iron-hydroxamate	DOI: 10.1128/JB.188.10.3664-3673.2006; DOI: 10.1111/j.1365-2958.1993.tb01208.x
O32243	BSU33810	PF04069	glycine betaine	Carnitine, glycine betaine, choline, ectoine and arsenobetaine	doi: 10.1042/BJ20102097; doi: 10.1016/j.jmb.2011.05.037; doi: 10.1111/1462-2920.13999; DOI: 10.1046/j.1365-2958.1999.01354.x; DOI: 10.1128/jb.178.17.5071-5079.1996; DOI: 10.1128/jb.178.17.5071-5079.1996
O05252	BSU31540	PF02608	sugar	Guanosine	DOI: 10.1128/JB.05899-11
Q45462	BSU33710	PF04069	glycine betaine	Glycine betaine, carnitine, choline	PDB: 6EYG, 6EYL, 6EYQ; DOI: 10.1046/j.1365-2958.1999.01354.x; DOI: 10.1128/jb.177.23.6874-6880.1995; DOI: 10.1128/JB.06642-11

P24141	BSU11430	PF00496	oligopeptide	Oligopeptides	DOI: 10.1111/j.1365-2958.1991.tb01838.x; DOI: 10.1128/jb.173.4.1388-1398.1991
O34385	BSU30770	PF01297	manganese/zinc ion	Manganese	DOI: 10.1046/j.1365-2958.2000.01811.x; DOI: 10.1046/j.1365-2958.2003.03648.x
P37966	BSU07100	PF01547	sugar		
P46922	BSU03000	PF04069	glycine betaine	Glycine betaine, proline betaine, sulfobetaine dimethylsulfonioacetate, arsenobetaine	doi: 10.1016/j.jmb.2005.12.085; doi: 10.1128/JB.00346-08; doi: 10.1111/1462-2920.13999; DOI: 10.1074/jbc.270.28.16701
O32167	BSU32730	PF03180	methionine	Methionine	DOI: 10.1016/j.resmic.2003.11.008
O34406	BSU29380	PF00497	amino acid (glutamine/glutamate/aspartate?)	Cysteine	
P54535	BSU23980	PF00497	amino acid (glutamine/glutamate/aspartate?)	Arginine	DOI: 10.1186/gb-2001-2-6-research0019
P40409	BSU01630	PF01497	iron-hydroxamate	Ferri-Bacillibactin, Ferri-Enterobactin, triscatecholate siderophore mimic mecam	doi: 10.1002/anie.200902495; doi: 10.1016/j.chembiol.2011.05.006; DOI: 10.1111/j.1365-2958.2007.05905.x
P42061	BSU11381/BSU11382	PF00496	dipeptide/oligopeptide	Nonapeptide VDSKNTSSW	doi: 10.1016/j.jmb.2004.10.089; DOI: 10.1111/j.1365-2958.1994.tb00436.x
P26906	BSU12960	PF00496	oligopeptide		
O32436	BSU11300	PF02608	sugar		
P96600	BSU04440	PF03480	C4-dicarboxylate	Fumarate and succinate	doi: 10.1099/00221287-146-2-263
O34563	BSU27440	PF00497	amino acid (glutamine/glutamate/aspartate?)		

P94528	BSU28750	PF01547	sugar	Arabinobiose, arabinotriose, arabinotetraose, maltoriose, maltotetraose, maltopentaose, debranched arabinan	doi: 10.1128/JB.00832-10
O07009	BSU34160	PF13416	Unclassified	maltoheptaose, α-cyclodextrin, β-cyclodextrin, γ-cyclodextrin	doi: 10.1111/j.1574-6968.2001.tb10862.x
P36949	BSU35960	PF13407	xylose	Ribose	doi: 10.1099/13500872-140-8-1829
P46338	BSU24990	PF12849	phosphate	Phosphate	doi: 10.1128/jb.179.8.2534-2539.1997
***Geobacillus thermodenitrificans* NG80-2**					
Number of ORFs in the proteome: 3424					
Number of SBPs: 51 (1.49% of total)					
Lifestyle Summary: OIL RESERVOIR					
**SeqID**	**Gene Locus**	**Pfam Code**	**Ligand prediction by TransportDB**	**Experimentally identified ligands**	**References/pdb accession codes**
A4IQM7	GTNG_2282	PF00497	amino acid (glutamine/glutamate/aspartate?)		
A4IPU7	GTNG_1999	PF00496	dipeptide/oligopeptide		
A4ISL8	GTNG_2977	PF16868	phosphonates		
A4IN11	GTNG_1345	PF03180	methionine		
A4IKK6	GTNG_0478	PF00496	dipeptide/oligopeptide		
A4ITB6	GTNG_3227	PF13416	2-aminoethylphosphonate		
A4ISJ0	GTNG_2949	PF03180	methionine		
A4IL04	GTNG_0626	PF13416	spermidine/putrescine		
A4IJQ4	GTNG_0174	PF01497	iron-hydroxamate		
A4IP83	GTNG_1773	PF01547	sugar		

A4IT20	GTNG_3129	PF13407	rhamnose		
A4IPA7	GTNG_1801	PF13407	xylose		
A4IRZ5	GTNG_2754	PF09084	nitrate/sulfonate/taurine		
A4IT97	GTNG_3206	PF13416	sugar		
A4IPL6	GTNG_1915	PF04392	Unclassified		
A4IS05	GTNG_2764	PF01297	manganese/zinc ion		
A4IPC9	GTNG_1823	PF13416	sugar		
A4IMF7	GTNG_1137	PF02608	sugar		
A4IKD2	GTNG_0404	PF13416	sugar		
A4IMY3	GTNG_1317	PF01497	iron-hydroxamate		
A4IPB0	GTNG_1804	PF13407	rhamnose		
A4ITD1	GTNG_3244	PF13416	sugar		
A4IPE9	GTNG_1843	PF03401	tricarboxylate		
A4IT17	GTNG_3126	PF01547	sugar		
A4ISH1	GTNG_2930	PF13458	leucine/valine		
A4IKY9	GTNG_0611	PF13416	sugar (maltose?)		
A4IQY8	GTNG_2397	PF12849	phosphate		
A4IPB9	GTNG_1813	PF13407	ribose		
A4ITR8	GTNG_3385	PF00497	amino acid (glutamine/glutamate/aspartate?)		
A4IPY4	GTNG_2036	PF09084	phosphonates		
A4ISM5	GTNG_2984	PF13458	leucine/valine		
A4ITG8	GTNG_3283	PF00496	dipeptide/oligopeptide		
A4ISE1	GTNG_2900	PF01497	iron-hydroxamate		
A4IQE4	GTNG_2199	PF01497	iron-hydroxamate		
A4IKV4	GTNG_0576	PF04392	Unclassified		
A4IRK2	GTNG_2611	PF09084	glycine betaine		
A4IPA0	GTNG_1794	PF01547	sugar		
A4IK07	GTNG_0277	PF04069	glycine betaine		
A4IPX0	GTNG_2022	PF01547	sugar		
A4IP90	GTNG_1780	PF01547	sugar		
A4IP42	GTNG_1732	PF05048	Not annotated in TransportDB		
A4IMU1	GTNG_1275	PF01497	iron-hydroxamate		

A4IL67	GTNG_0691	PF00496	oligopeptide		
A4IT61	GTNG_3170	PF13407	xylose		
A4ILL0	GTNG_0836	PF13407	rhamnose		
A4INH9	GTNG_1513	PF13458	leucine/valine		
A4ILJ8	GTNG_0824	PF01547	sugar		
A4IPP7	GTNG_1946	PF03480	C4-dicarboxylate		
A4IPH8	GTNG_1874	PF03480	C4-dicarboxylate		
A4IPH6	GTNG_1870	PF03480	C4-dicarboxylate		
A4IKN1	GTNG_0503	PF03480	C4-dicarboxylate		
***Mycobacterium tuberculosis* H37Rv**					
Number of ORFs in the proteome: 3993					
Number of SBPs: 17 (0.43% of total)					
Lifestyle Summary: HUMAN/ANIMAL PATHOGEN					
**SeqID**	**Gene Locus**	**Pfam Code**	**Ligand prediction by TransportDB**	**Experimentally identified ligands**	**References/pdb accession codes**
P9WL77	Rv2585c	PF00496	dipeptide/oligopeptide		
P9WGU5	Rv1280c	PF00496	dipeptide/oligopeptide		
P9WGT7	Rv0928	PF12849	phosphate	Phosphate	doi: 10.1002/prot.24548; doi: 10.1016/j.tube.2010.09.004
P9WGU1	Rv0934	PF12849	phosphate	Phosphate	doi: 10.1128/IAI.73.3.1898-1902.2005; doi: 10.1016/s0969-2126(03)00109-6
P9WGU7	Rv1166	PF00496	dipeptide/oligopeptide		
P9WGT9	Rv0932c	PF12849	phosphate	Phosphate	doi: 10.1128/IAI.73.3.1898-1902.2005
P9WGU9	Rv1235	PF01547	sugar	Trehalose	doi: 10.1073/pnas.1014642108
P96257	Rv0411c	PF00497	amino acid (glutamine/glutamate/aspartate?)	Aspartate, glutamate, asparagine	doi: 10.1128/mBio.00931-18
O53291	Rv3044	PF01497	iron-hydroxamate		
O53485	Rv2041c	PF01547	sugar		

P71619	Rv2833c	PF13416	sugar	Glycerophosphocholine, glycerophosphoserine, glycerophosphoethanolamine, glycerophosphoinositol, glycerophosphoinositol-4-phosphate	doi: 10.1021/acschembio.9b00204
O07257	Rv2059	PF01297	manganese/zinc ion		
P71894	Rv2318	PF01547	sugar	D-glucosamine, D-galactosamine, D-mannosamine, muramic acid, D-glucosamine-6-phosphate	doi: 10.1098/rsob.160105
L7N6B2	Rv0265c	PF01497	iron-hydroxamate		
O69725	Rv3759c	PF04069	glycine betaine		
O50459	Rv1244	PF04069	glycine betaine		
I6X811	Rv3666c	PF00496	oligopeptide	Tetrapeptide (Ser-Ser-Val-Thr), tetrapeptide (Val-Val-Val-Val), heme and hemoglobin	doi: 10.1038/s41467-019-12109-5
**Borrelia burgdORFseri B31**					
Number of ORFs in the proteome: 1290					
Number of SBPs: 12 (0.93% of total)					
Lifestyle Summary: HUMAN/ANIMAL PATHOGEN					
**SeqID**	**Gene Locus**	**Pfam Code**	**Ligand prediction by TransportDB**	**Experimentally identified ligands**	**References/pdb accession codes**
O51233	BB_0215	PF12849	phosphate	Phosphate	doi: 10.1002/pro.2406
P0CL55	BB_0385	PF02608	sugar		
Q45010	BB_0383	PF02608	sugar		
Q45011	BB_0382	PF02608	sugar		
P0CL65	BB_0384	PF02608	sugar		
O51298	BB_0319	PF02608	sugar		

O51308	BB_0330	PF00496	oligopeptide		
O51584	BB_0639	PF13416	spermidine/putrescine		
H7C7K8	BB_B16	PF00496	oligopeptide	Peptide of unknown sequence	PDB: 4GL8
O51169	BB_0144	PF04069	glycine betaine	Glycine-betaine	PDB: 3TMG
O51307	BB_0328	PF00496	oligopeptide		
O50927	BB_A34	PF00496	oligopeptide		
***Caulobacter crescentus* CB15**					
Number of ORFs in the proteome: 3720					
Number of SBPs: 9 (0.24% of total)					
Lifestyle: Isolated from a pond water in the USA. Free-living non-pathogenic environmental isolate					
Lifestyle Summary: FRESH/MARINE WATERS					
**SeqID**	**Gene Locus**	**Pfam Code**	**Ligand prediction by TransportDB**	**Experimentally identified ligands**	**References/pdb accession codes**
Q9A8A8	CC_1446	PF03480	C4-dicarboxylate		
Q9A3R5	CC_3137	PF13416	spermidine/putrescine		
Q9A536	CC_2633	PF09084	nitrate/sulfonate/taurine		
Q9A793	CC_1833	PF09084	nitrate/sulfonate/taurine		
Q9A8B3	CC_1441	PF00497	amino acid (glutamine/glutamate/aspartate?)		
Q9A904	CC_1191	PF01497	iron-hydroxamate		
Q9A507	CC_2664	PF03180	methionine		
Q9A849	CC_1515	PF12849	phosphate		
Q9A9V2	CC_0859	PF13407	xylose		
***Aliivibrio fischeri* MJ11**					
Number of ORFs in the proteome: 4034					
Number of SBPs: 39 (0.97% of total)					
Lifestyle Summary: ANIMAL SYMBIONT					
**SeqID**	**Gene Locus**	**Pfam Code**	**Ligand prediction by TransportDB**	**Experimentally identified ligands**	**References/pdb accession codes**
B5ESA5	VFMJ11_A0037	PF03480	C4-dicarboxylate		

B5EVA6	VFMJ11_A1076	PF13416	sugar (maltose?)		
B5FBG2	VFMJ11_0727	PF13407	ribose		
B5FCU6	VFMJ11_2644	PF00496	dipeptide/oligopeptide		
B5ET94	VFMJ11_A0357	PF09084	nitrate/sulfonate/taurine		
B5ET84	VFMJ11_A0347	PF00496	dipeptide/oligopeptide		
B5FEJ7	VFMJ11_1543	PF13407	xylose		
B5FET0	VFMJ11_1626	PF00497	amino acid (glutamine/glutamate/aspartate?)		
B5FGF3	VFMJ11_0262	PF16868	glycine betaine		
B5FA26	VFMJ11_2146	PF13416	sugar (maltose?)		
B5FBD5	VFMJ11_0700	PF00496	dipeptide/oligopeptide		
B5FE48	VFMJ11_1394	PF13416	spermidine/putrescine		
B5FBD8	VFMJ11_0703	PF03180	methionine		
B5EUT7	VFMJ11_A0907	PF01497	iron-hydroxamate		
B5FE49	VFMJ11_1395	PF13416	spermidine/putrescine		
B5FF16	VFMJ11_1697	PF00497	amino acid (glutamine/glutamate/aspartate?)		
B5FF39	VFMJ11_1720	PF12849	phosphate		
B5FE02	VFMJ11_1348	PF00497	amino acid (glutamine/glutamate/aspartate?)		
B5FBE5	VFMJ11_0710	PF00496	dipeptide/oligopeptide		
B5FF26	VFMJ11_1707	PF00496	oligopeptide		
B5ETU9	VFMJ11_A0568	PF12849	phosphate		
B5FF53	VFMJ11_1734	PF00532	trimethylamine N-oxide		
B5EUV6	VFMJ11_A0926	PF13416	sugar (maltose?)		
B5EUY4	VFMJ11_A0954	PF01497	iron-hydroxamate		
B5FDV8	VFMJ11_1302	PF01497	iron-hydroxamate		
B5FB45	VFMJ11_2323	PF04348	Not annotated in TransportDB		
B5FBV6	VFMJ11_2487	PF01297	zinc		
B5EST4	VFMJ11_A0195	PF01497	iron-hydroxamate		
B5FAM2	VFMJ11_2247	PF00496	dipeptide/oligopeptide		
B5FDH2	VFMJ11_1166	PF16868	phosphonates		
B5FA03	VFMJ11_2123	PF12849	phosphate		

B5FEV2	VFMJ11_0008	PF00497	amino acid (glutamine/glutamate/aspartate?)		
B5FBY7	VFMJ11_0822	PF04069	glycine betaine		
B5EVC5	VFMJ11_A1095	PF01497	iron-hydroxamate		
B5FEE0	VFMJ11_1486	PF12849	tungsten		
B5FG88	VFMJ11_1917	PF03401	tricarboxylate		
B5FC33	VFMJ11_0868	PF01297	zinc		
B5ESK9	VFMJ11_A0120	PF13416	spermidine/putrescine		
B5ETG7	VFMJ11_A0430	PF00497	amino acid (glutamine/glutamate/aspartate?)		
***Streptomyces coelicolor* A3(2)**					
Number of ORFs in the proteome: 8038					
Number of SBPs: 93 (1.16% of total)					
Lifestyle Summary: SOIL					
**SeqID**	**Gene Locus**	**Pfam Code**	**Ligand prediction by TransportDB**	**Experimentally identified ligands**	**References/pdb accession codes**
O50499	SCO6005	PF01547	N-acetylglucosamine	N,N'-diacetylchitobiose, N-acetylglucosamine	doi: 10.1264/jsme2.ME17172
Q9K491	SCO5232	PF01547	sugar	N-acetylglucosamine, N,N'-Diacetylchitobiose, N,N',N'''-triacetylchitotriose, N,N′,N‴,N‴-tetraacetylchitotetraose, N,N′,N‴,N‴,N′ ′ ′ ′-pentaacetylchitopentaose	doi: 10.1128/AEM.02612-06
O69944	SCO6569	PF13407	xylose		
Q9RK79	SCO0531	PF01547	sugar		
Q9RJA4	SCO0660	PF01547	sugar		
Q8CJP2	SCO6114	PF00496	dipeptide/oligopeptide		
O50503	SCO6009	PF13407	xylose		

Q7AKN1	SCO2933	PF04069	glycine betaine		
Q9L2K2	SCO0706	PF13458	urea		
Q9F3F1	SCO7606	PF13458	leucine/valine		
Q9L027	SCO7028	PF01547	sugar		
Q9EX42	SCO0996	PF01497	cobalamin		
Q9RJG1	SCO0473	PF01297	zinc		
O69956	SCO5780	PF16868	phosphonates		
Q9RKQ6	SCO2272	PF01497	iron-hydroxamate		
Q9K443	SCO1056	PF13416	sugar		
Q7AKD7	SCO7013	PF01547	sugar		
Q9RCZ6	SCO0914	PF01547	sugar		
Q9K3Y9	SCO1344	PF00496	dipeptide/oligopeptide		
Q9KZV9	SCO4142	PF12849	phosphate		
O86596	SCO6231	PF01547	sugar		
Q9ADH5	SCO6088	PF01547	sugar		
Q9F353	SCO5117	PF00496	oligopeptide		
Q9L057	SCO2978	PF01547	sugar		
Q9X9X4	SCO1898	PF01547	sugar		
Q9KXV5	SCO4286	PF13416	sugar		
Q9F3C0	SCO7555	PF13416	sugar		
O86690	SCO6644	PF00496	dipeptide/oligopeptide		
Q9L216	SCO6816	PF12849	phosphate		
Q9L263	SCO2664	PF00497	amino acid (glutamine/glutamate/aspartate?)		
Q7AKP1	SCO2231	PF01547	sugar (maltose?)		
Q9L1U8	SCO2946	PF13416	sugar		
Q9FBS5	SCO7167	PF13416	sugar		
O50494	SCO5776	PF00497	amino acid (glutamine/glutamate/aspartate?)		
Q9KYS9	SCO5686	PF01547	sugar		
Q9K434	SCO1065	PF01547	sugar		
Q9S1S7	SCO0065	PF01547	sugar		
Q9RKH6	SCO3456	PF13416	spermidine/putrescine		

Q93RU4	SCO1882	PF01547	sugar		
Q9X832	SCO6065	PF04069	glycine betaine		
Q9S2J5	SCO2008	PF13458	leucine/valine		
O86636	SCO5716	PF00496	dipeptide/oligopeptide		
Q9RJV8	SCO0352	PF01547	sugar		
O54107	SCO5911	PF00496	dipeptide/oligopeptide		
Q9KXY7	SCO2428	PF12849	phosphate		
Q93JA7	SCO7489	PF13416	sugar		
Q9K3U4	SCO4401	PF13458	leucine/valine		
Q9ZBG2	SCO6451	PF00496	dipeptide/oligopeptide		
O87856	SCO6601	PF01547	sugar		
Q8CK63	SCO0273	PF01547	sugar		
Q9AK41	SCO4885	PF02608	sugar		
Q9EWY8	SCO1138	PF03401	tricarboxylate		
Q9F3B2	SCO7563	PF00496	dipeptide/oligopeptide		
Q9RL34	SCO0453	PF01547	sugar		
O86741	SCO5658	PF13416	spermidine/putrescine		
Q9RJ53	SCO1655	PF00496	dipeptide/oligopeptide		
Q9RK12	SCO0494	PF01497	iron-hydroxamate		
Q9X861	SCO3505	PF01547	sugar		
Q9KYQ3	SCO3667	PF13407	xylose		
Q9L074	SCO2780	PF01497	iron-hydroxamate	Ferrioxamine B	doi: 10.1098/rstb.2017.0068
Q9K3I8	SCO4832	PF04069	glycine betaine		
Q9L1L4	SCO5430	PF01547	sugar		
Q9S2C3	SCO0290	PF01547	sugar		
Q9AK42	SCO4884	PF02608	sugar		
Q9L1C5	SCO1557	PF03180	methionine		
Q9RDN3	SCO2404	PF13407	xylose		
Q9FBZ7	SCO7185	PF13458	leucine/valine		
Q93IU2	SCO5113	PF00496	dipeptide/oligopeptide		
Q9F3I6	SCO2795	PF13416	sugar		
Q9RJU7	SCO0363	PF01547	sugar (maltose?)		

Q9RDC3	SCO2828	PF00497	amino acid (glutamine/glutamate/aspartate?)		
Q9L178	SCO7399	PF01497	iron-hydroxamate		
Q9L151	SCO7408	PF01547	sugar		
Q9EWQ0	SCO7677	PF00496	dipeptide/oligopeptide		
Q9RK72	SCO0538	PF01547	sugar		
Q9F3K5	SCO5260	PF00497	amino acid (glutamine/glutamate/aspartate?)		
Q9RD69	SCO0808	PF13407	rhamnose		
Q9RKF3	SCO3484	PF01547	sugar		
O86572	SCO5477	PF00496	dipeptide/oligopeptide		
Q93J94	SCO7503	PF01547	sugar		
O86832	SCO5667	PF13416	spermidine/putrescine		
Q8CJY9	SCO2658	PF13416	sugar		
Q9L0B2	SCO2434	PF01547	sugar		
Q9X837	SCO6070	PF13407	rhamnose		
Q9K467	SCO7218	PF01497	cobalamin		
Q9RL38	SCO0449	PF13407	rhamnose		
Q9RIV4	SCO0952	PF01547	sugar		
Q9L2H8	SCO2505	PF01297	zinc		
Q9L270	SCO1539	PF01547	sugar		
Q9RKT5	SCO6257	PF13407	ribose		
Q9KZH3	SCO6979	PF13407	rhamnose		
Q9KYY3	SCO7546	PF09084	phosphonates		
Q9L0Z4	SCO1465	PF01547	sugar		
***Sinorhizobium meliloti* 1021**					
Number of ORFs in the proteome: 6169					
Number of SBPs: 189 (3.06% of total)					
Lifestyle Summary: BENEFICIAL PLANT ASSOCIATED					
**SeqID**	**Gene Locus**	**Pfam Code**	**Ligand prediction by TransportDB**	**Experimentally identified ligands**	**References/pdb accession codes**
Q926H8	RB0402	PF13416	sugar		

Q92KZ7	R03301	PF01547	sugar		
Q9Z3R5	R00695	PF01547	sugar	Sucrose, maltose and trehalose	doi: 10.1128/JB.181.14.4176-4184.1999
Q9X4Y1	RB1567	PF00496	dipeptide/oligopeptide	Melibiose and raffinose	doi: 10.1128/JB.180.21.5739-5748.1998
Q92T97	SMc02589	PF13416	spermidine/putrescine		
Q931B1	SMa0067	PF13407	ribose		
Q92ZS4	SMa0709	PF01547	sugar		
Q92LQ8	SMc03121	PF13458	urea		
Q92XK3	SMa2305	PF01547	sugar		
Q926H2	SM_b21221	PF01547	sugar		
Q926G8	SM_b21215	PF00496	dipeptide/oligopeptide		
Q92YR5	SMa1462	PF04069	glycine betaine		
Q92PN7	SMc00271	PF03480	C4-dicarboxylate		
Q92XC3	SM_b20036	PF03480	C4-dicarboxylate		
Q92MV9	SMc01966	PF13416	spermidine/putrescine		
Q92MD0	SMc00676	PF00497	amino acid (glutamine/glutamate/aspartate?)		
Q930R1	SMa0252	PF03480	C4-dicarboxylate		
Q926E7	SM_b20706	PF00497	amino acid (glutamine/glutamate/aspartate?)		
Q926F1	SM_b21432	PF01497	iron-hydroxamate		
Q926E5	SM_b20720	PF13407	xylose		
Q92YY1	SMa1337	PF01547	sugar		
Q926H7	SM_b21016	PF13407	rhamnose	Autoinducer-2 (AI-2), (2R,4S)-2-methyl-2,3,3,4-tetrahydroxytetrahydrofuran (AI-2 isomer)	doi: 10.1111/j.1365-2958.2008.06477.x
Q92MN0	SMc02356	PF13458	leucine/valine		
Q92P64	SMc04259	PF01547	sugar		
Q92MS8	SMc02021	PF13407	ribose		
Q92WP7	SM_b20292	PF16868	phosphonate		
Q92YT4	SMa1427	PF13407	xylose		

Q930Q0	SMa0273	PF13407	xylose		
Q92VN0	SM_b21090	PF13416	sugar		
Q92WQ5	SM_b20284	PF13416	spermidine/putrescine		
Q92YV8	SMa1375	PF00496	dipeptide/oligopeptide		
Q926F7	SM_b20976	PF00497	amino acid (glutamine/glutamate/aspartate?)		
Q92WN0	SM_b20316	PF13407	rhamnose		
Q92P72	SMc04251	PF03480	C4-dicarboxylate		
Q92WH9	SM_b20374	PF03480	C4-dicarboxylate		
Q926F9	SM_b20931	PF13407	rhamnose		
Q92WP4	SM_b20295	PF03480	C4-dicarboxylate		
Q92M36	SMc04037	PF00496	dipeptide/oligopeptide		
Q926H0	SM_b21273	PF13416	2-aminoethylphosphonate		
Q930S9	SMa0218	PF13407	ribose		
Q92WB9	SM_b20442	PF03480	C4-dicarboxylate		
Q92MC6	SMc00672	PF04069	glycine betaine		
Q930W1	SMa0157	PF03480	C4-dicarboxylate		
Q92XT1	SMa2129	PF02608	sugar		
Q92TP4	SM_b20771	PF03401	tricarboxylate		
Q92V05	SM_b21353	PF03480	C4-dicarboxylate		
Q92PP3	SMc00265	PF03480	C4-dicarboxylate		
Q92WY4	SM_b20178	PF13458	leucine/valine		
Q92P42	SMc04293	PF00496	dipeptide/oligopeptide		
Q92S11	SMc02324	PF13407	rhamnose		
Q92N37	SMc02737	PF04069	choline	Choline and acetylcholine	doi: 10.1074/jbc.M806021200
Q926G0	SM_b21604	PF13416	sugar		
Q92WY7	SM_b20175	PF00497	amino acid (glutamine/glutamate/aspartate?)		
Q92T30	SMc02832	PF00496	oligopeptide		
Q92W79	SM_b20484	PF13407	rhamnose		
Q92LE5	SMc03269	PF00496	dipeptide/oligopeptide		
Q92L85	SMc03813	PF13407	rhamnose		

Q926F4	SM_b20895	PF13407	xylose		
Q926G7	SM_b21345	PF13407	ribose		
Q926B4	SM_b21438	PF03480	C4-dicarboxylate		
Q930T8	SMa0203	PF13407	rhamnose		
Q92XA6	SM_b20056	PF01497	cobalamin		
Q92Y64	SMa1860	PF00496	dipeptide/oligopeptide		
Q92W87	SM_b20476	PF00496	dipeptide/oligopeptide		
Q92PJ6	SMc00513	PF13458	leucine/valine		
Q92N15	SMc01512	PF01497	iron-hydroxamate		
Q92MS4	SMc02025	PF00496	dipeptide/oligopeptide		
Q92WS6	SM_b20263	PF00497	amino acid (glutamine/glutamate/aspartate?)		
Q926F8	SM_b20971	PF01547	sugar		
Q926G1	SM_b21595	PF01547	sugar		
Q92LI8	SMc02471	PF13416	sugar		
Q92P75	SMc04245	PF01297	zinc ion		
Q92W55	SM_b20508	PF00532	ribose		
Q92MP7	SMc02338	PF13407	xylose		
Q92MU8	SMc01977	PF13416	sugar		
Q92NV4	SMc04341	PF04069	glycine betaine		
Q7ANS2	SM_b20325	PF01547	sugar		
Q926H6	SM_b21037	PF00496	dipeptide/oligopeptide		
Q926I0	SM_b21652	PF13416	sugar		
Q926H1	SM_b21261	PF00496	dipeptide/oligopeptide		
Q92LX5	SMc03157	PF03180	methionine		
Q931A3	SMa0082	PF00497	amino acid (glutamine/glutamate/aspartate?)		
Q92N26	SMc01525	PF00496	dipeptide/oligopeptide		
Q92N02	SMc01496	PF01547	sugar		
Q92NE7	SMc01647	PF00496	dipeptide/oligopeptide		
Q92W59	SM_b20504	PF13407	rhamnose		
Q92RK9	SMc00991	PF13416	spermidine/putrescine		
Q92MM2	SMc02415	PF00496	dipeptide/oligopeptide		

Q92LR1	SMc03124	PF00496	dipeptide/oligopeptide		
Q926H5	SM_b21097	PF00497	amino acid (glutamine/glutamate/aspartate?)		
Q92YH7	SMa1651	PF00496	nickel		
Q92PY3	SMc01205	PF00497	amino acid (glutamine/glutamate/aspartate?)		
Q92S63	SMc02259	PF00497	amino acid (glutamine/glutamate/aspartate?)		
Q926E6	SM_b20712	PF13407	xylose		
Q92XV7	SMa2075	PF00496	dipeptide/oligopeptide		
Q92WK4	SM_b20349	PF13407	ribose		
Q926F6	SM_b20981	PF03480	C4-dicarboxylate		
Q926E1	SM_b20620	PF13407	rhamnose		
Q92SZ7	SMc02873	PF13416	sugar		
Q7D4K4	SMa1183	PF05048	Not annotated in TransportDB		
Q926F3	SM_b20902	PF13407	xylose		
Q926G6	SM_b21377	PF13407	ribose		
Q926H4	SM_b21103	PF01547	sugar		
Q926E0	SM_b20634	PF13416	sugar		
Q92WV7	SM_b20231	PF13416	sugar		
Q930Z3	SMa0104	PF00496	dipeptide/oligopeptide		
Q92X54	SM_b20108	PF00496	dipeptide/oligopeptide		
Q92RX4	SMc00770	PF13416	spermidine/putrescine		
Q92PT7	SMc00951	PF00496	oligopeptide		
Q92PA9	SMc00140	PF00497	amino acid (glutamine/glutamate/aspartate?)		
Q92MM0	SMc02417	PF00496	dipeptide/oligopeptide		
Q92PR4	SMc00242	PF02608	sugar		
Q92L69	SMc03830	PF04392	Unclassified		
Q92R76	SMc02378	PF04069	glycine betaine		
Q92TR9	SM_b20724	PF03401	tricarboxylate		
Q926F5	SM_b20856	PF13407	xylose		
Q926C5	SMc01946	PF13458	leucine/valine		

Q930N2	SMa0302	PF00496	dipeptide/oligopeptide		
Q92ZU1	SMa0677	PF00497	amino acid (glutamine/glutamate/aspartate?)		
Q92SC8	SMc02171	PF13407	ribose		
Q926F0	SM_b21461	PF13416	sugar		
Q92RW0	SMc00786	PF00496	dipeptide/oligopeptide		
Q92YD3	SMa1729	PF04069	glycine betaine		
Q926G9	SM_b21144	PF04069	glycine betaine		
Q92LR8	SMc03131	PF00497	amino acid (glutamine/glutamate/aspartate?)		
Q92V92	SM_b21151	PF13416	sugar		
Q7ANR0	SM_b21196	PF00496	oligopeptide		
Q92YS7	SMa1438	PF00496	dipeptide/oligopeptide		
Q92XZ2	SMa2000	PF13407	xylose		
Q92XQ7	SMa2199	PF00497	amino acid (glutamine/glutamate/aspartate?)		
Q92WY1	SM_b20181	PF09084	nitrate/sulfonate/taurine		
Q92WC8	SM_b20428	PF00497	amino acid (glutamine/glutamate/aspartate?)	Ectoine and hydroxyectoine	doi: 10.1016/j.jmb.2007.09.071
Q92X35	SM_b20127	PF02608	sugar		
Q92NE3	SMc01652	PF13416	spermidine/putrescine		
Q92X90	SM_b20072	PF13407	xylose		
Q92NF1	SMc01642	PF00496	dipeptide/oligopeptide		
Q92ZM2	SMa0799	PF13416	spermidine/putrescine		
Q92L16	SMc03891	PF00497	amino acid (glutamine/glutamate/aspartate?)		
Q92S93	SMc02219	PF00497	amino acid (glutamine/glutamate/aspartate?)		
Q92LL5	SMc02509	PF01297	manganese/zinc ion		
Q926G5	SM_b21526	PF09084	glycine betaine		
Q930D8	SMa0495	PF00497	amino acid (glutamine/glutamate/aspartate?)		
Q92T63	SMc04135	PF13416	sugar		

Q926F2	SM_b21421	PF13407	xylose		
Q92YB5	SMa1755	PF13416	spermidine/putrescine		
Q930D2	SMa0506	PF00497	amino acid (glutamine/glutamate/aspartate?)		
Q926E9	SM_b20660	PF13416	sugar		
Q92TN0	SM_b20568	PF13458	urea		
Q92X18	SM_b20144	PF00496	dipeptide/oligopeptide		
Q926G3	SM_b21572	PF04069	glycine betaine		
Q92XH5	SMa2361	PF02608	sugar		
Q92WH0	SM_b20383	PF13416	spermidine/putrescine		
Q92K04	SMc01827	PF09084	nitrate/sulfonate/taurine		
Q92ZH2	SMa0950	PF13416	spermidine/putrescine		
Q92ND7	SMc01659	PF01497	iron-hydroxamate		
Q926G2	SM_b21587	PF13407	ribose		
Q92YW6	SMa1364	PF13416	sugar		
Q926E4	SM_b20723	PF13416	2-aminoethylphosphonate		
Q92NG1	SMc01632	PF13416	spermidine/putrescine		
Q92ZZ6	SMa0576	PF13458	leucine/valine		
Q92YC2	SMa1746	PF01497	iron-hydroxamate		
Q92MR7	SMc02033	PF13407	xylose		
Q926E8	SM_b20671	PF13407	xylose		
Q92RF4	SMc00078	PF13458	leucine/valine		
Q92P29	SMc04311	PF04069	glycine betaine		
Q92TD7	SMc02774	PF13407	rhamnose		
Q92Q71	SMc02118	PF00497	amino acid (glutamine/glutamate/aspartate?)		
Q930F5	SMa0466	PF00496	dipeptide/oligopeptide		
Q92W28	SM_b20538	PF13416	sugar		
Q926H3	SM_b21135	PF00497	amino acid (glutamine/glutamate/aspartate?)		
Q92L38	SMc03864	PF00497	amino acid (glutamine/glutamate/aspartate?)		
Q92SA4	SMc02146	PF12849	phosphate		

Q92MP1	SMc02344	PF09084	phosphonates		
Q930I7	SMa0392	PF13416	spermidine/putrescine		
Q92XE1	SM_b20018	PF13407	xylose		
Q926D6	SMc02884	PF02608	sugar		
Q92LM0	SMc02514	PF01547	sugar		
Q92WE4	SM_b20410	PF04069	choline		
Q92YV6	SMa1379	PF00496	dipeptide/oligopeptide		
Q926E3	SM_b20570	PF09084	phosphonates		
Q92Y30	SMa1927	PF03401	tricarboxylate		
Q92XV1	SMa2087	PF09084	phosphonates		
Q92XU8	SMa2093	PF09084	nitrate/sulfonate/taurine		
Q92XD4	SM_b20025	PF03401	tricarboxylate		
Q92NG5	SMc01628	PF01547	sugar		
Q92VT4	SM_b21043	PF03401	tricarboxylate		
Q92WM6	SM_b20320	PF03480	C4-dicarboxylate		
Q92PI5	SMc00499	PF03401	tricarboxylate		
Q92NW2	SMc04289	PF03480	C4-dicarboxylate		
***Clostridium botulinum* A str. ATCC 3502**					
Number of ORFs in the proteome: 3590					
Number of SBPs: 35 (0.97% of total)					
Lifestyle Summary: HUMAN/ANIMAL PATHOGEN					
**SeqID**	**Gene Locus**	**Pfam Code**	**Ligand prediction by TransportDB**	**Experimentally identified ligands**	**References/pdb accession codes**
A5HYZ7	CBO0453	PF13458	leucine/valine		
A5HY04	CBO0107	PF13416	sugar		
A5I2E3	CBO1675	PF00496	dipeptide/oligopeptide		
A5I0S8	CBO1086	PF03180	methionine		
A5I1P3	CBO1411	PF00497	amino acid (glutamine/glutamate/aspartate?)		
A5I102	CBO1162	PF04392	Unclassified		
A5HYJ4	CBO0301	PF01497	iron-hydroxamate		

A5I3E2	CBO2018	PF00497	amino acid (glutamine/glutamate/aspartate?)		
A5I1H2	CBO1337	PF01497	iron-hydroxamate		
A5HYB2	CBO0219	PF02608	sugar		
A5I120	CBO1181	PF00497	amino acid (glutamine/glutamate/aspartate?)		
A5I0U9	CBO1107	PF13416	spermidine/putrescine		
A5HYS8	CBO0384	PF12849	phosphate		
A5HYZ0	CBO0446	PF00497	amino acid (glutamine/glutamate/aspartate?)		
A5I371	CBO1946	PF12849	tungsten		
A5HYJ9	CBO0306	PF01497	iron-hydroxamate		
A5HZL3	CBO0670	PF04392	trimethylamine N-oxide		
A5HZM9	CBO0686	PF01497	iron-hydroxamate		
A5I165	CBO1227	PF00496	dipeptide/oligopeptide		
A5I6Z1	CBO3264	PF00496	oligopeptide		
A5I0S9	CBO1087	PF03180	methionine		
A5HZW3	CBO0770	PF01547	sugar		
A5I6N0	CBO3150	PF00496	oligopeptide		
A5I1I9	CBO1355	PF00496	dipeptide/oligopeptide		
A5I1X9	CBO1501	PF03180	methionine		
A5I4V6	CBO2525	PF12849	phosphate		
A5I0G6	CBO0974	PF01497	cobalamin		
A5I1J7	CBO1363	PF01297	manganese/zinc ion		
A5I743	CBO3318	PF01297	manganese/zinc ion		
A5I1Z5	CBO1518	PF13416	sugar		
A5I5Y1	CBO2897	PF01497	iron-hydroxamate		
A5HYK3	CBO0310	PF01497	iron-hydroxamate		
A5I0P0	CBO1047	PF01497	iron-hydroxamate		
A5I0Z2	CBO1151	PF00497	amino acid (glutamine/glutamate/aspartate?)		
A5I6V0	CBO3221	PF01547	sugar		

***Lactococcus lactis* subsp. *lactis* Il1403**					
Number of ORFs in the proteome: 2225					
Number of SBPs: 20 (0.90% of total)					
Lifestyle Summary: FOOD					
**SeqID**	**Gene Locus**	**Pfam Code**	**Ligand prediction by TransportDB**	**Experimentally identified ligands**	**References/pdb accession codes**
Q9CFZ5	LL1316	PF01297	zinc		
Q9CEK0	LL1837	PF00496	dipeptide/oligopeptide		
Q9CIN8	L117444	PF03180	methionine		
Q9CI27	L124415	PF00496	oligopeptide		
Q9CIN5	L120334	PF03180	methionine		
Q9CEZ8	L128695	PF13416	sugar (maltose?)		
Q9CFM9	L61620	PF02608	sugar		
Q9CIN6	L119452	PF03180	methionine		
Q9CDU6	L167426	PF01297	manganese/zinc ion		
Q9CEW4	L158188	PF12849	phosphate		
Q9CIN7	L118475	PF03180	methionine		
Q9CIL3	L143312	PF00496	oligopeptide		
Q9CF46	L82310	PF13407	xylose		
Q9CI05	L158343	PF00497	amino acid (glutamine/glutamate/aspartate?)		
Q9CIM6	L129403	PF01497	iron-hydroxamate		
Q9CH05	L162009	PF00497	amino acid (glutamine/glutamate/aspartate?)		
Q9CDZ9	L121253	PF00497	amino acid (glutamine/glutamate/aspartate?)		
Q9CEW3	L159386	PF12849	phosphate		
Q9CIL2	L145267	PF00496	oligopeptide		
Q9CGD1	L178329	PF13416	spermidine/putrescine		
***Comamonas testosteroni* CNB-2**					
Number of ORFs in the proteome: 4780					
Number of SBPs: 177 (3.70% of total)					

Lifestyle Summary: ACTIVE SLUDGE					
**SeqID**	**Gene Locus**	**Pfam Code**	**Ligand prediction by TransportDB**	**Experimentally identified ligands**	**References/pdb accession codes**
D0IVL1	CtCNB1_2193	PF03401	tricarboxylate		
D0J7K0	CtCNB1_4186	PF03480	C4-dicarboxylate		
A0A454XZJ5	CtCNB1_4331	PF03401	tricarboxylate		
D0J432	CtCNB1_1479	PF03401	tricarboxylate		
D0J6W2	CtCNB1_1923	PF03401	tricarboxylate		
D0J453	CtCNB1_3521	PF03480	C4-dicarboxylate		
D0J4P2	CtCNB1_1554	PF03180	methionine		
D0IUT0	CtCNB1_4341	PF09084	nitrate/sulfonate/taurine		
D0IWG9	CtCNB1_0241	PF03401	tricarboxylate		
D0J634	CtCNB1_1779	PF03401	tricarboxylate		
D0J6J2	CtCNB1_3963	PF03401	tricarboxylate		
D0J776	CtCNB1_4062	PF03401	tricarboxylate		
D0J8C2	CtCNB1_4280	PF03401	tricarboxylate		
D0J6F2	CtCNB1_3923	PF03401	tricarboxylate		
D0J605	CtCNB1_3911	PF03401	tricarboxylate		
D0J021	CtCNB1_0742	PF03401	tricarboxylate		
D0J1H4	CtCNB1_3117	PF03401	tricarboxylate		
D0IWP8	CtCNB1_2311	PF03401	tricarboxylate		
D0J1E5	CtCNB1_3088	PF03401	tricarboxylate		
D0J4E7	CtCNB1_3615	PF03401	tricarboxylate		
D0IZ79	CtCNB1_0583	PF03401	tricarboxylate		
D0J2X2	CtCNB1_3349	PF03401	tricarboxylate		
D0J5E6	CtCNB1_1673	PF03401	tricarboxylate		
D0J3N5	CtCNB1_3483	PF13458	urea		
D0J0F8	CtCNB1_2883	PF03401	tricarboxylate		
D0IXD4	CtCNB1_4700	PF03401	tricarboxylate		
D0IX93	CtCNB1_4659	PF03401	tricarboxylate		
A0A454XMV	CtCNB1_0577	PF00497	amino acid (glutamine/glutamate/aspartate?)		
D0J7M0	CtCNB1_0014	PF03401	tricarboxylate		

A0A454XSE(	CtCNB1_0781	PF03401	tricarboxylate		
D0J614	CtCNB1_3920	PF03401	tricarboxylate		
D0J3U6	CtCNB1_1393	PF04069	glycine betaine		
D0IXM6	CtCNB1_0375	PF03401	tricarboxylate		
D0J1V0	CtCNB1_1100	PF03401	tricarboxylate		
D0IUT5	CtCNB1_4346	PF03401	tricarboxylate		
D0IX87	CtCNB1_4653	PF03401	tricarboxylate		
D0J4T5	CtCNB1_1597	PF03401	tricarboxylate		
D0J0G2	CtCNB1_2887	PF03401	tricarboxylate		
D0J0Z6	CtCNB1_0930	PF03401	tricarboxylate		
D0J735	CtCNB1_1996	PF00497	amino acid (glutamine/glutamate/aspartate?)		
A0A454XUP	CtCNB1_4579	PF09084	phosphonates		
D0IXR6	CtCNB1_0415	PF03401	tricarboxylate		
D0J445	CtCNB1_1492	PF03401	tricarboxylate		
D0J705	CtCNB1_1966	PF03401	tricarboxylate		
D0IZS4	CtCNB1_2781	PF03401	tricarboxylate		
D0J454	CtCNB1_3522	PF03480	C4-dicarboxylate		
D0J063	CtCNB1_0784	PF03401	tricarboxylate		
D0IWH1	CtCNB1_0243	PF03401	tricarboxylate		
A0A454XI80	CtCNB1_4364	PF03401	tricarboxylate		
D0J7F0	CtCNB1_4136	PF03401	tricarboxylate		
D0J3M0	CtCNB1_3468	PF03401	tricarboxylate		
A0A454XQX	CtCNB1_4766	PF03401	tricarboxylate		
D0J889	CtCNB1_4247	PF03401	tricarboxylate		
D0J310	CtCNB1_1244	PF03401	tricarboxylate		
D0IYT8	CtCNB1_2581	PF03401	tricarboxylate		
A0A454XNV	CtCNB1_4594	PF03480	C4-dicarboxylate		
D0J4R2	CtCNB1_1574	PF01497	iron-hydroxamate		
D0IVF9	CtCNB1_0149	PF09084	phosphonates		
D0J4Z3	CtCNB1_3680	PF02608	sugar		
D0J2H4	CtCNB1_1194	PF03401	tricarboxylate		
D0J2J9	CtCNB1_1219	PF13458	leucine/valine		

D0IYE0	CtCNB1_4791	PF13458	urea		
D0J3J7	CtCNB1_3445	PF13458	urea		
D0IZH8	CtCNB1_0682	PF03401	tricarboxylate		
D0J1D2	CtCNB1_3075	PF09084	glycine betaine		
D0J6A0	CtCNB1_1845	PF01497	iron-hydroxamate		
D0J0Z9	CtCNB1_0933	PF13416	sugar		
D0IUU1	CtCNB1_4352	PF13458	leucine/valine		
A0A454XZH!	CtCNB1_3679	PF02608	sugar		
D0IUS8	CtCNB1_4339	PF09084	nitrate/sulfonate/taurine		
D0J0J2	CtCNB1_2917	PF00497	amino acid (glutamine/glutamate/aspartate?)		
D0IV66	CtCNB1_0056	PF02608	sugar		
D0J1F4	CtCNB1_3097	PF03480	C4-dicarboxylate		
D0IUR7	CtCNB1_4328	PF13458	leucine/valine		
D0J3U5	CtCNB1_1392	PF04069	glycine betaine		
D0J4P3	CtCNB1_1555	PF03180	methionine		
D0J7T3	CtCNB1_2067	PF09084	phosphonates		
D0IYQ7	CtCNB1_0547	PF09084	nitrate/sulfonate/taurine		
D0IZ93	CtCNB1_0597	PF01497	iron-hydroxamate		
D0J819	CtCNB1_2153	PF03401	tricarboxylate		
D0J658	CtCNB1_1803	PF03401	tricarboxylate		
A0A454Y413	CtCNB1_1952	PF03180	methionine		
D0IW19	CtCNB1_4509	PF03401	tricarboxylate		
A0A454Y7F(	CtCNB1_0215	PF13458	urea		
D0J0N6	CtCNB1_2961	PF13458	urea		
D0IYU1	CtCNB1_2584	PF00497	Unclassified		
D0IUZ5	CtCNB1_4406	PF13458	urea		
D0J641	CtCNB1_1786	PF00496	oligopeptide		
D0J3I8	CtCNB1_3436	PF13458	urea		
D0J778	CtCNB1_4064	PF03401	tricarboxylate		
A0A454XWN	CtCNB1_2054	PF03180	methionine		
D0IVA6	CtCNB1_0096	PF13458	urea		
D0J1U4	CtCNB1_1094	PF03401	tricarboxylate		

D0J827	CtCNB1_2161	PF03401	tricarboxylate		
D0J3H0	CtCNB1_3418	PF03401	tricarboxylate		
D0J661	CtCNB1_1806	PF03401	tricarboxylate		
D0J6N8	CtCNB1_4009	PF03401	tricarboxylate		
D0J3U4	CtCNB1_1391	PF04069	glycine betaine		
D0IYD4	CtCNB1_4785	PF03401	tricarboxylate		
A0A454XQR	CtCNB1_4182	PF00497	amino acid (glutamine/glutamate/aspartate?)		
D0J569	CtCNB1_3756	PF03401	tricarboxylate		
A0A454XRT4	CtCNB1_3800	PF09084	glycine betaine		
D0J770	CtCNB1_2031	PF03401	tricarboxylate		
D0J565	CtCNB1_3752	PF03401	tricarboxylate		
D0IX48	CtCNB1_4614	PF03401	tricarboxylate		
D0IX40	CtCNB1_4606	PF03401	tricarboxylate		
A0A454XV58	CtCNB1_1485	PF09084	amino acid (glutamine/glutamate/aspartate?)		
D0IWF3	CtCNB1_0225	PF03401	tricarboxylate		
D0IWG6	CtCNB1_0238	PF03401	tricarboxylate		
D0J085	CtCNB1_0806	PF00496	dipeptide/oligopeptide		
D0J6T7	CtCNB1_1898	PF03401	tricarboxylate		
D0J1E1	CtCNB1_3084	PF03401	tricarboxylate		
D0J2Y5	CtCNB1_3362	PF03401	tricarboxylate		
D0J5L2	CtCNB1_1739	PF13458	leucine/valine		
D0J2Y4	CtCNB1_3361	PF03401	tricarboxylate		
D0J2C7	CtCNB1_1147	PF13458	urea		
D0J1T3	CtCNB1_1083	PF13458	urea		
D0J6E1	CtCNB1_1886	PF01497	cobalamin		
D0J3N1	CtCNB1_3479	PF13458	urea		
A0A454XQT	CtCNB1_2167	PF13458	leucine/valine		
D0J301	CtCNB1_3378	PF03401	tricarboxylate		
D0IZU0	CtCNB1_2797	PF03401	tricarboxylate		
D0J327	CtCNB1_1261	PF03401	tricarboxylate		
D0IW15	CtCNB1_4505	PF03401	tricarboxylate		

D0IXC9	CtCNB1_4695	PF03401	tricarboxylate		
D0IYS2	CtCNB1_0562	PF03480	C4-dicarboxylate		
D0J3L3	CtCNB1_3461	PF02608	sugar		
D0J306	CtCNB1_3383	PF03401	tricarboxylate		
D0J1Y8	CtCNB1_3145	PF03401	tricarboxylate		
D0J2J3	CtCNB1_1213	PF03401	tricarboxylate		
D0J8D9	CtCNB1_4297	PF03401	tricarboxylate		
D0J570	CtCNB1_3757	PF03401	tricarboxylate		
D0IWE7	CtCNB1_0219	PF03401	tricarboxylate		
D0J7L1	CtCNB1_0005	PF03401	tricarboxylate		
D0IYN5	CtCNB1_0525	PF03480	C4-dicarboxylate		
A0A454Y4K3	CtCNB1_3520	PF03480	C4-dicarboxylate		
D0J8B1	CtCNB1_4269	PF03401	tricarboxylate		
D0J1D1	CtCNB1_3074	PF09084	nitrate/sulfonate/taurine		
D0J4F2	CtCNB1_3620	PF13458	leucine/valine		
D0J7L6	CtCNB1_0010	PF03401	tricarboxylate		
D0J4F8	CtCNB1_3626	PF03401	tricarboxylate		
D0IWZ9	CtCNB1_2412	PF03401	tricarboxylate		
D0J328	CtCNB1_1262	PF03401	tricarboxylate		
D0J294	CtCNB1_1114	PF03401	tricarboxylate		
D0IVM6	CtCNB1_2208	PF03401	tricarboxylate		
D0IW09	CtCNB1_4499	PF03401	tricarboxylate		
D0J7Q3	CtCNB1_2037	PF03401	tricarboxylate		
D0J6C0	CtCNB1_1865	PF00497	amino acid (glutamine/glutamate/aspartate?)		
D0J246	CtCNB1_3203	PF12849	phosphate		
D0IVH0	CtCNB1_0160	PF09084	nitrate/sulfonate/taurine		
D0J2L6	CtCNB1_1236	PF09084	nitrate/sulfonate/taurine		
A0A454XNR[illegible]	CtCNB1_3427	PF03480	C4-dicarboxylate		
D0IWX1	CtCNB1_2384	PF04392	Unclassified		
D0J068	CtCNB1_0789	PF13416	sugar		
D0J2Y2	CtCNB1_3359	PF03401	tricarboxylate		
D0J0I2	CtCNB1_2907	PF03180	methionine		

D0J6K3	CtCNB1_3974	PF03401	tricarboxylate		
D0J0C1	CtCNB1_0842	PF03401	tricarboxylate		
D0IUW5	CtCNB1_4376	PF13458	leucine/valine		
D0IZJ4	CtCNB1_0698	PF00497	amino acid (glutamine/glutamate/aspartate?)		
D0IXA0	CtCNB1_4666	PF00497	amino acid (glutamine/glutamate/aspartate?)		
D0IVL5	CtCNB1_2197	PF03401	tricarboxylate		
D0J2G1	CtCNB1_1181	PF01497	iron-hydroxamate		
D0J2N6	CtCNB1_3263	PF03401	tricarboxylate		
D0IWG1	CtCNB1_0233	PF13458	urea		
D0J6B9	CtCNB1_1864	PF03401	tricarboxylate		
D0IV07	CtCNB1_4418	PF00496	dipeptide/oligopeptide		
D0J3K4	CtCNB1_3452	PF03401	tricarboxylate		
A0A454XRN	CtCNB1_2959	PF13458	urea		
D0J504	CtCNB1_3691	PF01497	iron-hydroxamate		
D0J2A0	CtCNB1_1120	PF03401	tricarboxylate		
D0IWD4	CtCNB1_0206	PF03401	tricarboxylate		
D0IUX9	CtCNB1_4390	PF01497	iron-hydroxamate		
D0J7H9	CtCNB1_4165	PF03480	C4-dicarboxylate		
A0A454XNT	CtCNB1_0055	PF03401	tricarboxylate		
D0IWD5	CtCNB1_0207	PF03401	tricarboxylate		
D0IV71	CtCNB1_0061	PF00497	amino acid (glutamine/glutamate/aspartate?)		
***Azotobacter vinelandii* DJ**					
Number of ORFs in the proteome: 4990					
Number of SBPs: 70 (1.40% of total)					
Lifestyle Summary: SOIL					
**SeqID**	**Gene Locus**	**Pfam Code**	**Ligand prediction by TransportDB**	**Experimentally identified ligands**	**References/pdb accession codes**
C1DLI2	Avin_06790	PF00497	amino acid (glutamine/glutamate/aspartate?)		

C1DGS7	Avin_44550	PF09084	phosphonates		
C1DID8	Avin_03750	PF03401	tricarboxylate		
C1DFC2	Avin_21240	PF03401	tricarboxylate		
C1DRJ7	Avin_15200	PF00497	amino acid (glutamine/glutamate/aspartate?)		
C1DHN6	Avin_24440	PF00496	dipeptide/oligopeptide		
C1DLI4	Avin_06810	PF01497	iron-hydroxamate		
C1DJQ0	Avin_26430	PF01497	iron-hydroxamate		
C1DNA8	Avin_31110	PF09084	nitrate/sulfonate/taurine		
C1DM44	Avin_29650	PF00496	oligopeptide		
C1DNU9	Avin_31390	PF09084	nitrate/sulfonate/taurine		
C1DLD0	Avin_50340	PF01497	iron-hydroxamate		
C1DSE2	Avin_16838	PF00497	amino acid (glutamine/glutamate/aspartate?)		
C1DFD5	Avin_21410	PF00497	amino acid (glutamine/glutamate/aspartate?)		
C1DFT7	Avin_43620	PF13407	ribose		
C1DGJ8	Avin_43730	PF13458	leucine/valine		
C1DNX8	Avin_31680	PF09084	nitrate/sulfonate/taurine		
C1DLB1	Avin_50140	PF13407	xylose		
C1DM10	Avin_29280	PF01547	sugar		
C1DNV0	Avin_31400	PF09084	phosphonates		
C1DFF5	Avin_21640	PF09084	nitrate/sulfonate/taurine		
C1DGC2	Avin_22420	PF09084	phosphonates		
C1DS61	Avin_37920	PF13407	rhamnose		
C1DDV8	Avin_18670	PF00496	dipeptide/oligopeptide		
C1DMM8	Avin_08130	PF09084	nitrate/sulfonate/taurine		
C1DN49	Avin_30510	PF09084	nitrate/sulfonate/taurine		
C1DKR2	Avin_27400	PF01547	sugar		
C1DM19	Avin_29390	PF09084	glycine betaine		
C1DE19	Avin_39880	PF01497	iron-hydroxamate		
C1DLC8	Avin_50320	PF13407	rhamnose		
C1DGK0	Avin_43750	PF13458	leucine/valine		

C1DGS4	Avin_44520	PF00497	amino acid (glutamine/glutamate/aspartate?)		
C1DNU8	Avin_31380	PF09084	nitrate/sulfonate/taurine		
C1DPE4	Avin_11470	PF16868	C4-dicarboxylate		
C1DG23	Avin_00830	PF01297	zinc ion		
C1DFT8	Avin_43630	PF13407	ribose		
C1DL51	Avin_49490	PF03401	tricarboxylate		
C1DL48	Avin_49460	PF03401	tricarboxylate		
C1DMJ2	Avin_51940	PF00496	dipeptide/oligopeptide		
C1DFY5	Avin_00450	PF01497	iron-hydroxamate		
C1DFS6	Avin_43510	PF13458	branched-chain amino acid		
C1DID9	Avin_03760	PF09084	nitrate/sulfonate/taurine		
C1DGJ7	Avin_43720	PF13458	leucine/valine		
C1DK05	Avin_48060	PF00496	dipeptide/oligopeptide		
C1DLN0	Avin_07320	PF00497	amino acid (glutamine/glutamate/aspartate?)		
C1DNU6	Avin_31360	PF09084	nitrate/sulfonate/taurine		
C1DMN2	Avin_08170	PF09084	nitrate/sulfonate/taurine		
C1DNW1	Avin_31510	PF03180	methionine		
C1DKB5	Avin_49230	PF13407	rhamnose		
C1DEI9	Avin_19650	PF13458	leucine/valine		
C1DG88	Avin_22080	PF13407	xylose		
C1DG77	Avin_21970	PF09084	nitrate/sulfonate/taurine		
C1DIC8	Avin_03650	PF03480	C4-dicarboxylate		
C1DJ00	Avin_47140	PF01497	iron-hydroxamate		
C1DJM9	Avin_26220	PF13407	xylose		
C1DGD0	Avin_22500	PF09084	phosphonates		
C1DQ88	Avin_13140	PF04348	Not annotated in TransportDB		
C1DG71	Avin_21910	PF09084	nitrate/sulfonate/taurine		
C1DFG3	Avin_21720	PF09084	nitrate/sulfonate/taurine		
C1DK56	Avin_48600	PF12849	phosphate		
C1DE36	Avin_40070	PF09084	nitrate/sulfonate/taurine		
C1DK71	Avin_48760	PF13416	spermidine/putrescine		

C1DN84	Avin_30870	PF01497	iron-hydroxamate		
C1DJM1	Avin_26140	PF13458	leucine/valine		
C1DLB0	Avin_50130	PF13407	xylose		
C1DM43	Avin_29640	PF00496	oligopeptide		
C1DJ76	Avin_04030	PF13407	rhamnose		
C1DQ27	Avin_12520	PF00496	dipeptide/oligopeptide		
C1DKH5	Avin_05850	PF03401	tricarboxylate		
C1DMG3	Avin_51630	PF13407	rhamnose		
***Pseudomonas aeruginosa* PAO1**					
Number of ORFs in the proteome: 5564					
Number of SBPs: 93 (1.67% of total)					
Lifestyle Summary: HUMAN/ANIMAL PATHOGEN					
**SeqID**	**Gene Locus**	**Pfam Code**	**Ligand prediction by TransportDB**	**Experimentally identified ligands**	**References/pdb accession codes**
Q9I6J0	PA0301	PF13416	spermidine/putrescine	Spermidine	doi: 10.1016/j.jmb.2012.01.010
Q9I6J1	PA0300	PF13416	spermidine/putrescine	Putrescine and spermidine	doi: 10.1016/j.jmb.2012.01.010
Q51371	PA3545	PF05048	Not annotated in TransportDB		
Q01269	PA3475	PF00497	amino acid (glutamine/glutamate/aspartate?)		
G3XDA8	PA5369	PF12849	phosphate	Phosphate	doi: 10.1111/j.1432-1033.1984.tb08508.x; doi: 10.1111/j.1432-1033.1984.tb08508.x; doi: 10.1107/S2053230X14010279
P35482	PA0688	PF12849	phosphate		
Q9HU18	PA5167	PF03480	C4-dicarboxylate	Succinate, fumarate and malate	doi: 10.1128/JB.05074-11
P21175	PA1074	PF13458	leucine/valine	Leucine, isoleucine. Valine, threonine, L-homoserine	doi: 10.1128/jb.171.11.6300-6306.1989; doi: 10.3390/ijms20205156
Q9HYL1	PA3393	PF05048	Not annotated in TransportDB		

Q9I3I2	PA1531	PF00497	amino acid (glutamine/glutamate/aspartate?)		
Q9I0P1	PA2596	PF09084	nitrate/sulfonate/taurine		
Q9I0P2	PA2595	PF09084	nitrate/sulfonate/taurine		
Q9I484	PA1260	PF00497	amino acid (glutamine/glutamate/aspartate?)		
Q9I2T3	PA1811	PF00496	oligopeptide		
Q9HY96	PA3513	PF09084	nitrate/sulfonate/taurine		
Q9HX85	PA3931	PF03180	methionine		
Q9HVS4	PA4497	PF00496	dipeptide/oligopeptide	Many different tripeptides	doi: 10.3390/ijms20205156
Q9HVR9	PA4502	PF00496	dipeptide/oligopeptide		
G3XD96	PA0186	PF09084	nitrate/sulfonate/taurine		
Q9HTT0	PA5270	PF00497	amino acid (glutamine/glutamate/aspartate?)		
Q9HX00	PA4027	PF00497	amino acid (glutamine/glutamate/aspartate?)		
Q9I0D1	PA2711	PF13416	spermidine/putrescine		
Q9HUA7	PA5076	PF00497	amino acid (glutamine/glutamate/aspartate?)		
Q9I402	PA1342	PF00497	amino acid (glutamine/glutamate/aspartate?)	L-Asp, L-Glu	doi: 10.3390/ijms20205156
Q9HUI0	PA4985	PF13416	spermidine/putrescine		
Q9I6H7	PA0314	PF00497	amino acid (glutamine/glutamate/aspartate?)		
Q9HXC4	PA3889	PF04069	glycine betaine	Glycine betaine	doi: 10.3390/ijms20205156
Q9HZ48	PA3190	PF01547	sugar		
Q9HWY3	PA4045	PF01497	iron-hydroxamate		
Q9I0P5	PA2592	PF13416	spermidine/putrescine	Putrescine, cadaverine	doi: 10.3390/ijms20205156
Q9HTI6	PA5378	PF04069	choline		
Q9I6R6	PA0222	PF13416	spermidine/putrescine	GABA, 4-aminovalerate	doi: 10.3390/ijms20205156
Q9HU45	PA5139	PF00497	amino acid (glutamine/glutamate/aspartate?)		

Q9I6G8	PA0323	PF13416	spermidine/putrescine		
Q9I3T4	PA1410	PF13416	spermidine/putrescine		
Q9I5T4	PA0604	PF13416	spermidine/putrescine		
Q9HU31	PA5153	PF00497	amino acid (glutamine/glutamate/aspartate?)		
Q9HV90	PA4708	PF01497	iron-hydroxamate		
Q9I6Y6	PA0146	PF02608	sugar		
Q9I1E0	PA2338	PF01547	sugar	Mannitol	doi: 10.3390/ijms20205156
Q9I2T4	PA1810	PF00496	oligopeptide		
Q9I1C9	PA2349	PF03180	methionine		
Q9I5H5	PA0754	PF03401	tricarboxylate		
Q9HT75	PA5498	PF01297	zinc ion		
Q9I6T5	PA0203	PF13416	spermidine/putrescine		
Q9I7A0	PA0030	PF04069	choline		
Q9HX78	PA3938	PF09084	glycine betaine		
Q9HYG1	PA3445	PF09084	glycine betaine		
Q9HXG8	PA3836	PF04392	Unclassified		
Q9HT29	PA5545	PF16868	phosphonates		
Q9HYF7	PA3449	PF09084	phosphonates		
Q9HZT4	PA2913	PF01497	cobalamin		
Q9HXE1	PA3865	PF00497	amino acid (glutamine/glutamate/aspartate?)		
Q9HZU5	PA2902	PF00497	amino acid (glutamine/glutamate/aspartate?)		
Q9HVZ2	PA4423	PF04348	Not annotated in TransportDB		
Q9I0P3	PA2594	PF09084	nitrate/sulfonate/taurine		
Q9I2F8	PA1946	PF13407	rhamnose	D-ribose; D-allose; D-arabinose	doi: 10.3390/ijms20205156
Q9HXF8	PA3848	PF16868	C4-dicarboxylate		
Q9HTN7	PA5317	PF00496	dipeptide/oligopeptide		
Q9HT68	PA5505	PF03180	methionine		
Q9HU46	PA5138	PF00497	amino acid (glutamine/glutamate/aspartate?)		

Q9I6J6	PA0295	PF13416	spermidine/putrescine		
Q9HU47	PA5137	PF00497	amino acid (glutamine/glutamate/aspartate?)		
Q9HU80	PA5103	PF04069	glycine betaine		
Q9HY16	PA3610	PF13416	spermidine/putrescine		
Q9HVA8	PA4687	PF13416	iron(III)	Iron, gallium	doi: 10.1021/acsinfecdis.9b00271
Q9HZ04	PA3236	PF04069	glycine betaine		
Q9HUQ0	PA4913	PF13458	leucine/valine	Branched chain amino acids, Gly, Ala, Pro	doi: 10.3390/ijms20205156
Q9I5T6	PA0602	PF13416	spermidine/putrescine		
Q9I257	PA2058	PF00496	oligopeptide		
Q9I174	PA2407	PF01297	manganese/zinc ion	Iron, magnesium, zinc, calcium, manganesium, nickel, zinc, cobaltum, copper, cadmium	doi: 10.1111/febs.15004
Q9HZS4	PA2923	PF00497	amino acid (glutamine/glutamate/aspartate?)		
Q9HVS1	PA4500	PF00496	dipeptide/oligopeptide	Many different dipeptides	doi: 10.3390/ijms20205156
Q9I0N8	PA2599	PF09084	glycine betaine		
Q9HU87	PA5096	PF04069	glycine betaine		
Q9HTH6	PA5388	PF04069	choline		
Q9HXE8	PA3858	PF00497	amino acid (glutamine/glutamate/aspartate?)		
Q9I561	PA0884	PF03480	C4-dicarboxylate		
Q9HWM2	PA4159	PF01497	iron-hydroxamate		
G3XD47	PA0888	PF00497	amino acid (glutamine/glutamate/aspartate?)	Arginine, Phospho-L-arginine	doi: 10.3390/ijms20205156
Q9HWI6	PA4195	PF00497	amino acid (glutamine/glutamate/aspartate?)		
Q9HVS5	PA4496	PF00496	dipeptide/oligopeptide		
Q9HVH5	PA4616	PF03480	C4-dicarboxylate	L-malate	doi: 10.1021/bi501388y.

Q9I1Q8	PA2209	PF03401	tricarboxylate		
Q9I171	PA2410	PF01297	manganese/zinc ion		
Q9HYX9	PA3261	PF00497	amino acid (glutamine/glutamate/aspartate?)		
Q9HXL6	PA3779	PF03480	C4-dicarboxylate		
Q9HUA1	PA5082	PF00497	amino acid (glutamine/glutamate/aspartate?)		
Q9I3B7	PA1604	PF00497	amino acid (glutamine/glutamate/aspartate?)		
Q9I5N7	PA0689	PF12849	phosphate		
Q9HX57	PA3959	PF00497	Unclassified		
Q9I1R3	PA2204	PF00497	amino acid (glutamine/glutamate/aspartate?)		
Q9HWX9	PA4049	PF00497	amino acid (glutamine/glutamate/aspartate?)		
***Photorhabdus luminescens* subsp. *laumondii* TTO1**					
Number of ORFs in the proteome: 4556					
Number of SBPs: 33 (0.72% of total)					
Lifestyle Summary: HUMAN/ANIMAL PATHOGEN					
**SeqID**	**Gene Locus**	**Pfam Code**	**Ligand prediction by TransportDB**	**Experimentally identified ligands**	**References/pdb accession codes**
Q7MB18	plu3146	PF13407	rhamnose		
Q7N842	plu0905	PF01497	iron-hydroxamate		
Q7N985	plu0458	PF13416	sugar (maltose?)		
Q7N4I7	plu2350	PF00496	oligopeptide		
Q7N006	plu4098	PF13458	leucine/valine		
Q7N460	plu2494	PF00496	oligopeptide		
Q7N8U8	plu0619	PF01547	thiamin		
Q7MZ24	plu4484	PF00497	amino acid (glutamine/glutamate/aspartate?)		
Q7N3X5	plu2587	PF00496	oligopeptide		
Q7N777	plu1281	PF04069	glycine betaine		

Q7MZ21	plu4487	PF00497	amino acid (glutamine/glutamate/aspartate?)		
Q7N9X9	plu0172	PF03480	C4-dicarboxylate		
Q7N461	plu2493	PF00496	oligopeptide		
Q7N9U7	plu0214	PF12849	phosphate		
Q7N9M2	plu0300	PF00496	dipeptide/oligopeptide		
Q7MZL6	plu4267	PF13458	leucine/valine		
Q7N3S9	plu2634	PF01497	iron-hydroxamate		
Q7N9U2	plu0219	PF00497	amino acid (glutamine/glutamate/aspartate?)		
Q7N751	plu1307	PF00497	amino acid (glutamine/glutamate/aspartate?)		
Q7N091	plu4002	PF04348	leucine/valine		
Q7NA77	plu0058	PF13407	ribose		
Q7N8M4	plu0695	PF03180	methionine		
Q7N3P3	plu2672	PF01297	manganese/zinc ion		
Q7N0Z1	plu3738	PF01497	iron-hydroxamate		
Q7N6G3	plu1587	PF00497	amino acid (glutamine/glutamate/aspartate?)		
Q7MZ54	plu4447	PF01497	iron-hydroxamate		
Q7N2A7	plu3181	PF03401	tricarboxylate		
Q7N3Y4	plu2578	PF00496	oligopeptide		
Q7MYQ2	plu4623	PF01497	iron-hydroxamate		
Q7N7H5	plu1174	PF01497	iron-hydroxamate		
Q7N544	plu2115	PF01297	zinc ion		
Q7N368	plu2853	PF01497	iron-hydroxamate		
Q7N4V3	plu2212	PF00496	oligopeptide		
***Pseudomonas syringae* pv. tomato DC3000**					
Number of ORFs in the proteome: 5431					
Number of SBPs: 95 (1.75% of total)					
Lifestyle Summary: PLANT PATHOGEN					

SeqID	Gene Locus	Pfam Code	Ligand prediction by TransportDB	Experimentally identified ligands	References/pdb accession codes
Q885K6	PSPTO_1826	PF00497	amino acid (glutamine/glutamate/aspartate?)		
Q88B53	PSPTO_0166	PF04069	choline		
Q881A0	PSPTO_2996	PF13407	ribose		
Q883E1	PSPTO_2423	PF09084	nitrate/sulfonate/taurine		
Q87WL2	PSPTO_4534	PF00496	dipeptide/oligopeptide		
Q880R4	PSPTO_3088	PF00496	dipeptide/oligopeptide		
Q882J2	PSPTO_2635	PF01297	manganese/zinc ion		
Q87XP0	PSPTO_4136	PF00497	amino acid (glutamine/glutamate/aspartate?)		
Q881U7	PSPTO_2785	PF13416	spermidine/putrescine		
Q888X5	PSPTO_0889	PF01547	sugar		
Q87WH3	PSPTO_4577	PF04069	glycine betaine		
Q88AM5	PSPTO_0364	PF01547	sugar		
Q880R9	PSPTO_3082	PF00497	amino acid (glutamine/glutamate/aspartate?)		
Q889I6	PSPTO_0763	PF01497	iron-hydroxamate		
Q886J4	PSPTO_1585	PF00497	amino acid (glutamine/glutamate/aspartate?)		
Q887K0	PSPTO_1292	PF13416	sugar		
Q889J0	PSPTO_0759	PF02608	sugar		
Q87UN6	PSPTO_5260	PF03180	methionine		
Q88BD0	PSPTO_0087	PF00496	dipeptide/oligopeptide		
Q87XE0	PSPTO_4238	PF09084	nitrate/sulfonate/taurine		
Q883J0	PSPTO_2367	PF13407	rhamnose		
Q87UM8	PSPTO_5268	PF01297	zinc ion		
Q87YS6	PSPTO_3716	PF00496	oligopeptide		
Q881R9	PSPTO_2814	PF00496	dipeptide/oligopeptide		
Q87WI7	PSPTO_4559	PF00496	dipeptide/oligopeptide		
Q881Z8	PSPTO_2731	PF13407	ribose		
Q880Z3	PSPTO_3003	PF13407	xylose		

Q886F7	PSPTO_1622	PF04069	glycine betaine		
Q87WI8	PSPTO_4558	PF00496	dipeptide/oligopeptide		
Q887X1	PSPTO_1159	PF02608	sugar		
Q87UE1	PSPTO_5358	PF00497	amino acid (glutamine/glutamate/aspartate?)		
Q880V2	PSPTO_3049	PF13407	xylose		
Q880U3	PSPTO_3058	PF04069	glycine betaine		
Q88AC8	PSPTO_0464	PF04069	choline		
Q87UW2	PSPTO_5180	PF00497	amino acid (glutamine/glutamate/aspartate?)		
Q87UG5	PSPTO_5333	PF00497	amino acid (glutamine/glutamate/aspartate?)		
Q87WI9	PSPTO_4557	PF00496	dipeptide/oligopeptide		
Q882K0	PSPTO_2627	PF00497	amino acid (glutamine/glutamate/aspartate?)		
Q881A9	PSPTO_2987	PF13458	branched-chain amino acid		
Q885P3	PSPTO_1788	PF09084	nitrate/sulfonate/taurine		
Q87XI1	PSPTO_4197	PF13416	spermidine/putrescine		
Q88A34	PSPTO_0563	PF13416	spermidine/putrescine		
Q87UT0	PSPTO_5216	PF03180	methionine		
Q887N6	PSPTO_1255	PF00497	amino acid (glutamine/glutamate/aspartate?)		
Q87UJ0	PSPTO_5307	PF13416	spermidine/putrescine		
Q880X1	PSPTO_3029	PF00496	oligopeptide		
Q882V4	PSPTO_2518	PF13416	2-aminoethylphosphonate		
Q881V7	PSPTO_2775	PF00497	amino acid (glutamine/glutamate/aspartate?)		
Q87UJ1	PSPTO_5306	PF13416	spermidine/putrescine		
Q87VC9	PSPTO_5010	PF00497	amino acid (glutamine/glutamate/aspartate?)		
Q884A9	PSPTO_2187	PF00497	amino acid (glutamine/glutamate/aspartate?)		
Q87ZD9	PSPTO_3490	PF13407	xylose		

Q880A3	PSPTO_3269	PF12849	phosphate		
Q887Z6	PSPTO_1134	PF00497	amino acid (glutamine/glutamate/aspartate?)		
Q886H9	PSPTO_1600	PF04069	glycine betaine		
Q882P9	PSPTO_2576	PF00496	dipeptide/oligopeptide		
Q87XE6	PSPTO_4232	PF03401	tricarboxylate		
Q87YQ9	PSPTO_3735	PF13416	sugar		
Q87WX4	PSPTO_4419	PF04348	leucine/valine		
Q882Z9	PSPTO_2473	PF13407	xylose		
Q87VL7	PSPTO_4919	PF13458	leucine/valine		
Q87XK7	PSPTO_4171	PF00497	amino acid (glutamine/glutamate/aspartate?)		
Q87UV5	PSPTO_5187	PF03180	methionine		
Q882I9	PSPTO_2638	PF00532	ribose		
Q882G1	PSPTO_2667	PF13416	spermidine/putrescine		
Q87UI1	PSPTO_5316	PF09084	glycine betaine		
Q883G4	PSPTO_2399	PF13407	xylose		
Q87WI5	PSPTO_4561	PF00496	dipeptide/oligopeptide		
Q881I1	PSPTO_2912	PF00497	amino acid (glutamine/glutamate/aspartate?)		
Q880B6	PSPTO_3256	PF01497	cobalamin		
Q885P0	PSPTO_1791	PF09084	phosphonates		
Q882C2	PSPTO_2707	PF01547	sugar		
Q887D6	PSPTO_1360	PF02608	sugar		
Q884F2	PSPTO_2141	PF01297	manganese/zinc ion		
Q887G1	PSPTO_1335	PF00497	amino acid (glutamine/glutamate/aspartate?)		
Q87U28	PSPTO_5487	PF12849	phosphate		
Q889T3	PSPTO_0664	PF13416	sugar		
Q87UM5	PSPTO_5271	PF04069	glycine betaine		
Q88AS2	PSPTO_0314	PF13416	2-aminoethylphosphonate		
Q880C3	PSPTO_3248	PF00496	dipeptide/oligopeptide		

Q880Y8	PSPTO_3008	PF00497	amino acid (glutamine/glutamate/aspartate?)		
Q881U4	PSPTO_2788	PF09084	nitrate/sulfonate/taurine		
Q87YS5	PSPTO_3717	PF00496	oligopeptide		
Q884F5	PSPTO_2138	PF01297	manganese/zinc ion		
Q87YZ2	PSPTO_3647	PF00497	amino acid (glutamine/glutamate/aspartate?)		
Q886U0	PSPTO_1486	PF00497	Not annotated in TransportDB		
Q87XR8	PSPTO_4108	PF13458	leucine/valine		
Q87UQ1	PSPTO_5245	PF00497	amino acid (glutamine/glutamate/aspartate?)		
Q88AY4	PSPTO_0249	PF00496	dipeptide/oligopeptide		
Q88AW6	PSPTO_0267	PF00496	oligopeptide		
Q87UH8	PSPTO_5319	PF09084	glycine betaine		
Q87TV8	PSPTO_5562	PF01497	cobalamin		
Q884R4	PSPTO_2024	PF00497	amino acid (glutamine/glutamate/aspartate?)		
Q881D2	PSPTO_2962	PF13458	leucine/valine		
Q888H3	PSPTO_1051	PF03480	C4-dicarboxylate		
***Bordetella pertussis* Tohama I**					
Number of ORFs in the proteome: 3258					
Number of SBPs: 167 (5.13% of total)					
Lifestyle Summary: HUMAN/ANIMAL PATHOGEN					
**SeqID**	**Gene Locus**	**Pfam Code**	**Ligand prediction by TransportDB**	**Experimentally identified ligands**	**References/pdb accession codes**
O30446	BP0148	PF03401	tricarboxylate		
Q45389	BP3782	PF03401	tricarboxylate		
Q7VY55	BP1507	PF00497	amino acid (glutamine/glutamate/aspartate?)		
Q7VYN8	BP1277	PF13458	leucine/valine		
Q7VU18	BP3322	PF13458	leucine/valine		
Q7VSG4	BP0461	PF09084	nitrate/sulfonate/taurine		

Q7VS23	BP0633	PF03401	tricarboxylate		
Q7VXB9	BP1864	PF03480	C4-dicarboxylate		
Q7VTS6	BP3434	PF03180	methionine		
Q7VXF2	BP1823	PF00496	dipeptide/oligopeptide		
Q7VVQ0	BP2594	PF00496	dipeptide/oligopeptide		
Q7VZ63	BP1071	PF12849	phosphate		
Q7VYI3	BP1354	PF03401	tricarboxylate		
Q7VXF5	BP1819	PF13416	sugar		
Q7VY56	BP1506	PF13458	leucine/valine		
Q7VY76	BP1480	PF12849	tungsten		
Q7W061	BP0301	PF13458	urea		
Q7VSK8	BP0403	PF16868	phosphonates		
Q7VVC9	BP2747	PF13458	leucine/valine		
Q7VS60	BP0589	PF03480	C4-dicarboxylate		
Q7VT77	BP3674	PF09084	glycine betaine		
Q7VWG3	BP2293	PF00496	dipeptide/oligopeptide		
Q7VYN5	BP1281	PF13416	sugar		
Q7VYH5	BP1364	PF00497	amino acid (glutamine/glutamate/aspartate?)		
Q7VSR6	BP3862	PF00496	dipeptide/oligopeptide		
Q7VUD9	BP3159	PF00496	dipeptide/oligopeptide		
Q7W0P0	BP0057	PF00497	amino acid (glutamine/glutamate/aspartate?)		
Q7VZJ2	BP0913	PF09084	nitrate/sulfonate/taurine		
Q7VS56	BP0593	PF09084	nitrate/sulfonate/taurine		
Q7VWX0	BP2066	PF03401	tricarboxylate		
Q7VVL6	BP2638	PF13416	sugar		
Q7W018	BP0683	PF09084	Not annotated in TransportDB		
Q7VW51	BP2418	PF13458	urea		
Q7VRZ6	BP0663	PF09084	nitrate/sulfonate/taurine		
Q7VYU9	BP1208	PF03401	tricarboxylate		
Q7VX60	BP1948	PF13458	urea		
Q7VV16	BP2895	PF03401	tricarboxylate		

Q7VTG8	BP3572	PF04069	glycine betaine		
Q7VXR3	BP1675	PF03401	tricarboxylate		
Q7U360	BP2835	PF13416	sugar		
Q7VU78	BP3245	PF03401	tricarboxylate		
Q7W032	BP0334	PF03401	tricarboxylate		
Q7VVN1	BP2616	PF09084	nitrate/sulfonate/taurine		
Q7VS15	BP0641	PF03401	tricarboxylate		
Q7VZH6	BP0931	PF03401	tricarboxylate		
Q7VSH2	BP0452	PF03401	tricarboxylate		
Q7VS81	BP0561	PF03401	tricarboxylate		
Q7VW44	BP2425	PF03401	tricarboxylate		
Q7W0N0	BP0069	PF03480	C4-dicarboxylate		
Q7VYX9	BP1170	PF03401	tricarboxylate		
Q7VT58	BP3696	PF03401	tricarboxylate		
Q7VX52	BP1965	PF03401	tricarboxylate		
Q79GS0	BP2231	PF04392	Unclassified		
Q7VZW5	BP0765	PF00497	amino acid (glutamine/glutamate/aspartate?)		
Q7VUA0	BP3219	PF03401	tricarboxylate		
Q7VZS1	BP0819	PF03401	tricarboxylate		
Q7W096	BP0254	PF03401	tricarboxylate		
Q7W019	BP0682	PF03401	tricarboxylate	Nicotinate, nicotinamide, citrate, benzoate, quinaldic acid	doi: 10.1016/j.jmb.2007.08.006
Q7VSP0	BP0363	PF00497	amino acid (glutamine/glutamate/aspartate?)		
Q7VUV7	BP2963	PF03480	C4-dicarboxylate		
Q7VTR0	BP3454	PF03401	tricarboxylate		
Q7VVJ6	BP2661	PF03480	C4-dicarboxylate		
Q7VZW0	BP0771	PF03401	tricarboxylate		
Q7VZI0	BP0927	PF03401	tricarboxylate		
Q7VTJ2	BP3544	PF03401	tricarboxylate		
Q7VVW2	BP2523	PF03401	tricarboxylate		

Q7W036	BP0330	PF03401	tricarboxylate		
Q7VVI8	BP2671	PF03480	C4-dicarboxylate		
Q7VV65	BP2826	PF03401	tricarboxylate		
Q7VWA8	BP2352	PF03480	C4-dicarboxylate		
Q7VY31	BP1532	PF00497	amino acid (glutamine/glutamate/aspartate?)		
Q7VZS8	BP0810	PF03401	tricarboxylate		
Q7W0B2	BP0237	PF03401	tricarboxylate		
Q7VTM2	BP3501	PF03401	tricarboxylate		
Q7VY71	BP1487	PF03480	C4-dicarboxylate		
Q7VST2	BP3843	PF03401	tricarboxylate		
Q7VRY8	BP0671	PF03401	tricarboxylate		
Q7VZS3	BP0817	PF03401	tricarboxylate		
Q7VYZ0	BP1160	PF03401	tricarboxylate		
Q7VUI5	BP3106	PF03401	tricarboxylate		
Q7VV33	BP2873	PF03401	tricarboxylate		
Q7VXB1	BP1891	PF03480	C4-dicarboxylate	Pyroglutamic acid	doi: 10.1016/j.jmb.2007.04.047
Q7VXN9	BP1708	PF09084	phosphonates		
Q7W0E0	BP0200	PF03401	tricarboxylate		
Q7VVK9	BP2647	PF03401	tricarboxylate		
Q7VSJ7	BP0418	PF03401	tricarboxylate		
Q7W0B4	BP0234	PF03480	C4-dicarboxylate		
Q7VT85	BP3666	PF03401	tricarboxylate		
Q7VXP2	BP1705	PF03480	C4-dicarboxylate		
Q7VSE5	BP0486	PF03401	tricarboxylate		
Q7W0J5	BP0121	PF13416	sugar		
Q7VSU5	BP3827	PF03401	tricarboxylate		
Q7VTS1	BP3440	PF03401	tricarboxylate	Aspartate	doi: 10.1016/j.jmb.2005.11.096
Q7VUC5	BP3178	PF03401	tricarboxylate		
Q7VYI0	BP1358	PF03401	tricarboxylate		
Q7VXT9	BP1644	PF03401	tricarboxylate		
Q7VT56	BP3700	PF03401	tricarboxylate		
Q7VV83	BP2802	PF03401	tricarboxylate		

Q7VT81	BP3670	PF03401	tricarboxylate		
Q7VWT9	BP2103	PF03401	tricarboxylate		
Q7VXQ9	BP1685	PF03401	tricarboxylate		
Q7VVK4	BP2653	PF03401	tricarboxylate		
Q7VT01	BP3756	PF03401	tricarboxylate		
Q7VRZ5	BP0664	PF03401	tricarboxylate		
Q7VVX2	BP2513	PF03401	tricarboxylate		
Q7VZH3	BP0935	PF03401	tricarboxylate		
Q7VXS1	BP1667	PF03401	tricarboxylate		
Q7VS39	BP0612	PF03401	tricarboxylate		
Q7VUR4	BP3012	PF03401	tricarboxylate		
Q7VSA6	BP0531	PF03401	tricarboxylate		
Q7VT49	BP3707	PF03401	tricarboxylate		
Q7VX73	BP1934	PF03401	tricarboxylate		
Q7VTH2	BP3568	PF13458	leucine/valine		
Q7VVH3	BP2692	PF00496	dipeptide/oligopeptide		
Q7W0N5	BP0063	PF03401	tricarboxylate		
Q7VZN6	BP0861	PF03401	tricarboxylate		
Q7VX39	BP1983	PF00496	dipeptide/oligopeptide		
Q7VX85	BP1920	PF03480	C4-dicarboxylate		
Q7VZT0	BP0807	PF03401	tricarboxylate		
Q7VXB3	BP1887	PF03480	C4-dicarboxylate	Pyroglutamic acid	doi: 10.1016/j.jmb.2007.04.047
Q7VUX3	BP2943	PF03401	tricarboxylate		
Q7VZ12	BP1131	PF03401	tricarboxylate		
Q7VTT3	BP3427	PF13458	urea		
Q7VS05	BP0654	PF03401	tricarboxylate	Aspartate	doi: 10.1016/j.jmb.2005.11.096
Q7VS98	BP0542	PF03401	tricarboxylate		
Q7VS10	BP0649	PF03401	tricarboxylate		
Q7VXI3	BP1780	PF03401	tricarboxylate		
Q7W0A0	BP0250	PF03401	tricarboxylate	Glutamate	doi: 10.1107/S0907444906032653
Q7VT40	BP3716	PF13458	leucine/valine		

Q7VXZ7	BP1573	PF00497	amino acid (glutamine/glutamate/aspartate?)		
Q7VS37	BP0615	PF03401	tricarboxylate		
Q7VWJ6	BP2219	PF16868	phosphonate		
Q7VYM6	BP1292	PF03180	methionine		
Q7VT55	BP3701	PF09084	glycine betaine		
Q7VTE6	BP3596	PF01497	iron-hydroxamate		
Q7VV70	BP2818	PF03180	methionine		
Q7VU86	BP3237	PF00496	dipeptide/oligopeptide		
Q7VZZ0	BP0726	PF00496	dipeptide/oligopeptide		
Q7VXA4	BP1900	PF03401	tricarboxylate		
Q7VSM1	BP0385	PF03180	methionine		
Q7VX79	BP1926	PF13416	spermidine/putrescine		
Q7VUB2	BP3196	PF13458	leucine/valine		
Q7VSQ6	BP0345	PF01497	iron-hydroxamate		
Q7VZV0	BP0782	PF09084	nitrate/sulfonate/taurine		
Q7W074	BP0284	PF00496	dipeptide/oligopeptide		
Q7VSH0	BP0454	PF16868	phosphonate		
Q7VYN1	BP1285	PF13458	leucine/valine		
Q7VWV1	BP2090	PF13458	leucine/valine		
Q7VZ42	BP1097	PF13458	urea		
Q7VWB2	BP2348	PF13416	spermidine/putrescine		
Q7VSW7	BP3802	PF00496	dipeptide/oligopeptide		
Q7VSS3	BP3855	PF09084	nitrate/sulfonate/taurine		
Q7VW70	BP2396	PF00496	dipeptide/oligopeptide		
Q7VWS8	BP2116	PF03401	tricarboxylate		
Q7VUK6	BP3080	PF01297	manganese/zinc ion		
Q7VS83	BP0558	PF00497	amino acid (glutamine/glutamate/aspartate?)		
Q7VWX9	BP2055	PF04069	glycine betaine		
Q7VS30	BP0622	PF13458	leucine/valine	3-hydroxybenzoic acid	PDB: 4Q6W
Q7VWW8	BP2068	PF04392	Unclassified		

Q7VYW9	BP1184	PF00497	amino acid (glutamine/glutamate/aspartate?)		
Q7VT52	BP3704	PF09084	glycine betaine		
Q7VXC7	BP1852	PF00497	amino acid (glutamine/glutamate/aspartate?)		
Q7VXR5	BP1673	PF03401	tricarboxylate		
Q7VSU1	BP3831	PF00497	amino acid (glutamine/glutamate/aspartate?)		
Q7VY34	BP1529	PF00497	amino acid (glutamine/glutamate/aspartate?)		
Q7VTG5	BP3575	PF13458	urea		
Q7VSR1	BP3867	PF03401	tricarboxylate		
***Pseudomonas fluorescens* Pf0-1**					
Number of ORFs in the proteome: 5712					
Number of SBPs: 94 (1.65% of total)					
Lifestyle Summary: SOIL					
**SeqID**	**Gene Locus**	**Pfam Code**	**Ligand prediction by TransportDB**	**Experimentally identified ligands**	**References/pdb accession codes**
Q3K987	Pfl01_3930	PF01297	manganese/zinc ion		
Q3KEJ9	Pfl01_2064	PF00497	amino acid (glutamine/glutamate/aspartate?)		
Q3K8M6	Pfl01_4141	PF00532	ribose		
Q3K5C8	Pfl01_5289	PF00497	amino acid (glutamine/glutamate/aspartate?)		
Q3KHP0	Pfl01_0973	PF00497	amino acid (glutamine/glutamate/aspartate?)		
Q3KKD7	Pfl01_0025	PF04069	choline		
Q3K6R7	Pfl01_4800	PF01497	iron-hydroxamate		
Q3KB22	Pfl01_3294	PF09084	nitrate/sulfonate/taurine		
Q3KI47	Pfl01_0816	PF00496	dipeptide/oligopeptide		
Q3K974	Pfl01_3943	PF00497	amino acid (glutamine/glutamate/aspartate?)		

Q3K4D6	Pfl01_5632	PF13416	spermidine/putrescine		
Q3K9S5	Pfl01_3741	PF00496	oligopeptide		
Q3K4M9	Pfl01_5538	PF00496	dipeptide/oligopeptide		
Q3K4F2	Pfl01_5615	PF12849	phosphate		
Q3K658	Pfl01_5009	PF01497	iron-hydroxamate		
Q3KE81	Pfl01_2182	PF00496	oligopeptide		
Q3KCG2	Pfl01_2802	PF00497	amino acid (glutamine/glutamate/aspartate?)		
Q3KEZ4	Pfl01_1919	PF13407	rhamnose		
Q3K5I7	Pfl01_5230	PF04069	choline		
Q3K4A7	Pfl01_5661	PF01297	manganese/zinc ion		
Q3K503	Pfl01_5414	PF09084	glycine betaine		
Q3KBP3	Pfl01_3073	PF00496	oligopeptide		
Q3K528	Pfl01_5389	PF00497	amino acid (glutamine/glutamate/aspartate?)		
Q3KB75	Pfl01_3241	PF13416	spermidine/putrescine		
Q3KJF0	Pfl01_0362	PF04069	glycine betaine		
Q3KJD7	Pfl01_0375	PF00497	amino acid (glutamine/glutamate/aspartate?)		
Q3KJE3	Pfl01_0369	PF04069	choline		
Q3K7I2	Pfl01_4535	PF00497	amino acid (glutamine/glutamate/aspartate?)		
Q3KAL2	Pfl01_3454	PF03480	C4-dicarboxylate		
Q3K733	Pfl01_4684	PF04348	leucine/valine		
Q3KDS3	Pfl01_2340	PF13407	rhamnose		
Q3KGW5	Pfl01_1248	PF13458	leucine/valine		
Q3KD39	Pfl01_2575	PF13416	spermidine/putrescine		
Q3K9K2	Pfl01_3814	PF00497	amino acid (glutamine/glutamate/aspartate?)		
Q3K513	Pfl01_5404	PF13416	spermidine/putrescine		
Q3K8Q2	Pfl01_4115	PF00497	amino acid (glutamine/glutamate/aspartate?)		
Q3KCX4	Pfl01_2640	PF01547	sugar		

Q3KAZ7	Pfl01_3319	PF04069	glycine betaine		
Q3K4Z9	Pfl01_5418	PF09084	glycine betaine		
Q3KFV7	Pfl01_1606	PF04069	glycine betaine		
Q3K5D5	Pfl01_5282	PF00497	amino acid (glutamine/glutamate/aspartate?)		
Q3KH03	Pfl01_1210	PF00497	amino acid (glutamine/glutamate/aspartate?)		
Q3K823	Pfl01_4344	PF04069	glycine betaine		
Q3KEE6	Pfl01_2117	PF13416	spermidine/putrescine		
Q3KGM0	Pfl01_1343	PF09084	nitrate/sulfonate/taurine		
Q3KBE4	Pfl01_3172	PF12849	phosphate		
Q3KDW1	Pfl01_2302	PF13407	xylose		
Q3KE82	Pfl01_2181	PF00496	oligopeptide		
Q3KCI4	Pfl01_2780	PF04069	glycine betaine		
Q3KEA8	Pfl01_2155	PF13416	spermidine/putrescine		
Q3KBK6	Pfl01_3110	PF13407	ribose		
Q3KBH1	Pfl01_3145	PF13416	spermidine/putrescine		
Q3K4V8	Pfl01_5459	PF00497	amino acid (glutamine/glutamate/aspartate?)		
Q3KBJ6	Pfl01_3120	PF00496	oligopeptide		
Q3KJV1	Pfl01_0211	PF09084	phosphonates		
Q3KKA3	Pfl01_0059	PF01297	zinc ion		
Q3KB23	Pfl01_3293	PF09084	glycine betaine		
Q3K5U3	Pfl01_5124	PF13416	spermidine/putrescine		
Q3KI50	Pfl01_0813	PF00496	dipeptide/oligopeptide		
Q3KJI4	Pfl01_0328	PF00497	amino acid (glutamine/glutamate/aspartate?)		
Q3KCD5	Pfl01_2829	PF13416	spermidine/putrescine		
Q3KII9	Pfl01_0673	PF02608	sugar		
Q3KJQ6	Pfl01_0256	PF09084	glycine betaine		
Q3KJT9	Pfl01_0223	PF03180	methionine		
Q3KCT7	Pfl01_2677	PF13416	spermidine/putrescine		
Q3KB56	Pfl01_3260	PF13416	spermidine/putrescine		

Q3KJS5	Pfl01_0237	PF03180	methionine		
Q3KIW0	Pfl01_0552	PF13458	leucine/valine		
Q3KI48	Pfl01_0815	PF00496	dipeptide/oligopeptide		
Q3KBM4	Pfl01_3092	PF00497	amino acid (glutamine/glutamate/aspartate?)		
Q3KFA9	Pfl01_1804	PF00497	Unclassified		
Q3KHP6	Pfl01_0967	PF00497	amino acid (glutamine/glutamate/aspartate?)		
Q3K7P8	Pfl01_4469	PF13416	spermidine/putrescine		
Q3KHG5	Pfl01_1048	PF13416	spermidine/putrescine		
Q3K5G6	Pfl01_5251	PF00497	amino acid (glutamine/glutamate/aspartate?)		
Q3K990	Pfl01_3927	PF01297	manganese/zinc ion		
Q3KCA9	Pfl01_2855	PF00497	amino acid (glutamine/glutamate/aspartate?)		
Q3KDP1	Pfl01_2372	PF13416	spermidine/putrescine		
Q3K512	Pfl01_5405	PF13416	spermidine/putrescine		
Q3KB11	Pfl01_3305	PF00497	amino acid (glutamine/glutamate/aspartate?)		
Q3KJK1	Pfl01_0311	PF00497	amino acid (glutamine/glutamate/aspartate?)		
Q3K655	Pfl01_5012	PF00497	amino acid (glutamine/glutamate/aspartate?)		
Q3K5H9	Pfl01_5238	PF04069	choline		
Q3KDS8	Pfl01_2335	PF03180	methionine		
Q3KJT4	Pfl01_0228	PF00497	amino acid (glutamine/glutamate/aspartate?)		
Q3KK95	Pfl01_0067	PF03180	methionine		
Q3K6D6	Pfl01_4931	PF09084	nitrate/sulfonate/taurine		
Q3K875	Pfl01_4292	PF00497	amino acid (glutamine/glutamate/aspartate?)		
Q3KGL0	Pfl01_1353	PF03401	tricarboxylate		
Q3K7Z7	Pfl01_4370	PF13416	N-acetylglucosamine		

Q3KJR8	Pfl01_0244	PF00497	amino acid (glutamine/glutamate/aspartate?)		
Q3KIS0	Pfl01_0592	PF01497	cobalamin		
Q3KAD4	Pfl01_3532	PF13416	spermidine/putrescine		
Q3KI58	Pfl01_0805	PF04069	glycine betaine		
***Brucella abortus* 2308**					
Number of ORFs in the proteome: 3023					
Number of SBPs: 62 (2.05% of total)					
Lifestyle Summary: HUMAN/ANIMAL PATHOGEN					
**SeqID**	**Gene Locus**	**Pfam Code**	**Ligand prediction by TransportDB**	**Experimentally identified ligands**	**References/pdb accession codes**
Q2YKR5	BAB2_0585	PF13416	sugar		
Q2YJB6	BAB2_1146	PF09084	phosphonates		
Q2YLW8	BAB1_1770	PF01547	thiamin		
Q2YKJ5	BAB2_0664	PF00496	oligopeptide		
Q2YL86	BAB2_0023	PF13458	urea		
Q2YLG0	BAB1_1792	PF13458	leucine/valine		
Q2YLF8	BAB1_1794	PF13458	leucine/valine		
Q2YK58	BAB2_0822	PF13458	leucine/valine		
Q2YKZ9	BAB2_0491	PF01547	sugar		
Q2YJK2	BAB2_1049	PF00496	dipeptide/oligopeptide		
Q2YK66	BAB2_0812	PF00496	dipeptide/oligopeptide		
Q2YJH5	BAB2_1079	PF01297	zinc ion	Zinc	doi: 10.1128/IAI.01957-05
Q2YK50	BAB2_0830	PF13458	leucine/valine		
Q2YKI6	BAB2_0673	PF02608	sugar	Adenine	PDB: 3S99
Q2YKQ9	BAB2_0593	PF13458	urea		
Q2YJA9	BAB2_1153	PF13458	leucine/valine		
Q2YL39	BAB2_0451	PF00496	dipeptide/oligopeptide		
Q2YIY2	BAB2_0377	PF13407	xylose		
Q2YRY0	BAB1_1593	PF04069	choline		
Q2YJW3	BAB2_0927	PF05048	Not annotated in TransportDB		
Q2YKZ2	BAB2_0502	PF04069	glycine betaine		

Q2YQQ6	BAB1_2176	PF03180	methionine		
Q2YKU0	BAB2_0558	PF00497	amino acid (glutamine/glutamate/aspartate?)		
Q2YJ40	BAB2_0427	PF13416	thiamin		
Q2YK03	BAB2_0879	PF13416	spermidine/putrescine		
Q2YJV3	BAB2_0938	PF13407	xylose		
Q2YJS2	BAB2_0974	PF00496	dipeptide/oligopeptide		
Q2YP76	BAB1_0226	PF04069	glycine betaine	Glycine-betaine, choline, ectoine, proline, carnitine (low affinity)	doi: 10.1128/JB.00746-16
Q2YQM0	BAB1_1313	PF13458	leucine/valine		
Q2YK97	BAB2_0767	PF09084	nitrate/sulfonate/taurine		
Q2YP60	BAB1_0214	PF09084	nitrate/sulfonate/taurine		
Q2YLB9	BAB2_0282	PF13458	urea		
Q2YMQ4	BAB1_0567	PF13407	xylose		
Q2YJK7	BAB2_1043	PF00496	dipeptide/oligopeptide		
Q2YKT4	BAB2_0564	PF01497	iron-hydroxamate		
Q2YRX3	BAB1_1600	PF00496	dipeptide/oligopeptide		
Q2YNH7	BAB1_0881	PF00497	amino acid (glutamine/glutamate/aspartate?)		
Q2YJI9	BAB2_1062	PF13416	spermidine/putrescine		
Q2YQD1	BAB1_1366	PF01497	iron-hydroxamate		
Q2YJJ6	BAB2_1055	PF00496	dipeptide/oligopeptide		
Q2YLW5	BAB1_1954	PF00497	amino acid (glutamine/glutamate/aspartate?)		
Q2YQA1	BAB1_1628	PF13416	spermidine/putrescine		
Q2YLB5	BAB2_0286	PF00497	amino acid (glutamine/glutamate/aspartate?)		
Q2YKP5	BAB2_0611	PF00497	amino acid (glutamine/glutamate/aspartate?)		
Q2YRV5	BAB1_1216	PF16868	glycine betaine		
Q2YKG0	BAB2_0700	PF00496	oligopeptide		

Q2YQB8	BAB1_1648	PF13407	xylose		
Q2YLA0	BAB2_0009	PF02608	sugar		
Q2YL07	BAB2_0483	PF01497	cobalamin		
Q2YIV4	BAB2_0275	PF03180	methionine		
Q2YJE8	BAB2_1109	PF13407	xylose		
Q2YKG1	BAB2_0699	PF00496	oligopeptide		
Q2YPL6	BAB1_0010	PF00496	oligopeptide		
Q2YQU1	BAB1_2141	PF12849	phosphate		
Q2YJC7	BAB2_1132	PF13458	urea		
Q2YPE4	BAB1_0325	PF00497	amino acid (glutamine/glutamate/aspartate?)		
Q2YPL7	BAB1_0009	PF00496	oligopeptide		
Q2YQE3	BAB1_1362	PF13407	ribose		
Q2YPK1	BAB1_0373	PF03480	C4-dicarboxylate		
Q2YKU9	BAB2_0547	PF01547	sugar		
Q2YKP4	BAB2_0612	PF00497	amino acid (glutamine/glutamate/aspartate?)		
Q2YR20	BAB1_2054	PF04392	Unclassified		
***Acinetobacter baumannii* AYE**					
Number of ORFs in the proteome: 3652					
Number of SBPs: 18 (0.49% of total)					
Lifestyle Summary: HUMAN/ANIMAL PATHOGEN					
**SeqID**	**Gene Locus**	**Pfam Code**	**Ligand prediction by TransportDB**	**Experimentally identified ligands**	**References/pdb accession codes**
B0VBK4	ABAYE2253	PF00497	amino acid (glutamine/glutamate/aspartate?)		
B0VBK5	ABAYE2254	PF00497	amino acid (glutamine/glutamate/aspartate?)		
B0VC68	ABAYE2161	PF00497	amino acid (glutamine/glutamate/aspartate?)		
B0VBM8	ABAYE3725	PF01297	zinc ion		
B0VAB4	ABAYE2662	PF00496	oligopeptide		

B0VC25	ABAYE2212	PF04069	glycine betaine		
B0VAD5	ABAYE2638	PF03180	methionine		
B0V5V7	ABAYE3840	PF09084	glycine betaine		
B0V7E3	ABAYE1409	PF09084	nitrate/sulfonate/taurine		
B0VBB8	ABAYE1177	PF00497	amino acid (glutamine/glutamate/aspartate?)		
B0VC73	ABAYE2167	PF03180	methionine		
B0VBU2	ABAYE1092	PF01497	iron-hydroxamate		
B0VC74	ABAYE2168	PF03180	methionine		
B0VB10	ABAYE2421	PF09084	nitrate/sulfonate/taurine		
B0VD28	ABAYE2044	PF00496	dipeptide/oligopeptide		
B0VBZ9	ABAYE1032	PF12849	phosphate		
B0V7H3	ABAYE1365	PF03180	methionine		
B0VD29	ABAYE2045	PF00496	dipeptide/oligopeptide		
***Nocardia brasiliensis* ATCC 700358**					
Number of ORFs in the proteome: 8414					
Number of SBPs: 80 (0.95% of total)					
Lifestyle Summary: HUMAN/ANIMAL PATHOGEN					
**SeqID**	**Gene Locus**	**Pfam Code**	**Ligand prediction by TransportDB**	**Experimentally identified ligands**	**References/pdb accession codes**
K0EFU5	O3I_000600	PF00496	dipeptide/oligopeptide		
K0EN58	O3I_001835	PF00496	oligopeptide		
K0F7Q8	O3I_027885	PF00497	amino acid (glutamine/glutamate/aspartate?)		
K0F6L6	O3I_035340	PF13458	urea		
K0ENN9	O3I_003230	PF00496	dipeptide/oligopeptide		
K0F0Z0	O3I_016925	PF09084	nitrate/sulfonate/taurine		
K0EJ32	O3I_007000	PF13407	xylose		
K0ETD2	O3I_004790	PF16868	Unclassified		
K0EVJ2	O3I_015740	PF01297	zinc		
K0ENA3	O3I_002505	PF01297	zinc		
K0EV74	O3I_017505	PF13407	xylose		

K0F0B2	O3I_023250	PF00496	dipeptide/oligopeptide		
K0FC11	O3I_035645	PF04069	glycine betaine		
K0F8J3	O3I_029135	PF01547	sugar		
K0ETM2	O3I_012440	PF00497	amino acid (glutamine/glutamate/aspartate?)		
K0ET74	O3I_013580	PF13407	ribose		
K0EXB1	O3I_032845	PF02608	sugar		
K0EMP7	O3I_014510	PF01547	sugar		
K0EUX5	O3I_014740	PF13407	ribose		
K0F1C6	O3I_027665	PF03180	methionine		
K0ELI1	O3I_012060	PF01497	cobalamin		
K0EWS9	O3I_016620	PF13416	sugar		
K0EYB9	O3I_022760	PF01497	Gene locus not annotated in Uniprot		
K0EXK5	O3I_011625	PF00497	amino acid (glutamine/glutamate/aspartate?)		
K0EH37	O3I_003150	PF13458	urea		
K0F4D2	O3I_033040	PF01497	iron-hydroxamate		
K0ESD0	O3I_012080	PF09084	nitrate/sulfonate/taurine		
K0EUL3	O3I_027670	PF03180	methionine		
K0EV70	O3I_017455	PF16868	phosphonates		
K0ET66	O3I_004315	PF01497	iron-hydroxamate		
K0ETA3	O3I_011615	PF00496	dipeptide/oligopeptide		
K0EP46	O3I_017460	PF16868	phosphonates		
K0EQS4	O3I_020995	PF01497	iron-hydroxamate		
K0F7A5	O3I_026935	PF09084	nitrate/sulfonate/taurine		
K0EYI7	O3I_013200	PF00496	dipeptide/oligopeptide		
K0EPB9	O3I_004380	PF01497	iron-hydroxamate		
K0F5A7	O3I_032730	PF00496	oligopeptide		
K0EWS5	O3I_010550	PF01547	sugar		
K0F6F8	O3I_025400	PF01497	iron-hydroxamate		
K0EZS6	O3I_023245	PF00496	dipeptide/oligopeptide		
K0F6N5	O3I_036885	PF00496	dipeptide/oligopeptide		
K0F4S5	O3I_033590	PF00496	oligopeptide		

K0F284	O3I_018885	PF00496	dipeptide/oligopeptide		
K0F3R8	O3I_032165	PF01497	iron-hydroxamate		
K0EM50	O3I_000605	PF00496	dipeptide/oligopeptide		
K0EY51	O3I_012800	PF00497	amino acid (glutamine/glutamate/aspartate?)		
K0F598	O3I_034390	PF01497	cobalamin		
K0F4C9	O3I_022305	PF13407	rhamnose		
K0FC69	O3I_036020	PF04069	glycine betaine		
K0F002	O3I_025480	PF01547	sugar		
K0F9A7	O3I_040050	PF00497	amino acid (glutamine/glutamate/aspartate?)		
K0EJ14	O3I_006850	PF13407	xylose		
K0EV66	O3I_007540	PF00496	dipeptide/oligopeptide		
K0F2E6	O3I_041990	PF13416	sugar		
K0EWW3	O3I_031820	PF13407	rhamnose		
K0F1Y4	O3I_028790	PF00496	dipeptide/oligopeptide		
K0EYH6	O3I_022985	PF09084	nitrate/sulfonate/taurine		
K0F384	O3I_029975	PF00497	amino acid (glutamine/glutamate/aspartate?)		
K0F521	O3I_034015	PF00496	oligopeptide		
K0EUA6	O3I_012520	PF13458	leucine/valine		
K0F2T2	O3I_027505	PF01547	sugar		
K0F0S0	O3I_025415	PF01497	iron-hydroxamate		
K0EUL8	O3I_013195	PF00496	dipeptide/oligopeptide		
K0ETM0	O3I_025945	PF09084	nitrate/sulfonate/taurine		
K0F4P3	O3I_033175	PF00496	dipeptide/oligopeptide		
K0EH75	O3I_003350	PF12849	phosphate		
K0F1Y0	O3I_041240	PF01547	sugar		
K0EUI4	O3I_013840	PF01547	sugar		
K0F6N8	O3I_025885	PF00497	amino acid (glutamine/glutamate/aspartate?)		
K0EV98	O3I_015315	PF13407	ribose		
K0ERV9	O3I_022965	PF09084	nitrate/sulfonate/taurine		

K0EYV0	O3I_021325	PF04069	glycine betaine		
K0F7X1	O3I_039360	PF13416	sugar		
K0F5X5	O3I_035950	PF00496	dipeptide/oligopeptide		
K0F4X5	O3I_023180	PF00496	dipeptide/oligopeptide		
K0EXX3	O3I_021860	PF13458	leucine/valine		
K0ELW1	O3I_012885	PF00496	dipeptide/oligopeptide		
K0F5R4	O3I_035650	PF04069	glycine betaine		
K0EZL0	O3I_037305	PF13407	xylose		
K0F7V6	O3I_039260	PF09084	nitrate/sulfonate/taurine		
***Lactobacillus acidophilus* NCFM**					
Number of ORFs in the proteome: 1859					
Number of SBPs: 29 (1.56% of total)					
Lifestyle Summary: HUMAN INTESTINAL MICROFLORA					
**SeqID**	**Gene Locus**	**Pfam Code**	**Ligand prediction by TransportDB**	**Experimentally identified ligands**	**References/pdb accession codes**
Q5FKL3	LBA0904	PF03180	methionine		
Q5FI08	LBA1866	PF13416	sugar (maltose?)		
G1UB54	LBA1442	PF01547	sugar		
Q5FHS2	LBA1958	PF00496	oligopeptide		
Q5FJJ3	LBA1301	PF00496	dipeptide/oligopeptide		
Q5FIJ0	LBA1672	PF00497	amino acid (glutamine/glutamate/aspartate?)		
Q5FK82	LBA1046	PF00497	amino acid (glutamine/glutamate/aspartate?)		
Q5FJ99	LBA1400	PF00496	dipeptide/oligopeptide		
Q5FJ23	LBA1481	PF13407	xylose		
Q5FI94	LBA1773	PF01297	manganese/zinc ion		
Q5FJJ4	LBA1300	PF00496	dipeptide/oligopeptide		
Q5FLF9	LBA0585	PF13416	sugar		
Q5FJA4	LBA1395	PF03180	methionine		
G1UB60	LBA0502	PF13416	sugar		
Q5FHT6	LBA1943	PF02608	sugar		

Q5FIJ7	LBA1665	PF00496	oligopeptide		
Q5FHT7	LBA1942	PF02608	sugar		
Q5FJS3	LBA1216	PF00496	oligopeptide		
Q5FMI2	LBA0197	PF00496	oligopeptide		
Q5FLG4	LBA0579	PF00497	amino acid (glutamine/glutamate/aspartate?)		
Q5FJF1	LBA1346	PF00496	oligopeptide		
Q5FIM1	LBA1641	PF13416	sugar		
Q5FLH1	LBA0572	PF00497	amino acid (glutamine/glutamate/aspartate?)		
Q5FM21	LBA0361	PF12849	phosphate		
Q5FHR9	LBA1961	PF00496	oligopeptide		
Q5FMX1	LBA0049	PF04392	Unclassified		
Q5FL38	LBA0713	PF13416	spermidine/putrescine		
Q5FMI1	LBA0198	PF00496	oligopeptide		
Q5FJF0	LBA1347	PF00496	oligopeptide		
***Spirochaeta thermophila* Z-1203**					
Number of ORFs in the proteome: 2249					
Number of SBPs: 41 (1.82% of total)					
Lifestyle Summary: FRESH/MARINE WATERS					
**SeqID**	**Gene Locus**	**Pfam Code**	**Ligand prediction by TransportDB**	**Experimentally identified ligands**	**References/pdb accession codes**
G0GDU0	Spith_1962	PF09084	nitrate/sulfonate/taurine		
G0GDC0	Spith_0260	PF00496	oligopeptide		
G0GFS0	Spith_1349	PF13416	sugar		
G0GAA3	Spith_0660	PF01547	sugar		
G0GC43	Spith_0120	PF13458	leucine/valine		
G0GEZ5	Spith_2083	PF03480	C4-dicarboxylate		
G0GB12	Spith_0760	PF00496	dipeptide/oligopeptide		
G0GAT4	Spith_1569	PF01547	sugar		
G0GF74	Spith_0493	PF04069	glycine betaine		
G0GDT6	Spith_1958	PF02608	sugar		

G0GA73	Spith_0629	PF03401	tricarboxylate		
G0GDR0	Spith_1932	PF13416	sugar		
G0GFK0	Spith_2144	PF03480	C4-dicarboxylate		
G0GF11	Spith_2099	PF13407	rhamnose		
G0GDA3	Spith_0243	PF01547	sugar		
G0GEJ2	Spith_0396	PF01547	sugar		
G0GBA3	Spith_1651	PF03480	C4-dicarboxylate		
G0GA25	Spith_1449	PF01547	sugar		
G0GDW1	Spith_0307	PF13407	xylose		
G0GAH2	Spith_1529	PF01547	sugar		
G0GEM1	Spith_0425	PF13407	rhamnose		
G0GBZ2	Spith_1743	PF01547	sugar		
G0GAD1	Spith_1487	PF03480	C4-dicarboxylate		
G0GDR9	Spith_1941	PF00496	oligopeptide		
G0GF15	Spith_2103	PF01547	sugar		
G0GFK9	Spith_2153	PF01497	iron-hydroxamate		
G0GDT1	Spith_1953	PF13458	leucine/valine		
G0GD79	Spith_1896	PF13416	sugar		
G0GA78	Spith_0634	PF01297	zinc		
G0GFH4	Spith_1323	PF01547	sugar		
G0GA71	Spith_0627	PF03480	C4-dicarboxylate		
G0GFR0	Spith_1339	PF13407	rhamnose		
G0GD21	Spith_1041	PF13416	sugar (maltose?)		
G0GEI1	Spith_0384	PF03480	C4-dicarboxylate		
G0G9T2	Spith_0552	PF12849	phosphate		
G0GCJ3	Spith_1800	PF00496	dipeptide/oligopeptide		
G0GF63	Spith_0482	PF13416	sugar		
G0GE23	Spith_0370	PF01547	sugar		
G0GAN1	Spith_0717	PF01547	sugar		
G0GBK3	Spith_0075	PF01547	sugar		
G0GD29	Spith_1845	PF02608	sugar		
***Azospirillum brasilense* Sp245**					

Number of ORFs in the proteome: 7541					
Number of SBPs: 161 (2.13% of total)					
Lifestyle Summary: BENEFICIAL PLANT ASSOCIATED					
**SeqID**	**Gene Locus**	**Pfam Code**	**Ligand prediction by TransportDB**	**Experimentally identified ligands**	**References/pdb accession codes**
G8AWV9	AZOBR_p310110	PF13458	leucine/valine		
G8AKQ3	AZOBR_100201	PF02608	sugar		
G8ATS3	AZOBR_p1130057	PF03480	C4-dicarboxylate		
G8AQ61	AZOBR_p110048	PF00496	oligopeptide		
G8AUW6	AZOBR_p230066	PF00496	dipeptide/oligopeptide		
G8AX81	AZOBR_p310232	PF13416	2-aminoethylphosphonate		
G8AFZ2	AZOBR_180217	PF13458	leucine/valine		
G8AYT0	AZOBR_p410007	PF13458	leucine/valine		
G8B019	AZOBR_p460039	PF03480	C4-dicarboxylate		
G8AGP3	AZOBR_180300	PF09084	nitrate/sulfonate/taurine		
G8AQZ2	AZOBR_p130041	PF04069	glycine betaine		
G8AZU1	AZOBR_p440163	PF13407	xylose		
G8AYW5	AZOBR_p410042	PF01497	iron-hydroxamate		
G8AWS4	AZOBR_p310075	PF00497	amino acid (glutamine/glutamate/aspartate?)		
G8AVY5	AZOBR_p270290	PF03480	C4-dicarboxylate		
G8ATU5	AZOBR_p210018	PF13458	urea		
G8AIN1	AZOBR_40262	PF00496	oligopeptide		
G8AR13	AZOBR_p130062	PF04392	Unclassified		
G8AEG0	AZOBR_10106	PF00497	amino acid (glutamine/glutamate/aspartate?)		
G8AGU1	AZOBR_190023	PF13458	leucine/valine		
G8AXV7	AZOBR_p330117	PF01297	zinc		
G8AZX8	AZOBR_p450012	PF03480	C4-dicarboxylate		
G8AQV9	AZOBR_p130008	PF13458	leucine/valine		
G8AFF2	AZOBR_10288	PF00496	oligopeptide		
G8ASH5	AZOBR_p170142	PF09084	glycine betaine		
G8AXB6	AZOBR_p310267	PF09084	nitrate/sulfonate/taurine		

G8AXU2	AZOBR_p330102	PF01547	sugar		
G8AF88	AZOBR_10224	PF01297	zinc		
G8AX46	AZOBR_p310197	PF13458	urea		
G8AVD6	AZOBR_p270091	PF13458	leucine/valine		
G8AXK5	AZOBR_p330015	PF09084	nitrate/sulfonate/taurine		
G8AUJ9	AZOBR_p220071	PF03480	C4-dicarboxylate		
G8AWU7	AZOBR_p310098	PF13458	leucine/valine		
G8AWX6	AZOBR_p310127	PF03480	C4-dicarboxylate		
G8ALR6	AZOBR_120022	PF16868	phosphonates		
G8AWG2	AZOBR_p350061	PF00496	dipeptide/oligopeptide		
G8AX64	AZOBR_p310215	PF13458	urea		
G8B0K8	AZOBR_p480032	PF13458	leucine/valine		
G8AXQ9	AZOBR_p330069	PF13416	2-aminoethylphosphonate		
G8AFE0	AZOBR_10276	PF13416	spermidine/putrescine		
G8AZA8	AZOBR_p430046	PF00496	dipeptide/oligopeptide		
G8AVV9	AZOBR_p270264	PF00496	dipeptide/oligopeptide		
G8AH75	AZOBR_30007	PF00496	dipeptide/oligopeptide		
G8AJB8	AZOBR_50054	PF00496	dipeptide/oligopeptide		
G8B005	AZOBR_p460025	PF00496	dipeptide/oligopeptide		
G8AVY9	AZOBR_p270294	PF03180	methionine		
G8AJE0	AZOBR_60013	PF13458	urea		
G8B091	AZOBR_p470016	PF02608	sugar		
G8AUA9	AZOBR_p210182	PF00496	dipeptide/oligopeptide		
G8AZT6	AZOBR_p440158	PF13407	ribose		
G8AET0	AZOBR_160063	PF09084	phosphonates		
G8AZI8	AZOBR_p440060	PF13416	spermidine/putrescine		
G8AGD9	AZOBR_10461	PF13458	leucine/valine		
G8AXS9	AZOBR_p330089	PF13407	xylose		
G8B0I0	AZOBR_p480004	PF13458	urea		
G8AGP9	AZOBR_180306	PF09084	nitrate/sulfonate/taurine		
G8AFY1	AZOBR_180206	PF09084	nitrate/sulfonate/taurine		
G8AED5	AZOBR_10081	PF09084	nitrate/sulfonate/taurine		
G8AZQ0	AZOBR_p440122	PF02608	sugar		

G8AY75	AZOBR_p340072	PF13458	leucine/valine		
G8AY06	AZOBR_p340003	PF05048	Not annotated in TransportDB		
G8AYS1	AZOBR_p350046	PF01497	iron-hydroxamate		
G8AWP9	AZOBR_p310050	PF13416	2-aminoethylphosphonate		
G8AG29	AZOBR_10351	PF01297	zinc		
G8AE60	AZOBR_10006	PF13416	sugar		
G8ASZ6	AZOBR_p1110021	PF09084	phosphonates		
G8AKI8	AZOBR_100136	PF13458	leucine/valine		
G8ARP0	AZOBR_p140067	PF13458	leucine/valine		
G8AX92	AZOBR_p310243	PF00497	amino acid (glutamine/glutamate/aspartate?)		
G8AVW3	AZOBR_p270268	PF09084	nitrate/sulfonate/taurine		
G8AUH6	AZOBR_p220048	PF03401	tricarboxylate		
G8AEU5	AZOBR_160078	PF09084	nitrate/sulfonate/taurine		
G8ATJ3	AZOBR_p1120052	PF13416	spermidine/putrescine		
G8AGA7	AZOBR_10429	PF00496	dipeptide/oligopeptide		
G8AW03	AZOBR_p270308	PF13458	urea		
G8AGF3	AZOBR_10475	PF13458	leucine/valine		
G8ASM7	AZOBR_p1100004	PF00497	amino acid (glutamine/glutamate/aspartate?)		
G8AMV1	AZOBR_140293	PF00497	amino acid (glutamine/glutamate/aspartate?)		
G8ATF2	AZOBR_p1120011	PF03180	methionine		
G8AIN2	AZOBR_40263	PF00496	oligopeptide		
G8AHZ4	AZOBR_40066	PF13458	branched-chain amino acid		
G8ATA1	AZOBR_p1110126	PF03480	C4-dicarboxylate		
G8AWZ6	AZOBR_p310147	PF00496	dipeptide/oligopeptide		
G8ASL0	AZOBR_p180016	PF01547	sugar		
G8AH04	AZOBR_10510	PF00496	dipeptide/oligopeptide		
G8AYR7	AZOBR_p350042	PF09084	phosphonates		
G8AME3	AZOBR_140135	PF00497	Unclassified		
G8ATF7	AZOBR_p1120016	PF03180	methionine		
G8AP88	AZOBR_p1140076	PF13458	urea		

G8AUJ5	AZOBR_p220067	PF04392	trimethylamine N-oxide		
G8AWN9	AZOBR_p310040	PF00496	dipeptide/oligopeptide		
G8ALX7	AZOBR_130017	PF13416	spermidine/putrescine		
G8AEZ0	AZOBR_180033	PF00497	amino acid (glutamine/glutamate/aspartate?)		
G8AFB0	AZOBR_10246	PF12849	phosphate		
G8AME8	AZOBR_140140	PF03480	C4-dicarboxylate		
G8AQZ6	AZOBR_p130045	PF13416	spermidine/putrescine		
G8AQX3	AZOBR_p130022	PF00497	amino acid (glutamine/glutamate/aspartate?)		
G8AIV8	AZOBR_40339	PF13458	urea		
G8AL55	AZOBR_100353	PF09084	nitrate/sulfonate/taurine		
G8AYL9	AZOBR_p340216	PF13458	urea		
G8AJB5	AZOBR_50051	PF00497	amino acid (glutamine/glutamate/aspartate?)		
G8AVV0	AZOBR_p270255	PF03401	tricarboxylate		
G8AXA0	AZOBR_p310251	PF03480	C4-dicarboxylate		
G8AH97	AZOBR_30029	PF03480	C4-dicarboxylate		
G8AZ35	AZOBR_p420006	PF03480	C4-dicarboxylate		
G8AXV4	AZOBR_p330114	PF01297	zinc		
G8AWT2	AZOBR_p310083	PF03480	C4-dicarboxylate		
G8AUC1	AZOBR_p210194	PF03480	C4-dicarboxylate		
G8ATP0	AZOBR_p1130024	PF03480	C4-dicarboxylate		
G8AQ83	AZOBR_p110070	PF03480	C4-dicarboxylate		
G8AQ63	AZOBR_p110050	PF03480	C4-dicarboxylate		
G8AU30	AZOBR_p210103	PF00497	amino acid (glutamine/glutamate/aspartate?)		
G8AUV1	AZOBR_p230051	PF03480	C4-dicarboxylate		
G8B0L8	AZOBR_p480042	PF03401	tricarboxylate		
G8AU43	AZOBR_p210116	PF03401	tricarboxylate		
G8AU42	AZOBR_p210115	PF03401	tricarboxylate		
G8AE82	AZOBR_10028	PF03401	tricarboxylate		
G8AJ65	AZOBR_50001	PF03480	C4-dicarboxylate		

G8AXL6	AZOBR_p330026	PF09084	nitrate/sulfonate/taurine		
G8AUH7	AZOBR_p220049	PF03401	tricarboxylate		
G8AM55	AZOBR_140047	PF00497	amino acid (glutamine/glutamate/aspartate?)		
G8AKE7	AZOBR_100095	PF04392	Unclassified		
G8AE80	AZOBR_10026	PF03401	tricarboxylate		
G8ASG1	AZOBR_p170128	PF01497	iron-hydroxamate		
G8AGN3	AZOBR_180290	PF09084	nitrate/sulfonate/taurine		
G8AVU2	AZOBR_p270247	PF09084	nitrate/sulfonate/taurine		
G8AKH6	AZOBR_100124	PF02608	sugar		
G8AUI6	AZOBR_p220058	PF01497	iron-hydroxamate		
G8AWJ7	AZOBR_p360005	PF00497	amino acid (glutamine/glutamate/aspartate?)		
G8AZR0	AZOBR_p440132	PF02608	sugar		
G8AV11	AZOBR_p250011	PF13416	spermidine/putrescine		
G8ARK0	AZOBR_p140027	PF13458	urea		
G8AEJ5	AZOBR_10141	PF00497	amino acid (glutamine/glutamate/aspartate?)		
G8AEQ6	AZOBR_160039	PF13458	urea		
G8AX77	AZOBR_p310228	PF00497	amino acid (glutamine/glutamate/aspartate?)		
G8AZP1	AZOBR_p440113	PF00496	dipeptide/oligopeptide		
G8AR24	AZOBR_p130073	PF03480	C4-dicarboxylate		
G8AUM2	AZOBR_p220094	PF00497	amino acid (glutamine/glutamate/aspartate?)		
G8AZZ0	AZOBR_p460010	PF03401	tricarboxylate		
G8AU31	AZOBR_p210104	PF00497	amino acid (glutamine/glutamate/aspartate?)		
G8AUL0	AZOBR_p220082	PF04069	glycine betaine		
G8ALT4	AZOBR_120040	PF00497	amino acid (glutamine/glutamate/aspartate?)		
G8AJ39	AZOBR_40420	PF00497	amino acid (glutamine/glutamate/aspartate?)		

G8AT82	AZOBR_p1110107	PF01497	iron-hydroxamate		
G8AT72	AZOBR_p1110097	PF01497	iron-hydroxamate		
G8AX23	AZOBR_p310174	PF13458	urea		
G8APA0	AZOBR_p1140088	PF09084	nitrate/sulfonate/taurine		
G8ARG2	AZOBR_p130211	PF16868	phosphonate		
G8AIJ7	AZOBR_40228	PF00496	dipeptide/oligopeptide		
G8ASK6	AZOBR_p180012	PF01547	sugar		
G8ASI9	AZOBR_p170156	PF00497	amino acid (glutamine/glutamate/aspartate?)		
G8AJJ8	AZOBR_70046	PF12849	2-aminoethylphosphonate		
G8AIL9	AZOBR_40250	PF16868	glycine betaine		
G8AVT9	AZOBR_p270244	PF09084	glycine betaine		
G8AW38	AZOBR_p280033	PF13458	leucine/valine		
G8AVT4	AZOBR_p270239	PF09084	nitrate/sulfonate/taurine		
G8AR79	AZOBR_p130128	PF03480	C4-dicarboxylate		
G8APQ4	AZOBR_p1170023	PF00496	dipeptide/oligopeptide		
G8ALJ3	AZOBR_110079	PF13458	leucine/valine		
G8AW23	AZOBR_p280018	PF01547	sugar		
G8AXQ0	AZOBR_p330060	PF09084	phosphonates		
***Pectobacterium atrosepticum* SCRI1043**					
Number of ORFs in the proteome: 4459					
Number of SBPs: 85 (1.91% of total)					
Lifestyle Summary: PLANT PATHOGEN					
**SeqID**	**Gene Locus**	**Pfam Code**	**Ligand prediction by TransportDB**	**Experimentally identified ligands**	**References/pdb accession codes**
Q6CZ31	ECA4322	PF13416	sugar		
Q6D1Z3	ECA3304	PF01497	iron-hydroxamate		
Q6D3B0	ECA2834	PF00496	dipeptide/oligopeptide		
Q6CZA4	ECA4249	PF13416	spermidine/putrescine		
Q6D1F3	ECA3495	PF00497	amino acid (glutamine/glutamate/aspartate?)		
Q6D5A6	ECA2134	PF13416	2-aminoethylphosphonate		

Q6DA98	ECA0357	PF03480	C4-dicarboxylate		
Q6D430	ECA2564	PF01497	iron-hydroxamate		
Q6D1C2	ECA3526	PF03180	methionine		
Q6CYW1	ECA4394	PF00496	dipeptide/oligopeptide		
Q6D4A9	ECA2484	PF01297	zinc ion		
Q6D5R3	ECA1977	PF00496	oligopeptide		
Q6D3K8	ECA2736	PF00496	oligopeptide		
Q6D3Q5	ECA2689	PF03180	methionine		
Q6D4G1	ECA2429	PF13416	sugar		
Q6D6Y0	ECA1549	PF00496	dipeptide/oligopeptide		
Q6D2X8	ECA2966	PF00497	amino acid (glutamine/glutamate/aspartate?)		
Q6D250	ECA3246	PF01497	iron-hydroxamate		
Q6D1Y6	ECA3311	PF01497	iron-hydroxamate		
Q6CZ98	ECA4255	PF01497	cobalamin		
Q6D3D5	ECA2809	PF01497	iron-hydroxamate		
Q6D1B1	ECA3537	PF00497	amino acid (glutamine/glutamate/aspartate?)		
Q6D701	ECA1528	PF04069	glycine betaine		
Q6D583	ECA2157	PF00497	amino acid (glutamine/glutamate/aspartate?)		
Q6CZS5	ECA4076	PF00496	dipeptide/oligopeptide		
Q6D8X3	ECA0848	PF13416	sugar		
Q6DB26	ECA0075	PF00496	dipeptide/oligopeptide		
Q6D898	ECA1076	PF01497	iron-hydroxamate		
Q6D6X1	ECA1558	PF09084	phosphonates		
Q6D532	ECA2210	PF13416	sugar		
Q6D7L1	ECA1314	PF00497	amino acid (glutamine/glutamate/aspartate?)		
Q6D8W5	ECA0856	PF00497	amino acid (glutamine/glutamate/aspartate?)		
Q6D4W7	ECA2273	PF00532	ribose		

Q6D3H6	ECA2768	PF00497	amino acid (glutamine/glutamate/aspartate?)		
Q6D584	ECA2156	PF00497	amino acid (glutamine/glutamate/aspartate?)		
Q6D8G6	ECA1008	PF00496	dipeptide/oligopeptide		
Q6DAK8	ECA0245	PF00497	amino acid (glutamine/glutamate/aspartate?)		
Q6D2J9	ECA3096	PF01497	iron-hydroxamate		
Q6D0P7	ECA3751	PF13416	sugar		
Q6D3S6	ECA2668	PF00497	amino acid (glutamine/glutamate/aspartate?)		
Q6D5U6	ECA1942	PF00496	oligopeptide		
Q6CZ52	ECA4301	PF09084	nitrate/sulfonate/taurine		
Q6CZB7	ECA4236	PF13407	xylose		
Q6D643	ECA1845	PF01497	iron-hydroxamate		
Q6CZF7	ECA4195	PF00497	amino acid (glutamine/glutamate/aspartate?)		
Q6D728	ECA1498	PF03180	methionine		
Q6D3E3	ECA2801	PF00496	dipeptide/oligopeptide		
Q6D8Z3	ECA0828	PF00497	amino acid (glutamine/glutamate/aspartate?)		
Q6D3S3	ECA2671	PF00497	amino acid (glutamine/glutamate/aspartate?)		
Q6DAW5	ECA0137	PF00496	dipeptide/oligopeptide		
Q6D4D9	ECA2453	PF13416	spermidine/putrescine		
Q6D967	ECA0752	PF13416	sugar		
Q6D5H4	ECA2066	PF03180	methionine		
Q6CZP1	ECA4110	PF01497	iron-hydroxamate		
Q6D184	ECA3564	PF01497	iron-hydroxamate		
Q6D5X6	ECA1912	PF09084	nitrate/sulfonate/taurine		
Q6D4R6	ECA2324	PF00496	oligopeptide		
Q6CYL8	ECA4489	PF00497	amino acid (glutamine/glutamate/aspartate?)		

Q6DB85	ECA0013	PF13407	ribose		
Q6D4I4	ECA2406	PF01547	sugar		
Q6D2P5	ECA3050	PF00497	amino acid (glutamine/glutamate/aspartate?)		
Q6D4R5	ECA2325	PF00496	oligopeptide		
Q6CZR6	ECA4085	PF00497	amino acid (glutamine/glutamate/aspartate?)		
Q6D863	ECA1112	PF12849	phosphate		
Q6DB04	ECA0098	PF13407	xylose		
Q6D766	ECA1459	PF13407	xylose		
Q6CYN7	ECA4470	PF00497	amino acid (glutamine/glutamate/aspartate?)		
Q6D197	ECA3551	PF13416	sugar		
Q6D2Y1	ECA2963	PF00496	dipeptide/oligopeptide		
Q6DAD2	ECA0322	PF04348	leucine/valine		
Q6CYU5	ECA4410	PF09084	nitrate/sulfonate/taurine		
Q6D7C0	ECA1405	PF13407	ribose		
Q6D5P9	ECA1992	PF00496	oligopeptide		
Q6D5I5	ECA2055	PF13416	spermidine/putrescine		
Q6DA15	ECA0448	PF00496	dipeptide/oligopeptide		
Q6D0P0	ECA3758	PF01297	manganese/zinc ion		
Q6D4J8	ECA2392	PF01297	manganese/zinc ion		
Q6D3R7	ECA2677	PF13416	spermidine/putrescine		
Q6D2C1	ECA3175	PF13416	sugar (maltose?)		
Q6D255	ECA3241	PF01497	iron-hydroxamate		
Q6CYS9	ECA4426	PF00496	dipeptide/oligopeptide		
Q6DAK6	ECA0247	PF00497	amino acid (glutamine/glutamate/aspartate?)		
Q6CZ12	ECA4341	PF13458	leucine/valine		
Q6D1E4	ECA3504	PF04069	glycine betaine		
Q6CYN0	ECA4477	PF12849	phosphate		
***Bdellovibrio bacteriovorus* HD100**					

Number of ORFs in the proteome: 3583					
Number of SBPs: 42 (1.17% of total)					
Lifestyle Summary: SOIL					
**SeqID**	**Gene Locus**	**Pfam Code**	**Ligand prediction by TransportDB**	**Experimentally identified ligands**	**References/pdb accession codes**
Q6MM71	Bd1771	PF00497	amino acid (glutamine/glutamate/aspartate?)		
Q6MHH1	Bd3573	PF00497	amino acid (glutamine/glutamate/aspartate?)		
Q6MHZ9	Bd3385	PF13458	leucine/valine		
Q6MN45	Bd1418	PF13416	spermidine/putrescine		
Q6MJC6	Bd2855	PF00497	amino acid (glutamine/glutamate/aspartate?)		
Q6MJQ5	Bd2714	PF13407	rhamnose		
Q6MQM0	Bd0445	PF02608	sugar		
Q6MP13	Bd1057	PF00497	amino acid (glutamine/glutamate/aspartate?)		
Q6MJA4	Bd2877	PF00497	amino acid (glutamine/glutamate/aspartate?)		
Q6MPM3	Bd0825	PF00497	amino acid (glutamine/glutamate/aspartate?)		
Q6MQU9	Bd0357	PF00496	dipeptide/oligopeptide		
Q6MQ83	Bd0597	PF00497	amino acid (glutamine/glutamate/aspartate?)		
Q6MKE6	Bd2450	PF00497	amino acid (glutamine/glutamate/aspartate?)		
Q6ML31	Bd2191	PF00496	dipeptide/oligopeptide		
Q6MN34	Bd1429	PF00497	amino acid (glutamine/glutamate/aspartate?)		
Q6MLN5	Bd1972	PF00496	oligopeptide		
Q6MNL1	Bd1237	PF00497	amino acid (glutamine/glutamate/aspartate?)		
Q6MLI9	Bd2020	PF09084	nitrate/sulfonate/taurine		

Q6MHI7	Bd3555	PF00497	amino acid (glutamine/glutamate/aspartate?)		
Q6MHV2	Bd3433	PF00497	Not annotated in TransportDB		
Q6MN84	Bd1376	PF13407	ribose		
Q6MR42	Bd0257	PF00497	amino acid (glutamine/glutamate/aspartate?)		
Q6MJJ4	Bd2783	PF00497	amino acid (glutamine/glutamate/aspartate?)		
Q6MJW5	Bd2651	PF00497	amino acid (glutamine/glutamate/aspartate?)		
Q6MPZ9	Bd0689	PF00497	amino acid (glutamine/glutamate/aspartate?)		
Q6MM58	Bd1786	PF00497	amino acid (glutamine/glutamate/aspartate?)		
Q6MQH8	Bd0489	PF00497	amino acid (glutamine/glutamate/aspartate?)		
Q6MI09	Bd3371	PF01297	manganese/zinc ion		
Q6MLC3	Bd2089	PF00497	amino acid (glutamine/glutamate/aspartate?)		
Q6MGQ1	Bd3873	PF13458	leucine/valine		
Q6MPR8	Bd0777	PF00497	amino acid (glutamine/glutamate/aspartate?)		
Q6MM70	Bd1773	PF00497	amino acid (glutamine/glutamate/aspartate?)		
Q6MLH0	Bd2041	PF00497	amino acid (glutamine/glutamate/aspartate?)		
Q6MMH3	Bd1658	PF12727	phosphate		
Q6MNB1	Bd1349	PF00497	amino acid (glutamine/glutamate/aspartate?)		
Q6MPK8	Bd0844	PF00497	Unclassified		
Q6MLM0	Bd1987	PF00497	amino acid (glutamine/glutamate/aspartate?)		

Q6MQG3	Bd0505	PF00497	amino acid (glutamine/glutamate/aspartate?)		
Q6MKK3	Bd2385	PF01497	iron-hydroxamate		
Q6MPW1	Bd0728	PF00497	amino acid (glutamine/glutamate/aspartate?)		
Q6MQ19	Bd0668	PF00497	amino acid (glutamine/glutamate/aspartate?)		
Q6MHU5	Bd3441	PF00497	Unclassified		
***Thermotoga maritima* MSB8**					
Number of ORFs in the proteome: 1852					
Number of SBPs: 34 (1.84% of total)					
Lifestyle Summary: SEDIMENTS (MARINE/FRESH WATER)					
**SeqID**	**Gene Locus**	**Pfam Code**	**Ligand prediction by TransportDB**	**Experimentally identified ligands**	**References/pdb accession codes**
Q9WXX7	TM_0123	PF01297	zinc		
Q9WYF8	TM_0322	PF03480	C4-dicarboxylate		
Q9WXR2	TM_0056	PF00496	dipeptide/oligopeptide	Oligopeptides	doi: 10.1007/s00792-016-0861-7
Q9X0K4	TM_1120	PF13416	sugar		
Q9S5Y1	TM_1839	PF13416	sugar (maltose?)	Maltotetraose, maltotriose	doi: 10.1021/acs.biochem.8b00783; PDB: 2FNC
Q9X053	TM_0958	PF13407	ribose	Ribose	doi: 10.1186/1472-6807-8-50
Q9WZR7	TM_0810	PF01547	sugar		
Q9X0S6	TM_1199	PF00496	dipeptide/oligopeptide		
Q9WZ01	TM_0531	PF00496	dipeptide/oligopeptide		
Q9X0N4	TM_1150	PF00496	dipeptide/oligopeptide		
Q9WXS6	TM_0071	PF00496	dipeptide/oligopeptide		

Q9X0V0	TM_1223	PF00496	dipeptide/oligopeptide	Mannobiose, mannohexaose, xylobiose, xylopentaose, laminaribiose, cellobiose, mannopentaose, cellopentaose	doi: 10.1074/jbc.M114.590992
Q9WXV7	TM_0102	PF02608	sugar		
Q9WYP9	TM_0418	PF01547	sugar (maltose?)		
Q9WYV4	TM_0484	PF09084	nitrate/sulfonate/taurine		
Q9WYE5	TM_0309	PF00496	dipeptide/oligopeptide		
Q9WZ62	TM_0593	PF00497	amino acid (glutamine/glutamate/aspartate?)	Arginine	doi: 10.1371/journal.pone.0096560; doi: 10.1016/j.bbapap.2018.05.016; doi: 10.1016/j.ijbiomac.2018.07.172
Q9X0T1	TM_1204	PF13416	sugar (maltose?)	Maltotetraose, maltotriose	doi: 10.1021/acs.biochem.8b00783; PDB: 2GHA
Q9WXT5	TM_0080	PF01497	iron-hydroxamate		
Q9X195	TM_1375	PF13416	spermidine/putrescine		
Q9WZ64	TM_0595	PF13416	sugar		
Q9X2H0	TM_1855	PF01547	sugar		
Q9X268	TM_1746	PF00496	oligopeptide		
Q9WXN8	TM_0031	PF00496	dipeptide/oligopeptide	Cellobiose, laminaribiose, cellotriose, laminaripentaose cellopentaose, cellohexaose	doi: 10.1074/jbc.M109.041624; doi: 10.1128/AEM.72.2.1336-1345.2006; doi: 10.1074/jbc.M109.041624; PDB: 4JSO

Q9X0V3	TM_1226	PF00496	dipeptide/oligopeptide	Mannobiose, mannohexaose, xylobiose, xylopentaose, laminaribiose, cellobiose, mannopentaose, cellopentaose	doi: 10.1074/jbc.M114.590992
Q9WXW9	TM_0114	PF13407	rhamnose	Glucose, mannose, xylose and galactose	doi: 10.1110/ps.072969407; doi: 10.1107/S1744309112045241; PDB: 3C6Q
Q9WY32	TM_0189	PF01497	iron-hydroxamate	Nickel	PDB: 2ETV
Q9WYT2	TM_0460	PF00496	dipeptide/oligopeptide		
Q9X0Z1	TM_1264	PF12849	phosphate		
Q9WYR3	TM_0432	PF01547	sugar		
Q9X0W2	TM_1235	PF13416	sugar		
Q9X0L9	TM_1135	PF13458	leucine/valine	Phenylalanine	PDB: 3TD9
Q9X0F7	TM_1067	PF00496	Unclassified		
Q9WYD6	TM_0300	PF00496	dipeptide/oligopeptide		
***Shewanella oneidensis* MR-1**					
Number of ORFs in the proteome: 4068					
Number of SBPs: 28 (0.69% of total)					
Lifestyle Summary: SEDIMENTS (MARINE/FRESH WATER)					
**SeqID**	**Gene Locus**	**Pfam Code**	**Ligand prediction by TransportDB**	**Experimentally identified ligands**	**References/pdb accession codes**
Q8EA34	SO_4075	PF00497	Unclassified		
Q8EBT7	SO_3412	PF00497	amino acid (glutamine/glutamate/aspartate?)		
Q8EHG1	SO_1260	PF00497	amino acid (glutamine/glutamate/aspartate?)		
Q8EI09	SO_1044	PF00497	amino acid (glutamine/glutamate/aspartate?)		

Q8EGT8	SO_1503	PF00497	amino acid (glutamine/glutamate/aspartate?)		
Q8EJA3	SO_0565	PF01297	manganese/zinc ion		
Q8EK06	SO_0300	PF04348	Not annotated in TransportDB		
Q8E9I5	SO_4292	PF12849	phosphate		
Q8EBM9	SO_3481	PF00497	amino acid (glutamine/glutamate/aspartate?)		
Q8EHW2	SO_1100	PF03480	C4-dicarboxylate		
Q8EC12	SO_3333	PF01547	sugar		
Q8EHJ2	SO_1229	PF00532	trimethylamine N-oxide		
Q8ECK4	SO_3134	PF03480	C4-dicarboxylate	Succinate	doi: 10.1021/bi501388y
Q8EGN9	SO_1560	PF12849	phosphate		
Q8EG09	SO_1805	PF00496	dipeptide/oligopeptide		
Q8EFX6	SO_1839	PF00497	amino acid (glutamine/glutamate/aspartate?)		
Q8EJK7	SO_0456	PF16868	phosphonates		
Q8EKN6	SO_0056	PF00497	amino acid (glutamine/glutamate/aspartate?)		
Q8EHS4	SO_1143	PF00497	amino acid (glutamine/glutamate/aspartate?)		
Q8EJH9	SO_0486	PF05048	Not annotated in TransportDB		
Q8EAN8	SO_3860	PF00497	amino acid (glutamine/glutamate/aspartate?)		
Q8EHF3	SO_1270	PF13416	spermidine/putrescine		
Q8EB61	SO_3673	PF01497	iron-hydroxamate		
Q8E8F1	SO_4714	PF00497	amino acid (glutamine/glutamate/aspartate?)		
Q8EFL2	SO_1959	PF04069	glycine betaine		
Q8E8E6	SO_4719	PF12849	tungsten		
Q8EB26	SO_3709	PF01497	iron-hydroxamate		
Q8EB22	SO_3714	PF13407	rhamnose		
***Staphylococcus aureus* NCTC 8325**					

Number of ORFs in the proteome: 2889					
Number of SBPs: 21 (0.73% of total)					
Lifestyle Summary: HUMAN/ANIMAL PATHOGEN					
**SeqID**	**Gene Locus**	**Pfam Code**	**Ligand prediction by TransportDB**	**Experimentally identified ligands**	**References/pdb accession codes**
Q2FYP6	SAOUHSC_01389	PF12849	phosphate		
Q2G2P5	SAOUHSC_00201	PF00496	dipeptide/oligopeptide	Nickel, histidine, Ni-(l-His)2 complex, Ni-(l-His) (2-methyl-thiazolidine dicarboxylate) complex	doi: 10.1111/j.1365-2958.2010.07287.x; doi: 10.1039/c4mt00295d; PDB: 3RQT, 4OFJ, 4XKN
Q2FVE7	SAOUHSC_02767	PF00496	nickel	Metallophore staphylopine with nickel, cobalt and zinc	doi: 10.1111/mmi.12126; doi: 10.1073/pnas.1718382115
Q2FZE6	SAOUHSC_01085	PF01497	heme	Ferric and ferrous heme	doi: 10.1016/j.bbrc.2004.06.025; doi: 10.1021/bi7009585
Q2G2D8	SAOUHSC_00634	PF01297	zinc		
Q2FZR2	SAOUHSC_00928	PF00496	dipeptide/oligopeptide		
Q2FZR3	SAOUHSC_00927	PF00496	oligopeptide		
Q2G2A8	SAOUHSC_01049	PF13416	spermidine/putrescine		
Q2FW75	SAOUHSC_02430	PF01497	iron-hydroxamate		
Q2FWN6	SAOUHSC_02246	PF01497	iron-hydroxamate		
Q2G071	SAOUHSC_00749	PF01497	iron-hydroxamate		
Q2G1E9	SAOUHSC_00176	PF13416	sugar (maltose?)	Maltose	PDB: 4HW8
Q2FZZ0	SAOUHSC_00844	PF03180	methionine		
Q2G0V0	SAOUHSC_00426	PF03180	methionine		
Q2FVH0	SAOUHSC_02742	PF04069	glycine betaine		
Q2G1N4	SAOUHSC_00074	PF01497	iron-hydroxamate		
Q2FVL2	SAOUHSC_02699	PF00497	amino acid (glutamine/glutamate/aspartate?)		
Q2FZM4	SAOUHSC_00976	PF01497	iron-hydroxamate		

Q2FVW9	SAOUHSC_02554	PF01497	iron-hydroxamate	Hydroxamate siderophores (e.g. ferrichrome, ferrioxamine-B)	doi: 10.1042/BJ20121426; doi: 10.1021/bi401349d.
Q2G0F6	SAOUHSC_00613	PF01497	iron-hydroxamate		
Q2G1F5	SAOUHSC_00170	PF00496	dipeptide/oligopeptide		
***Yersinia pestis* Nepal516**					
Number of ORFs in the proteome: 3946					
Number of SBPs: 69 (1.75% of total)					
Lifestyle Summary: HUMAN/ANIMAL PATHOGEN					
**SeqID**	**Gene Locus**	**Pfam Code**	**Ligand prediction by TransportDB**	**Experimentally identified ligands**	**References/pdb accession codes**
Q1CNC9	YPN_0168	PF13416	sugar		
Q1CN18	YPN_0279	PF13407	rhamnose		
Q1CLU2	YPN_0706	PF01497	iron-hydroxamate		
A0A0H2YMN	YPN_MT0044	PF09084	nitrate/sulfonate/taurine		
A0A0H2YI45	YPN_1799	PF00496	oligopeptide		
A0A0H2YLS4	YPN_3431	PF04348	Not annotated in TransportDB		
A0A0H2YIR0	YPN_2461	PF13407	ribose		
A0A0H2YIL8	YPN_1745	PF13407	xylose		
A0A0H2YKX	YPN_2703	PF00496	Unclassified		
A0A0H2YK5	YPN_3062	PF13407	xylose		
A0A0H2YH5	YPN_1131	PF04069	glycine betaine		
A0A0H2YJ85	YPN_2685	PF13407	xylose		
A0A0H2YJY6	YPN_2277	PF00497	amino acid (glutamine/glutamate/aspartate?)		
A0A0H2YE5	YPN_0558	PF01497	iron-hydroxamate		
A0A0H2YGB	YPN_1506	PF13416	spermidine/putrescine		
A0A0H2YJV3	YPN_2107	PF00497	amino acid (glutamine/glutamate/aspartate?)		
A0A0H2YJD3	YPN_2311	PF13407	rhamnose		
A0A0H2YET	YPN_0475	PF01547	sugar		

A0A0H2YES8	YPN_0027	PF01547	sugar (maltose?)		
A0A0H2YGV	YPN_0765	PF13407	rhamnose		
A0A0H2YFZ4	YPN_1335	PF13416	iron(III)		
A0A0H2YIS1	YPN_2472	PF13407	xylose		
A0A0H2YK01	YPN_2173	PF00497	amino acid (glutamine/glutamate/aspartate?)		
A0A0H2YKE6	YPN_2444	PF01497	iron-hydroxamate		
A0A0H2YJ47	YPN_2632	PF00497	amino acid (glutamine/glutamate/aspartate?)		
A0A0H2YFR5	YPN_0839	PF00497	amino acid (glutamine/glutamate/aspartate?)		
A0A0H2YFB4	YPN_1065	PF13407	xylose		
A0A0H2YIJ5	YPN_2401	PF03480	C4-dicarboxylate		
A0A0H2YK9	YPN_2662	PF03180	methionine		
A0A0H2YIS2	YPN_1648	PF00496	oligopeptide		
A0A0H2YIJ6	YPN_1724	PF00532	ribose		
A0A0H2YJ61	YPN_2232	PF01547	sugar		
A0A0H2YIE4	YPN_1893	PF01297	zinc		
A0A0H2YFL2	YPN_0775	PF13407	ribose		
A0A0H2YKN	YPN_2781	PF04069	glycine betaine		
A0A0H2YHF4	YPN_1538	PF01297	zinc		
A0A0H2YKZ8	YPN_2928	PF03180	methionine		
A0A0H2YFY3	YPN_1295	PF13416	spermidine/putrescine		
A0A0H2YNK	YPN_3974	PF12849	phosphate		
A0A0H2YDE4	YPN_0156	PF13458	leucine/valine		
A0A0H2YI63	YPN_1815	PF00496	oligopeptide		
A0A0H2YG1	YPN_0615	PF01547	sugar		
A0A0H2YJ69	YPN_2669	PF01497	iron-hydroxamate		
A0A0H2YFH4	YPN_1100	PF00497	amino acid (glutamine/glutamate/aspartate?)		
A0A0H2YHT	YPN_2071	PF13416	sugar		
A0A0H2YLS0	YPN_3611	PF13407	xylose		
A0A0H2YM4	YPN_3652	PF00496	dipeptide/oligopeptide		

A0A0H2YK8(	YPN_2649	PF13416	spermidine/putrescine		
A0A0H2YMS	YPN_3885	PF04069	glycine betaine		
A0A0H2YEX(	YPN_0886	PF12849	phosphate		
A0A0H2YKF4	YPN_3153	PF01497	iron-hydroxamate		
A0A0H2YNK	YPN_3968	PF00497	amino acid (glutamine/glutamate/aspartate?)		
A0A0H2YLN(	YPN_3537	PF13407	rhamnose		
A0A0H2YM7	YPN_3157	PF13407	xylose		
A0A0H2YEI4	YPN_0640	PF00496	dipeptide/oligopeptide		
A0A0H2YEJ4	YPN_0699	PF01497	iron-hydroxamate		
A0A0H2YF52	YPN_0059	PF13407	xylose		
A0A0H2YN3	YPN_3670	PF01497	iron-hydroxamate		
A0A0H2YHE	YPN_1911	PF01547	sugar		
A0A0H2YKS(	YPN_2641	PF01497	iron-hydroxamate		
A0A0H2YJI5	YPN_2780	PF04069	glycine betaine		
A0A0H2YFI3	YPN_1143	PF00496	dipeptide/oligopeptide		
A0A0H2YK7!	YPN_2643	PF00497	amino acid (glutamine/glutamate/aspartate?)		
A0A0H2YM(	YPN_3386	PF01497	iron-hydroxamate		
A0A0H2YMF	YPN_3546	PF09084	nitrate/sulfonate/taurine		
A0A0H2YJU3	YPN_2095	PF13407	xylose		
A0A0H2YLZ6	YPN_3058	PF13416	sugar (maltose?)		
A0A0H2YGT	YPN_0750	PF00497	amino acid (glutamine/glutamate/aspartate?)		
A0A0H2YM7	YPN_3684	PF13407	xylose		
***Anabaena cylindrica* PCC 7122**					
Number of ORFs in the proteome: 5797					
Number of SBPs: 28 (0.48% of total)					
Lifestyle Summary: FRESH/MARINE WATERS					
**SeqID**	**Gene Locus**	**Pfam Code**	**Ligand prediction by TransportDB**	**Experimentally identified ligands**	**References/pdb accession codes**
K9ZLI1	Anacy_4280	PF12849	phosphate		

K9ZDH1	Anacy_1759	PF09084	nitrate/sulfonate/taurine		
K9ZLK4	Anacy_4052	PF00497	amino acid (glutamine/glutamate/aspartate?)		
K9ZDQ5	Anacy_1853	PF01547	sugar		
K9ZMB3	Anacy_5033	PF00496	dipeptide/oligopeptide		
K9ZDY8	Anacy_1266	PF09084	nitrate/sulfonate/taurine		
K9ZJ32	Anacy_2938	PF01497	iron-hydroxamate		
K9Z908	Anacy_0041	PF01297	zinc		
K9ZJU9	Anacy_3410	PF03480	C4-dicarboxylate		
K9ZC17	Anacy_0328	PF16868	phosphonates		
K9ZF45	Anacy_1688	PF01297	zinc		
K9ZMG6	Anacy_5057	PF00497	amino acid (glutamine/glutamate/aspartate?)		
K9ZEL9	Anacy_1269	PF09084	glycine betaine		
K9ZEC3	Anacy_2062	PF01547	sugar		
K9ZFT0	Anacy_2643	PF00496	dipeptide/oligopeptide		
K9ZIS4	Anacy_2786	PF12849	phosphate		
K9ZLG0	Anacy_4260	PF01497	iron-hydroxamate		
K9ZG83	Anacy_2783	PF12849	phosphate		
K9ZIK1	Anacy_3179	PF13416	spermidine/putrescine		
K9ZAR1	Anacy_0691	PF01497	iron-hydroxamate		
K9ZPV6	Anacy_5046	PF12849	phosphate		
K9ZMQ7	Anacy_5152	PF13416	sugar		
K9ZJ48	Anacy_3873	PF01094	leucine/valine		
K9ZPQ2	Anacy_5449	PF01497	iron-hydroxamate		
K9ZCL6	Anacy_1410	PF01297	zinc		
K9ZIG1	Anacy_3144	PF12727	phosphate		
K9ZLM4	Anacy_4078	PF01497	iron-hydroxamate		
K9ZQJ4	Anacy_5495	PF12849	phosphate		
***Serratia plymuthica* S13**					
Number of ORFs in the proteome: 4986					
Number of SBPs: 95 (1.91% of total)					

Lifestyle Summary: BENEFICIAL PLANT ASSOCIATED					
**SeqID**	**Gene Locus**	**Pfam Code**	**Ligand prediction by TransportDB**	**Experimentally identified ligands**	**References/pdb accession codes**
S4YK96	M621_04840	PF13407	rhamnose		
S4YIL6	M621_12240	PF13407	xylose		
S4YI45	M621_09920	PF13407	xylose		
S4YL11	M621_16065	PF09084	nitrate/sulfonate/taurine		
S4YJ07	M621_13460	PF00496	oligopeptide		
S4YP20	M621_23360	PF01547	sugar (maltose?)		
S4YI76	M621_11645	PF01547	sugar		
S4YPH6	M621_16675	PF09084	nitrate/sulfonate/taurine		
S4YH44	M621_09660	PF00497	amino acid (glutamine/glutamate/aspartate?)		
S4YJ92	M621_12765	PF03180	methionine		
S4YLZ4	M621_08450	PF13416	spermidine/putrescine		
S4YIC6	M621_16325	PF01497	iron-hydroxamate		
S4YH87	M621_09940	PF03180	methionine		
S4YKL6	M621_17205	PF00496	oligopeptide		
S4YPH4	M621_13880	PF00496	oligopeptide		
S4YIK0	M621_10915	PF01497	iron-hydroxamate		
S4YTG5	M621_23240	PF00497	amino acid (glutamine/glutamate/aspartate?)		
S4YIS1	M621_12780	PF09084	glycine betaine		
S4YCT3	M621_00915	PF13458	leucine/valine		
S4YGS8	M621_07025	PF01497	iron-hydroxamate		
S4YMM4	M621_09935	PF13407	xylose		
S4YJ21	M621_12045	PF13407	ribose		
S4YPB3	M621_16125	PF13407	ribose		
S4YGS9	M621_06925	PF03180	methionine		
S4YL86	M621_16880	PF13458	leucine/valine		
S4YFK1	M621_04905	PF12849	phosphate		
S4YEA5	M621_07995	PF13407	ribose		

S4YE25	M621_07610	PF00497	amino acid (glutamine/glutamate/aspartate?)		
S4YIU9	M621_12985	PF13407	ribose		
S4YQ21	M621_25465	PF13407	ribose		
S4YPC3	M621_23840	PF04069	glycine betaine		
S4YJ73	M621_12600	PF13407	xylose		
S4YLT1	M621_07950	PF13407	xylose		
S4YKN4	M621_15030	PF00497	amino acid (glutamine/glutamate/aspartate?)		
S4YCH5	M621_04335	PF00497	amino acid (glutamine/glutamate/aspartate?)		
S4YKW0	M621_17635	PF00497	amino acid (glutamine/glutamate/aspartate?)		
S4YGS6	M621_06995	PF00497	amino acid (glutamine/glutamate/aspartate?)		
S4YMM5	M621_24795	PF13407	ribose		
S4YAF9	M621_00295	PF13407	xylose		
S4YI85	M621_10520	PF09084	nitrate/sulfonate/taurine		
S4YKG7	M621_20890	PF13407	rhamnose		
S4YUX6	M621_25610	PF00497	amino acid (glutamine/glutamate/aspartate?)		
S4YH53	M621_07890	PF00496	dipeptide/oligopeptide		
S4YX86	M621_16705	PF09084	nitrate/sulfonate/taurine		
S4YIX5	M621_13210	PF13458	leucine/valine		
S4YEI3	M621_08495	PF00497	amino acid (glutamine/glutamate/aspartate?)		
S4YLN0	M621_17715	PF03401	tricarboxylate		
S4YFV5	M621_07150	PF03180	methionine		
S4YNA5	M621_11375	PF00532	ribose		
S4YGD2	M621_08480	PF00497	amino acid (glutamine/glutamate/aspartate?)		
S4YEM1	M621_02185	PF00496	dipeptide/oligopeptide		
S4YK48	M621_14100	PF00496	dipeptide/oligopeptide		

S4YL84	M621_22610	PF04348	Not annotated in TransportDB		
S4YIY4	M621_11910	PF00496	dipeptide/oligopeptide		
S4YJK9	M621_13545	PF00496	oligopeptide		
S4YQ98	M621_03605	PF00497	amino acid (glutamine/glutamate/aspartate?)		
S4YDH4	M621_00475	PF00496	dipeptide/oligopeptide		
S4YDM9	M621_00945	PF13416	sugar		
S4YP37	M621_12745	PF01497	iron-hydroxamate		
S4YMZ3	M621_10725	PF01297	zinc		
S4YPN3	M621_19345	PF13416	spermidine/putrescine		
S4YJS9	M621_14910	PF00496	dipeptide/oligopeptide		
S4YEV3	M621_03420	PF01497	iron-hydroxamate		
S4YPA7	M621_16070	PF00497	amino acid (glutamine/glutamate/aspartate?)		
S4YJK0	M621_13095	PF00496	oligopeptide		
S4YMN3	M621_19910	PF04069	glycine betaine		
S4YMU9	M621_10445	PF09084	nitrate/sulfonate/taurine		
S4YS73	M621_21115	PF01497	iron-hydroxamate		
S4YG85	M621_11995	PF00497	amino acid (glutamine/glutamate/aspartate?)		
S4YPN9	M621_14265	PF01297	zinc		
S4YMI2	M621_20025	PF03180	methionine		
S4YFG5	M621_04765	PF01497	iron-hydroxamate		
S4YIK1	M621_12130	PF13416	sugar (maltose?)		
S4YCL5	M621_04550	PF00496	dipeptide/oligopeptide		
S4YH59	M621_13875	PF00496	oligopeptide		
S4YIL3	M621_12215	PF01497	iron-hydroxamate		
S4YUE4	M621_24465	PF13407	rhamnose		
S4YGD8	M621_06080	PF00497	amino acid (glutamine/glutamate/aspartate?)		
S4YHN5	M621_08900	PF09084	nitrate/sulfonate/taurine		
S4YJJ0	M621_13015	PF01547	sugar		
S4YPK6	M621_25585	PF12849	phosphate		

S4YL73	M621_18225	PF00496	dipeptide/oligopeptide		
S4YP18	M621_12950	PF03180	methionine		
S4YN85	M621_20985	PF13416	iron(III)		
S4YG35	M621_05330	PF01297	zinc		
S4YIK3	M621_16995	PF00497	amino acid (glutamine/glutamate/aspartate?)		
S4YHV0	M621_14930	PF00496	dipeptide/oligopeptide		
S4YIH5	M621_10760	PF01497	iron-hydroxamate		
S4YFY8	M621_07415	PF04069	glycine betaine		
S4YLS2	M621_18180	PF01497	iron-hydroxamate		
S4YPY1	M621_14665	PF00496	dipeptide/oligopeptide		
S4YNT6	M621_12260	PF00497	amino acid (glutamine/glutamate/aspartate?)		
S4YL55	M621_16550	PF00496	dipeptide/oligopeptide		
S4YL90	M621_16935	PF04069	glycine betaine		
S4YNU4	M621_12345	PF00497	amino acid (glutamine/glutamate/aspartate?)		
***Klebsiella pneumoniae* HS11286**					
Number of ORFs in the proteome: 5728					
Number of SBPs: 88 (1.54% of total)					
Lifestyle Summary: HUMAN/ANIMAL PATHOGEN					
**SeqID**	**Gene Locus**	**Pfam Code**	**Ligand prediction by TransportDB**	**Experimentally identified ligands**	**References/pdb accession codes**
A0A0H3H45	KPHS_49660	PF13416	sugar		
A0A0H3GZF	KPHS_31610	PF03180	methionine		
A0A0H3GQV	KPHS_30000	PF00496	dipeptide/oligopeptide		
A0A0H3GM5	KPHS_17700	PF00497	amino acid (glutamine/glutamate/aspartate?)		
A0A0H3GPU	KPHS_14790	PF13407	ribose		
A0A0H3GTC	KPHS_13410	PF00497	amino acid (glutamine/glutamate/aspartate?)		
A0A0H3GII9	KPHS_09070	PF01497	iron-hydroxamate		

A0A0H3GK5	KPHS_10350	PF00497	amino acid (glutamine/glutamate/aspartate?)		
A0A0H3GTJ1	KPHS_46370	PF13407	rhamnose		
A0A0H3GZM	KPHS_49730	PF13458	leucine/valine		
A0A0H3GUC	KPHS_32440	PF13407	xylose		
A0A0H3GKE	KPHS_16720	PF00497	amino acid (glutamine/glutamate/aspartate?)		
A0A0H3GXI7	KPHS_42180	PF09084	glycine betaine		
A0A0H3GM	KPHS_04640	PF13407	ribose		
A0A0H3GKX	KPHS_02580	PF01547	sugar (maltose?)		
A0A0H3GQ8	KPHS_34170	PF00532	ribose		
A0A0H3GQV	KPHS_17730	PF00497	amino acid (glutamine/glutamate/aspartate?)		
A0A0H3GM	KPHS_18800	PF00496	oligopeptide		
A0A0H3GUR	KPHS_30830	PF01497	iron-hydroxamate		
A0A0H3GPS	KPHS_14590	PF13407	ribose		
A0A0H3GQ4	KPHS_27550	PF09084	nitrate/sulfonate/taurine		
A0A0H3GR2	KPHS_30800	PF00497	amino acid (glutamine/glutamate/aspartate?)		
A0A0H3GX5	KPHS_43240	PF01547	sugar		
A0A0H3GZX	KPHS_50580	PF00496	dipeptide/oligopeptide		
A0A0H3GW	KPHS_41040	PF01297	zinc		
A0A0H3GM2	KPHS_03290	PF13407	rhamnose		
A0A0H3GVX	KPHS_35980	PF00496	dipeptide/oligopeptide		
A0A0H3GNV	KPHS_10490	PF04069	glycine betaine		
A0A0H3GGS	KPHS_00100	PF13407	ribose		
A0A0H3H4N	KPHS_51960	PF03180	methionine		
A0A0H3GVG	KPHS_33930	PF01297	zinc		
A0A0H3GP9	KPHS_30220	PF09084	nitrate/sulfonate/taurine		
A0A0H3H0T	KPHS_36510	PF13407	xylose		
A0A0H3GNY	KPHS_28670	PF00497	amino acid (glutamine/glutamate/aspartate?)		
A0A0H3GWS	KPHS_41640	PF01497	iron-hydroxamate		

A0A0H3GZA	KPHS_30910	PF09084	nitrate/sulfonate/taurine		
A0A0H3GTZ	KPHS_15210	PF00497	amino acid (glutamine/glutamate/aspartate?)		
A0A0H3GTP	KPHS_14310	PF13407	ribose		
A0A0H3GL8	KPHS_14450	PF01497	iron-hydroxamate		
A0A0H3GX4	KPHS_40830	PF04069	glycine betaine		
A0A0H3H0S	KPHS_36310	PF04069	glycine betaine		
A0A0H3GGY	KPHS_02970	PF13416	spermidine/putrescine		
A0A0H3GS7	KPHS_22230	PF00496	oligopeptide		
A0A0H3GN7	KPHS_25870	PF03180	methionine		
A0A0H3GV3	KPHS_52320	PF01547	2-aminoethylphosphonate		
A0A0H3GS1	KPHS_09010	PF01497	iron-hydroxamate		
A0A0H3GM	KPHS_22020	PF00496	oligopeptide		
A0A0H3GXB	KPHS_41530	PF01297	zinc		
A0A0H3GLS	KPHS_21120	PF13416	spermidine/putrescine		
A0A0H3GZ3	KPHS_30160	PF09084	nitrate/sulfonate/taurine		
A0A0H3GTW	KPHS_27930	PF00497	amino acid (glutamine/glutamate/aspartate?)		
A0A0H3GXT	KPHS_53000	PF12849	phosphate		
A0A0H3GZU	KPHS_32960	PF13458	leucine/valine		
A0A0H3GKG	KPHS_16970	PF00496	dipeptide/oligopeptide		
A0A0H3GQH	KPHS_28900	PF00496	dipeptide/oligopeptide		
A0A0H3GYQ	KPHS_50890	PF13407	xylose		
A0A0H3GU7	KPHS_29030	PF13416	spermidine/putrescine		
A0A0H3GP5	KPHS_13180	PF13407	xylose		
A0A0H3GSY	KPHS_26740	PF13407	rhamnose		
A0A0H3GR3	KPHS_30900	PF09084	nitrate/sulfonate/taurine		
A0A0H3GW	KPHS_23360	PF03180	methionine		
A0A0H3GUD	KPHS_49770	PF13458	leucine/valine		
A0A0H3GHK	KPHS_05320	PF13416	sugar		
A0A0H3GTM	KPHS_46920	PF04348	Not annotated in TransportDB		
A0A0H3GZH	KPHS_31910	PF00496	oligopeptide		

A0A0H3GWI	KPHS_37680	PF00497	amino acid (glutamine/glutamate/aspartate?)		
A0A0H3GV0	KPHS_34390	PF00497	amino acid (glutamine/glutamate/aspartate?)		
A0A0H3GJS1	KPHS_14020	PF00497	amino acid (glutamine/glutamate/aspartate?)		
A0A0H3GR7	KPHS_37670	PF00497	amino acid (glutamine/glutamate/aspartate?)		
A0A0H3GKJ8	KPHS_11700	PF13416	sugar (maltose?)		
A0A0H3GQ1	KPHS_27150	PF00497	amino acid (glutamine/glutamate/aspartate?)		
A0A0H3GHC	KPHS_05970	PF13407	rhamnose		
A0A0H3H24	KPHS_42210	PF03180	methionine		
A0A0H3GNN	KPHS_09440	PF03180	methionine		
A0A0H3GU5	KPHS_31390	PF01497	cobalamin		
A0A0H3GSG	KPHS_10410	PF13458	leucine/valine		
A0A0H3GPF	KPHS_14130	PF00496	dipeptide/oligopeptide		
A0A0H3H0V	KPHS_36760	PF00496	oligopeptide		
A0A0H3GL4	KPHS_18520	PF09084	nitrate/sulfonate/taurine		
A0A0H3GPD	KPHS_30670	PF13416	iron(III)		
A0A0H3GPG	KPHS_31070	PF01497	iron-hydroxamate		
A0A0H3GGA	KPHS_00620	PF13407	rhamnose		
A0A0H3GVL	KPHS_20060	PF13416	2-aminoethylphosphonate		
A0A0H3GH8	KPHS_04070	PF00497	amino acid (glutamine/glutamate/aspartate?)		
A0A0H3GX0	KPHS_50050	PF00496	nickel		
A0A0H3GPG	KPHS_13340	PF01497	iron-hydroxamate		
A0A0H3GKV	KPHS_17620	PF13416	spermidine/putrescine		
A0A0H3GTC	KPHS_28240	PF00496	dipeptide/oligopeptide		
***Sphingomonas wittichii* RW1**					
Number of ORFs in the proteome: 5313					
Number of SBPs: 10 (0.19% of total)					

Lifestyle Summary: FRESH/MARINE WATERS					
**SeqID**	**Gene Locus**	**Pfam Code**	**Ligand prediction by TransportDB**	**Experimentally identified ligands**	**References/pdb accession codes**
A5V9L7	Swit_2625	PF00497	amino acid (glutamine/glutamate/aspartate?)		
A5V5A4	Swit_1104	PF12849	phosphate		
A5V7K2	Swit_1908	PF13458	urea		
A5VD32	Swit_3853	PF00496	oligopeptide		
A5V443	Swit_0692	PF00497	amino acid (glutamine/glutamate/aspartate?)		
A5V9A1	Swit_2508	PF09084	nitrate/sulfonate/taurine		
A5V2W4	Swit_0259	PF01497	iron-hydroxamate		
A5V9H3	Swit_2581	PF13458	leucine/valine		
A5VD31	Swit_3852	PF00496	oligopeptide		
A5V3S2	Swit_0570	PF13458	leucine/valine		
***Burkholderia cepacia* 383**					
Number of ORFs in the proteome: 7715					
Number of SBPs: 126 (1.63% of total)					
Lifestyle Summary: SOIL					
**SeqID**	**Gene Locus**	**Pfam Code**	**Ligand prediction by TransportDB**	**Experimentally identified ligands**	**References/pdb accession codes**
Q391B0	Bcep18194_B2845	PF04069	glycine betaine/L-proline		
Q39FX2	Bcep18194_A5050	PF13416	spermidine/putrescine		
Q39G21	Bcep18194_A5001	PF00496	oligopeptide		
Q39P48	Bcep18194_C6719	PF13416	spermidine/putrescine		
Q396G9	Bcep18194_B1528	PF09084	nitrate/sulfonate/taurine		
Q39DE6	Bcep18194_A5926	PF01547	sugar		
Q39DE1	Bcep18194_A5931	PF01297	manganese/zinc ion		
Q396Y7	Bcep18194_B1360	PF13416	spermidine/putrescine		
Q397G3	Bcep18194_B1284	PF00497	amino acid (glutamine/glutamate/aspartate?)		
Q39GH6	Bcep18194_A4845	PF13416	spermidine/putrescine		

Q39I22	Bcep18194_A4297	PF00497	amino acid (glutamine/glutamate/aspartate?)		
Q39LW9	Bcep18194_C7503	PF09084	nitrate/sulfonate/taurine		
Q39HA2	Bcep18194_A4568	PF13407	ribose		
Q39B26	Bcep18194_B0219	PF01497	hemin		
Q395F8	Bcep18194_B1789	PF00532	ribose		
Q39GZ0	Bcep18194_A4681	PF13407	ribose		
Q39BH1	Bcep18194_B0073	PF00497	amino acid (glutamine/glutamate/aspartate?)		
Q391H6	Bcep18194_B2779	PF13416	spermidine/putrescine		
Q39C30	Bcep18194_A6392	PF00496	dipeptide/oligopeptide		
Q397U5	Bcep18194_B1152	PF09084	nitrate/sulfonate/taurine		
Q39JR2	Bcep18194_A3703	PF00532	ribose		
Q39N36	Bcep18194_C7086	PF09084	nitrate/sulfonate/taurine		
Q39GQ9	Bcep18194_A4762	PF00497	amino acid (glutamine/glutamate/aspartate?)		
Q39MK5	Bcep18194_C7267	PF00497	amino acid (glutamine/glutamate/aspartate?)		
Q39HM8	Bcep18194_A4442	PF12849	phosphate		
Q39LB9	Bcep18194_C7703	PF04069	glycine betaine/L-proline		
Q394E2	Bcep18194_B2061	PF09084	nitrate/sulfonate/taurine		
Q397J4	Bcep18194_B1253	PF00496	dipeptide/oligopeptide		
Q39LD9	Bcep18194_C7683	PF13416	spermidine/putrescine		
Q39LU7	Bcep18194_C7525	PF13416	spermidine/putrescine		
Q39GR3	Bcep18194_A4758	PF13416	spermidine/putrescine		
Q39F01	Bcep18194_A5371	PF00496	oligopeptide		
Q39AZ0	Bcep18194_B0255	PF00497	amino acid (glutamine/glutamate/aspartate?)		
Q39LM5	Bcep18194_C7597	PF13458	branched-chain amino acid		
Q39CF6	Bcep18194_A6266	PF00496	dipeptide/oligopeptide		
Q391S7	Bcep18194_B2678	PF13458	branched-chain amino acid		
Q39LH0	Bcep18194_C7652	PF09084	nitrate/sulfonate/taurine		
Q39J37	Bcep18194_A3930	PF16868	C4-dicarboxylate		

Q398W3	Bcep18194_B0884	PF13407	ribose		
Q396G7	Bcep18194_B1530	PF00497	amino acid (glutamine/glutamate/aspartate?)		
Q39DU1	Bcep18194_A5781	PF01497	cobalamin/Fe3+-siderophores		
Q392C3	Bcep18194_B2582	PF03180	D-methionine		
Q39HK1	Bcep18194_A4469	PF13458	branched-chain amino acid		
Q39I42	Bcep18194_A4277	PF13458	branched-chain amino acid		
Q39L46	Bcep18194_A3218	PF09084	nitrate/sulfonate/taurine		
Q394D6	Bcep18194_B2067	PF00496	dipeptide/oligopeptide		
Q39BC9	Bcep18194_B0115	PF00496	oligopeptide		
Q39P84	Bcep18194_C6682	PF00497	amino acid (glutamine/glutamate/aspartate?)		
Q397D8	Bcep18194_B1309	PF03480	C4-dicarboxylate		
Q394D4	Bcep18194_B2069	PF09084	nitrate/sulfonate/taurine		
Q39A98	Bcep18194_B0499	PF04069	glycine betaine/L-proline/carnitine/choline		
Q39LC8	Bcep18194_C7694	PF04069	glycine betaine/L-proline		
Q39LS1	Bcep18194_C7551	PF04069	glycine betaine/L-proline/carnitine/choline		
Q398C7	Bcep18194_B1070	PF12849	phosphate		
Q39DZ1	Bcep18194_A5731	PF00497	amino acid (glutamine/glutamate/aspartate?)		
Q39KC2	Bcep18194_A3492	PF13416	sugar		
Q39N17	Bcep18194_C7105	PF01497	cobalamin/Fe3+-siderophores		
Q39M36	Bcep18194_C7436	PF03480	C4-dicarboxylate		
Q392N6	Bcep18194_B2469	PF00497	amino acid (glutamine/glutamate/aspartate?)		
Q39KN3	Bcep18194_A3381	PF13458	branched-chain amino acid		
Q39A17	Bcep18194_B0580	PF04069	glycine betaine/L-proline		
Q392H0	Bcep18194_B2535	PF13458	branched-chain amino acid		
Q39BT1	Bcep18194_A6491	PF00497	amino acid (glutamine/glutamate/aspartate?)		
Q390R0	Bcep18194_B2945	PF13458	branched-chain amino acid		

Q39CR1	Bcep18194_A6161	PF04392	Unclassified		
Q392Y4	Bcep18194_B2371	PF13407	ribose		
Q399X1	Bcep18194_B0626	PF13407	ribose		
Q394Q3	Bcep18194_B1950	PF13458	branched-chain amino acid		
Q399Y6	Bcep18194_B0611	PF04069	glycine betaine/L-proline		
Q39LD0	Bcep18194_C7692	PF13458	branched-chain amino acid		
Q39KZ3	Bcep18194_A3271	PF13458	branched-chain amino acid		
Q39AT6	Bcep18194_B0309	PF03180	D-methionine		
Q39KW9	Bcep18194_A3295	PF13458	branched-chain amino acid		
Q39KX3	Bcep18194_A3291	PF00497	amino acid (glutamine/glutamate/aspartate?)		
Q39B19	Bcep18194_B0226	PF03180	D-methionine		
Q39AD9	Bcep18194_B0458	PF04069	glycine betaine/L-proline		
Q39KZ7	Bcep18194_A3267	PF13458	branched-chain amino acid		
Q393K1	Bcep18194_B2254	PF00497	amino acid (glutamine/glutamate/aspartate?)		
Q39IP8	Bcep18194_A4071	PF01547	sugar		
Q39CJ5	Bcep18194_A6227	PF09084	taurine		
Q394P2	Bcep18194_B1961	PF00497	amino acid (glutamine/glutamate/aspartate?)		
Q39A65	Bcep18194_B0532	PF04069	glycine betaine/L-proline		
Q39K66	Bcep18194_A3548	PF00497	amino acid (glutamine/glutamate/aspartate?)		
Q39P52	Bcep18194_C6715	PF00497	amino acid (glutamine/glutamate/aspartate?)		
Q39B17	Bcep18194_B0228	PF00497	amino acid (glutamine/glutamate/aspartate?)		
Q392X7	Bcep18194_B2378	PF00497	amino acid (glutamine/glutamate/aspartate?)		
Q39CQ9	Bcep18194_A6163	PF03180	D-methionine		
Q39JL0	Bcep18194_A3755	PF00497	amino acid (glutamine/glutamate/aspartate?)		
Q39GS6	Bcep18194_A4745	PF13407	ribose		

Q39GX6	Bcep18194_A4695	PF00497	amino acid (glutamine/glutamate/aspartate?)		
Q39C77	Bcep18194_A6345	PF13416	spermidine/putrescine		
Q39KW4	Bcep18194_A3300	PF00497	amino acid (glutamine/glutamate/aspartate?)		
Q39HI9	Bcep18194_A4481	PF00497	amino acid (glutamine/glutamate/aspartate?)		
Q39J28	Bcep18194_A3939	PF00497	amino acid (glutamine/glutamate/aspartate?)		
Q399U6	Bcep18194_B0651	PF13458	branched-chain amino acid		
Q397I9	Bcep18194_B1258	PF00496	dipeptide/oligopeptide		
Q390E1	Bcep18194_B3065	PF01547	sugar		
Q399B1	Bcep18194_B0836	PF00497	amino acid (glutamine/glutamate/aspartate?)		
Q394Y0	Bcep18194_B1867	PF00496	dipeptide/oligopeptide		
Q39AQ6	Bcep18194_B0339	PF13458	branched-chain amino acid		
Q39M19	Bcep18194_C7453	PF00497	amino acid (glutamine/glutamate/aspartate?)		
Q39EN9	Bcep18194_A5483	PF00497	amino acid (glutamine/glutamate/aspartate?)		
Q39NI7	Bcep18194_C6933	PF13416	sugar		
Q39BK0	Bcep18194_B0044	PF00532	ribose		
Q395T6	Bcep18194_B1661	PF13458	branched-chain amino acid		
Q392A8	Bcep18194_B2597	PF00497	amino acid (glutamine/glutamate/aspartate?)		
Q391F7	Bcep18194_B2798	PF04069	glycine betaine/L-proline		
Q393T6	Bcep18194_B2169	PF13416	spermidine/putrescine		
Q398Q8	Bcep18194_B0939	PF01547	sugar		
Q395J0	Bcep18194_B1757	PF09084	nitrate/sulfonate/taurine		
Q391Z9	Bcep18194_B2606	PF09084	phosphonates		
Q39IE5	Bcep18194_A4174	PF03180	D-methionine		
Q39A09	Bcep18194_B0588	PF04069	glycine betaine/L-proline		
Q39LQ7	Bcep18194_C7565	PF13416	spermidine/putrescine		

Q39DR4	Bcep18194_A5808	PF13458	branched-chain amino acid		
Q398C8	Bcep18194_B1069	PF12849	phosphate		
Q39GY6	Bcep18194_A4685	PF09084	nitrate/sulfonate/taurine		
Q39HH9	Bcep18194_A4491	PF01497	cobalamin/Fe3+-siderophores		
Q395I0	Bcep18194_B1767	PF13416	iron(III)		
Q392A3	Bcep18194_B2602	PF09084	phosphonates		
Q39ML8	Bcep18194_C7254	PF12849	phosphate		
Q392I0	Bcep18194_B2525	PF09084	nitrate/sulfonate/taurine		
Q396G3	Bcep18194_B1534	PF00497	amino acid (glutamine/glutamate/aspartate?)		
Q39ED9	Bcep18194_A5583	PF13458	branched-chain amino acid		
Q39GN7	Bcep18194_A4784	PF01497	cobalamin/Fe3+-siderophores		
Q39PN7	Bcep18194_C6528	PF09084	nitrate/sulfonate/taurine		
